# One-pot synthesis of 1,3,5-triazine-2,4-dithione derivatives via three-component reactions

**DOI:** 10.3762/bjoc.16.120

**Published:** 2020-06-24

**Authors:** Gui-Feng Kang, Gang Zhang

**Affiliations:** 1 School of Pharmaceutical Sciences, Capital Medical University, Beijing 100069, China; 2 State Key Laboratory of Bioactive Substances and Function of Natural Medicine, Institute of Materia Medica, Peking Union Medical College and Chinese Academy of Medical Sciences, Beijing 100050, China

**Keywords:** aldehydes, multicomponent reactions, nitrogen heterocycles, thiourea, triazinethiones

## Abstract

A catalyst-free one-pot synthetic methodology was developed for the preparation of 1,3,5-triazine-2,4-dithione derivatives through three-component reactions of arylaldehydes, thiourea, and orthoformates. The procedure tolerated a diverse range of arylaldehydes and orthoformates and provided a rapid entry to a variety of 4-aryl-6-(alkylthio)-3,4-dihydro-1,3,5-triazine-2(1*H*)-thiones (29 examples). The synthetic strategy relies on the dual role of thiourea in the cyclization with the aldehydes and the alkylation via an intermediate imidate formation. The structures of 1,3,5-triazine-2,4-dithione derivatives were characterized by spectroscopic techniques as well as by single crystal X-ray diffraction.

## Introduction

**Figure 1:**
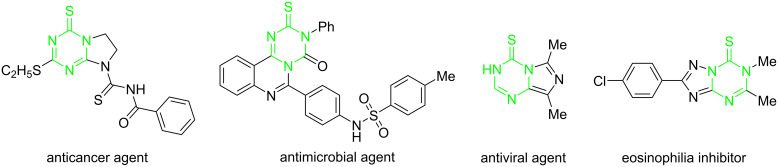
Selected examples of triazinethione-containing bioactive compounds.

The construction of nitrogen-containing heterocycles is one of the most prolific areas in organic chemistry that has drawn considerable attention from chemists due to their widespread occurrence in a variety of natural products [1–4], chemical materials [5–6], and medication [7]. Among the vast array of nitrogen-containing heterocyclic compounds, triazines and substituted triazines are of particular utility in drug discovery because of their broad potential biological activities [8–9]. Besides, triazinethiones also played important roles in the construction of an array of pharmacologically important compounds (Figure 1), comprising anticancer [10–11], antimicrobial [12], antiviral agents [13–14], and eosinophilia inhibitors [15]. Therefore, it is highly desirable to develop efficient and practical synthetic methods for triazinethione architectures and to expand the structure diversity of this class of compounds for medicinal chemistry demands.

**Scheme 1:**
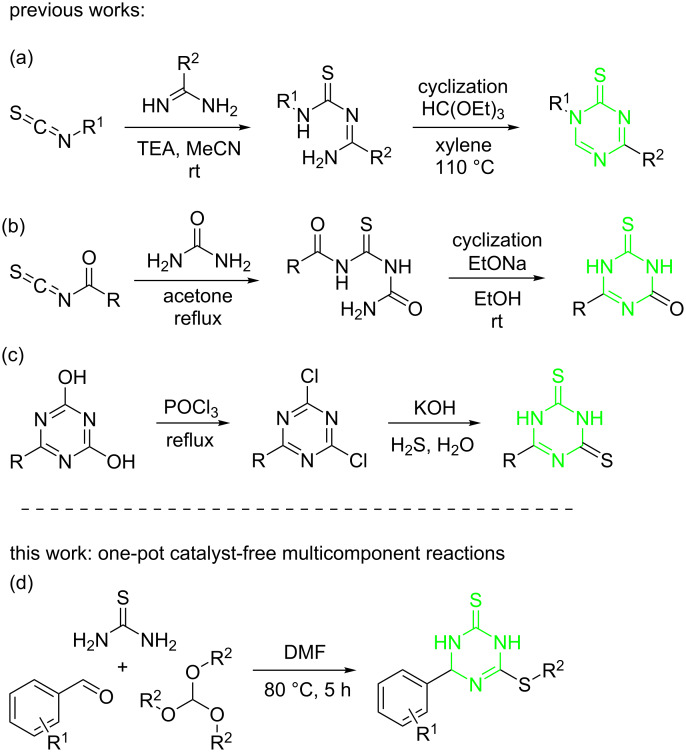
Strategies for the synthesis of triazinethiones.

Several methods were reported for the synthesis of triazinethione analogs. One of the most popular methods involved the reaction of a carboximidamide [16], or urea [17] with isothiocyanates. The C=S bond in the latter is highly polar, with the carbon atom always positive and therefore being susceptible to nucleophilic attack. The resulting substituted thioureas underwent a cyclization reaction providing the desired triazinethiones (Scheme 1a and 1b). However, this method required the preparation of the starting isothiocyanates, which limited its synthetic applications. In another method, triazinethione derivatives were synthesized through the sulfidation of triazine using phosphorus oxychloride and hydrogen sulfide (Scheme 1c) [18]. This strategy required harsh reaction conditions and also suffered from an awful smell. Therefore, the development of facile and environmentally benign methodologies for the generation of structurally diverse triazinethione derivatives is still challenging in organic synthetic chemistry and medicinal chemistry.

Multicomponent reactions (MCRs) gained much interest among synthetic organic chemists as well as in combinatorial chemistry and drug discovery as versatile tools for the construction of different biologically active compounds by combining three or more starting materials into a single product in one convergent chemical step [19–23]. Among multicomponent reactions, aldehydes have emerged as one of the most useful class of molecules. They are quite flexible as possible starting substrates, and through cyclization reactions, can lead to a variety of complex molecules, allowing a wide exploration of scaffolds and substituents diversity [24–28]. In this regard, the use of simple thiourea as an inexpensive thiocarbonyl group source in the multicomponent reaction with aldehydes and other reactive intermediates for the preparation of various triazinethione derivatives by independently varying the individual components thus represents a significant extension in this area. On the other hand, trialkyl orthoformates are versatile building blocks in organic synthesis, capable of constructing acyl derivatives [29–33] and ether derivatives [34–36] through acylation and alkylation under the appropriate conditions.

In view of what was explained above, and as a result of our interest in developing novel multicomponent reactions to access diverse triazinethione derivatives, we recently synthesized a series of 4-aryl-6-(alkylthio)-3,4-dihydro-1,3,5-triazine-2(1*H*)-thiones from aldehydes, thiourea and suitable orthoformates. This strategy was straightforward and the reaction conditions were mild (Scheme 1d). Herein we report the details of this effort.

## Results and Discussion

We explored the synthesis of 1,3,5-triazine-2,4-dithione derivatives by carrying out a set of experiments using benzaldehyde (**1a**, 1.0 equiv), thiourea (**2**, 1.0 equiv), and trimethyl orthoformate (**3a**, 1.0 equiv) as the model reaction (Table 1). The combination of these substrates in the absence of any extra reaction medium did not provide any product at room temperature, even after 12 hours (Table 1, entry 1). Interestingly, when the model reaction was repeated using DMF as the solvent trace amounts of 1,1'-(phenylmethylene)bis(thiourea) (**4**) were observed after stirring 12 h at room temperature (Table 1, entry 2). An increase in the yield of product **4** was observed when the reaction time was extended to 24 h (Table 1, entry 3). Changing the solvent to either hexafluoroisopropanol (HFIP) or 1,4-dioxane did not provide higher yields under the same conditions (Table 1, entries 4 and 5). However, when the reaction temperature was raised to 50 °C in DMF as solvent, the yield of compound **4** decreased (Table 1, entry 6). On the basis of these results, we suggested that upon heating, the inherent reactivity of the intermediate **4** led to a decomposition. In another experiment, the same reaction was tested at 80 °C for 1 h and intermediate **4** in addition to formylthiourea (**5**) were formed in low yields (Table 1, entry 7). When the reaction time was extended to 5 h, the desired three component product 6-(methylthio)-4-phenyl-3,4-dihydro-1,3,5-triazine-2(1*H*)-thione (**6aa**) was obtained in 22% yield (Table 1, entry 8). However, increasing the temperature to 100 °C failed to enhance the reaction rate substantially (Table 1, entry 9). In point of fact, higher temperatures lowered the product yield slightly, accompanied by some impurities.

**Table 1:** Optimization of reaction conditions.^a^

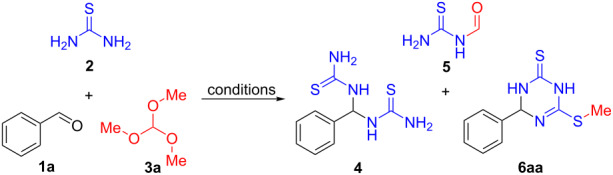						

entry	solvent	*T* (°C)	time (h)	yield (%)^b^		

				**4**	**5**	**6aa**

1	none	rt	12	0	0	0
2	DMF	rt	12	trace	0	0
3	DMF	rt	24	28	0	0
4	HFIP	rt	24	15	0	0
5	dioxane	rt	24	20	0	0
6	DMF	50	24	18	0	0
7	DMF	80	1	20	8	0
8	DMF	80	5	15	17	22
9	DMF	100	5	10	25	16

^a^Reaction conditions: benzaldehyde (1.0 mmol, 1.0 equiv), thiourea (1.0 mmol, 1.0 equiv), trimethyl orthoformate (1.0 mmol, 1.0 equiv); ^b^yields refer to isolated yields.

Encouraged by the result shown in Table 1, entry 8, we further optimized the conditions at 80 °C in DMF as reaction medium by varying the amount of thiourea and trimethyl orthoformate (Table 2). Increasing the amount of thiourea (**2**) significantly increased the yield. If the ratio of **1a**/**2** was altered to 1:2, product **6aa** was isolated in 73% yield (Table 2, entry 1) and we observed that reactions carried out with an excess of **2** gave higher yields of the product (Table 2, entries 2–4). An excellent yield of 90% was achieved when 2.5 equiv of thiourea (**2**) were used (Table 2, entry3). Conversely, increasing the amount of **3a** decreased the yield markedly (Table 2, entries 6–8), and we observed that the best result was obtained with 1.0 equiv of this component relative to **1a** (Table 2, entry 3 vs entries 5–8).

**Table 2:** Optimization of the reaction conditions.^a^

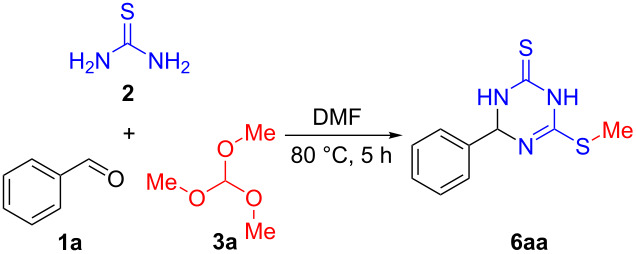			

entry	thiourea (**2**) (equiv)	trimethyl orthoformate (**3a**) (equiv)	yield (%)^b^

1	2.0	1.0	73
2	2.2	1.0	79
3	2.5	1.0	90
4	3.0	1.0	80
5	2.5	0.8	70
6	2.5	1.2	84
7	2.5	1.5	68
8	2.5	2.0	48

^a^Reaction conditions: benzaldehyde (1.0 mmol, 1.0 equiv), thiourea, trimethyl orthoformate, DMF (1 mL), 80 °C, 5 h; ^b^yields refer to isolated yields.

**Scheme 2:**
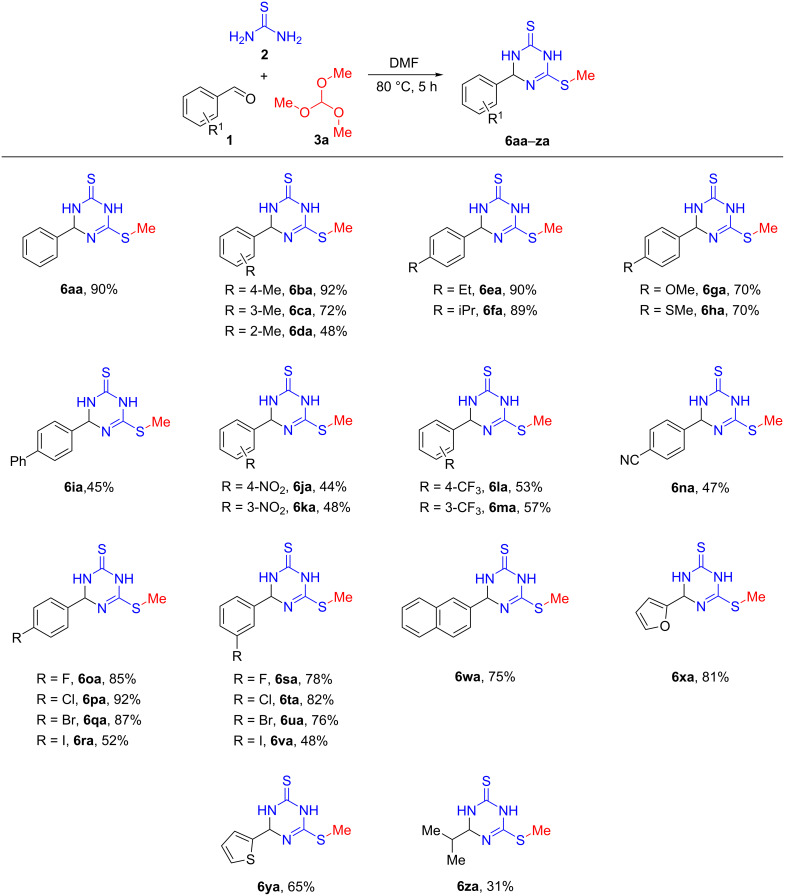
Aldehyde substrate scope of three-component reaction of aldehydes, thiourea and trimethyl orthoformate. Reactions were performed with aldehydes (1.0 mmol, 1.0 equiv), thiourea (2.5 mmol, 2.5 equiv), trimethyl orthoformate (1.0 mmol, 1.0 equiv) in 1 mL DMF at 80 °C for 5 h; yields refer to isolated yields.

Having established the optimal reaction conditions (Table 2, entry 3), we next investigated the generality of this multicomponent reaction with a wide range of aldehydes **1** together with thiourea (**2**) and trimethyl orthoformate (**3a**) (Scheme 2). The reaction carried out with 4-methylbenzaldehyde delivered the expected 6-(methylthio)-4-(*p*-tolyl)-3,4-dihydro-1,3,5-triazine-2(1*H*)-thione (**6ba**) in 92% yield, and this outcome was even better than that of treating the parent compound **1a** analogously. The steric environment surrounding aldehyde group markedly affected the reaction, as 3-methylbenzaldehyde (**1c**) and 2-methylbenzaldehyde (**1d**) provided products **6ca** and **6da** in 72 and 48% yield, respectively. The reaction also worked well with other 4-alkylbenzaldehydes **1e** and **1f**, giving the expected products **6ea** and **6fa** in very good yields. We found benzaldehydes featuring other electron-donating groups such as methoxy (**1g**) and methylthio (**1h**) in the *para*-position of the aldehyde group were also applicable in the reaction, and provided products **6ga** and **6ha** in still moderate yields.

When an electron-deficient aromatic aldehyde such as 4-phenylbenzaldehyde (**1i**) was used, the reaction effectively afforded product **6ia** in 45% yield. Similarly, benzaldehydes having other electron-withdrawing groups such as cyano, nitro, and trifluoromethyl they smoothly took part in the three-component reaction and provided the desired products **6ja–na** in moderate yields. It is noteworthy that benzaldehydes containing a halogen atom such as fluorine, chlorine, bromine, and iodine in the *para* and *meta*-positions were also compatible with the reaction conditions and yielded the corresponding products (**6oa**–**va**, 48–92%), allowing for further functionalization through subsequent transition-metal-catalyzed coupling reactions.

Intriguingly, it was found that compounds with bicycloaryl and heteroaryl ring systems were also viable substrates, affording the desired products **6wa**–**ya** in 65–81% yields. The possibility of using aliphatic aldehydes in place of aromatic aldehydes was investigated for **2** and **3a**. Fortunately, the reactions carried out with isobutyraldehyde (**1z**) yielded 31% of the isopropyl-functionalized 6-(methylthio)-3,4-dihydro-1,3,5-triazine-2(1*H*)-thione (**6za**). However, no product was detected starting from paraformaldehyde and paraldehyde. Presumably, this result could be either due to the aliphatic aldehydes' lower reactivity with thiourea to generate the intermediate imines undergoing ring-closure and subsequent alkylation, and/or due to the instability of the imines formed.

**Scheme 3:**
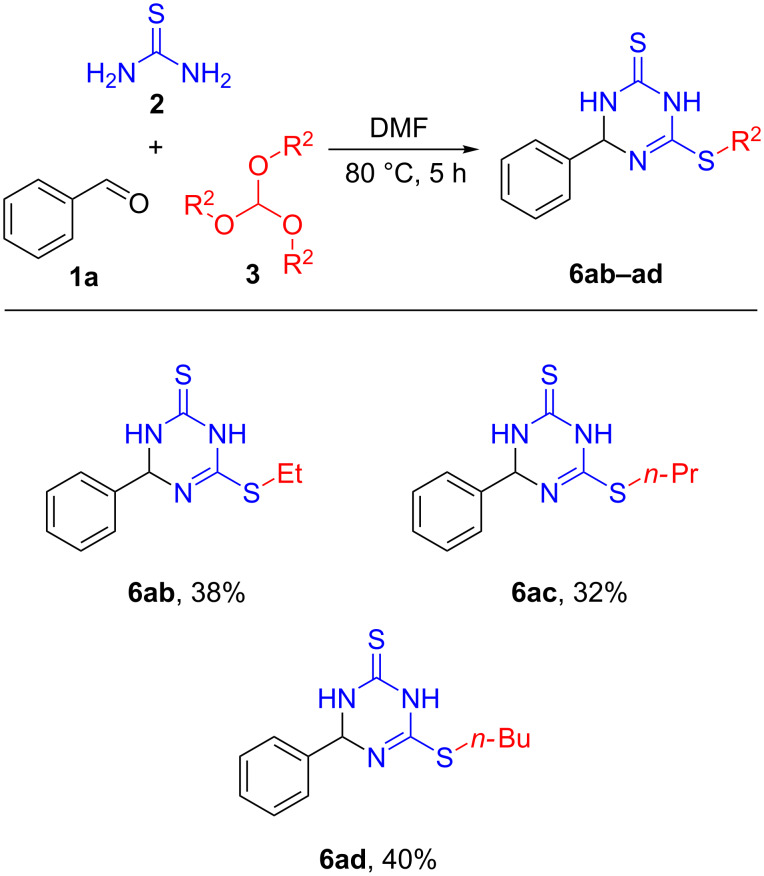
Orthoformate substrate scope of the three component reaction of benzaldehyde, thiourea, and orthoformates. Reactions were performed with benzaldehyde (1.0 mmol, 1.0 equiv), thiourea (2.5 mmol, 2.5 equiv), orthoformates (1.0 mmol, 1.0 equiv) in 1 mL DMF at 80 °C for 5 h; yields refer to isolated yields.

In order to further explore the substrate scope of this multicomponent reaction, next we wanted to use other orthoformates. Interestingly, under the similar reaction conditions triethyl orthoformate (**3b**), tripropyl orthoformate (**3c**), and tributyl orthoformate (**3d**) provided the corresponding products similarly to trimethyl orthoformate and the results are summarized in Scheme 3.

**Scheme 4:**
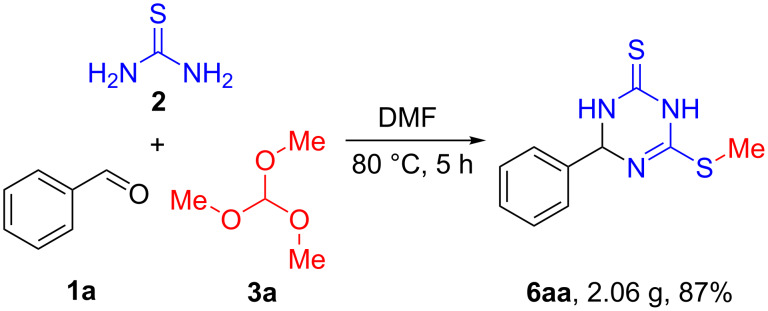
Gram-scale synthesis of **6aa**.

To assess the scalability of the multicomponent reaction, a 10 mmol scale synthesis of 6-(methylthio)-4-phenyl-3,4-dihydro-1,3,5-triazine-2(1*H*)-thione (**6aa**) was conducted under the optimized conditions and the product was obtained in 87% yield (2.06 g). This result was similar to that obtained on a smaller scale (Scheme 4).

**Figure 2:**
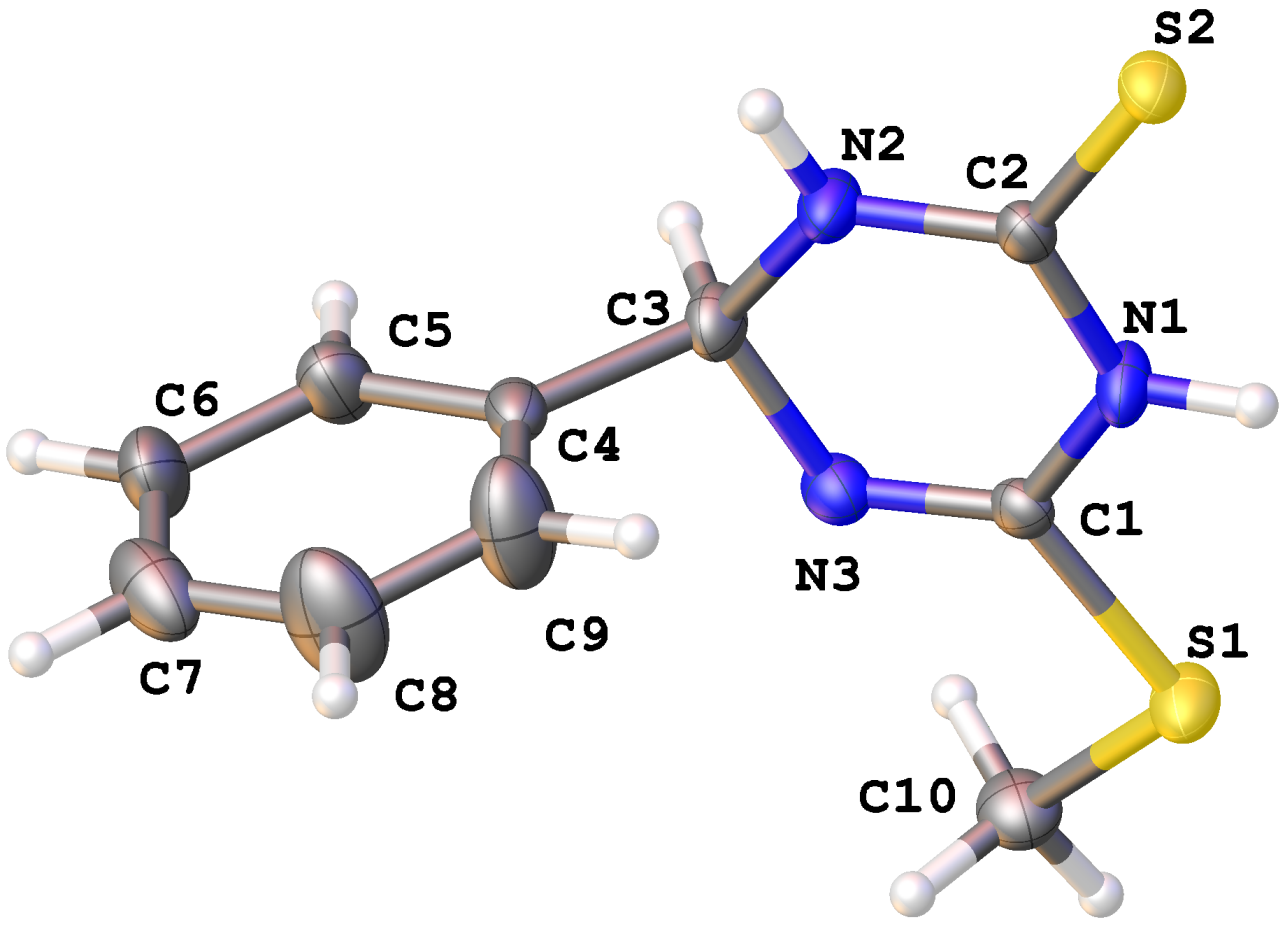
X-ray structure of 6-(methylthio)-4-phenyl-3,4-dihydro-1,3,5-triazine-2(1*H*)-thione (**6aa**) with thermal ellipsoids at 50% probability (CCDC 1991859).

All synthesized compounds were isolated by column chromatography and characterized by detailed spectroscopic analyses. To further verify the structure unambiguously, as a representative example, the structure of **6aa** was also confirmed by single crystal X-ray diffraction (XRD) studies after crystallization from ethyl acetate/hexane (Figure 2). CCDC 1991859 (for **6aa**) contains the supplementary crystallographic data for this paper. These data are provided free of charge by The Cambridge Crystallographic Data Centre.

**Scheme 5:**
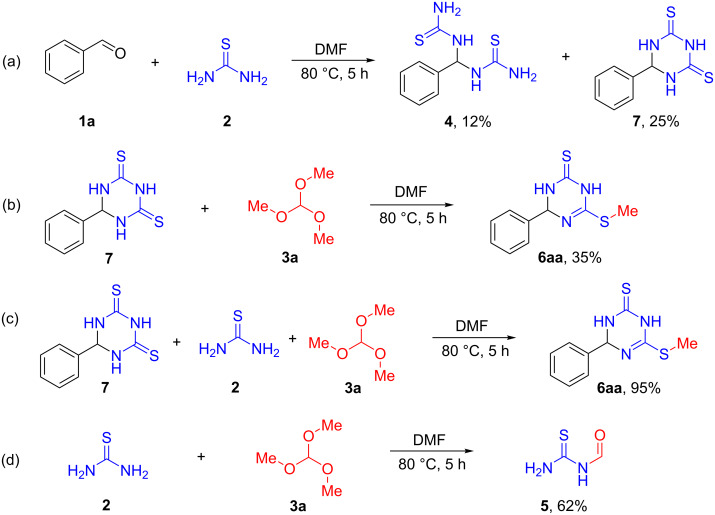
Control experiments for investigation of the mechanism.

To gain insights into the mechanistic pathway, a series of preliminary experiments were performed. First, the reaction of benzaldehyde (**1a**) and thiourea (**2**) in DMF at 80 °C produced 1,1'-(phenylmethylene)bis(thiourea) (**4**) together with its ring closure product **7** (Scheme 5a). It is worth mentioning that reacting compound **7** with trimethyl orthoformate (1.0 equiv) under the optimized conditions for 5 h furnished product **6aa** in only 35% yield (Scheme 5b). On the other hand, repeating the same reaction in the presence of thiourea (1.0 equiv) afforded product **6aa** in 95% yield (Scheme 5c), thereby indicating the crucial participation of thiourea in this alkylation process. Pleasingly, thiourea (**2**) upon heating in DMF with trimethyl orthoformate (**3a**, 1.0 equiv) yielded formylthiourea in 62% yield after 5 h, thus indicating that it is involved in the methylation reaction mechanism (Scheme 5d).

**Scheme 6:**
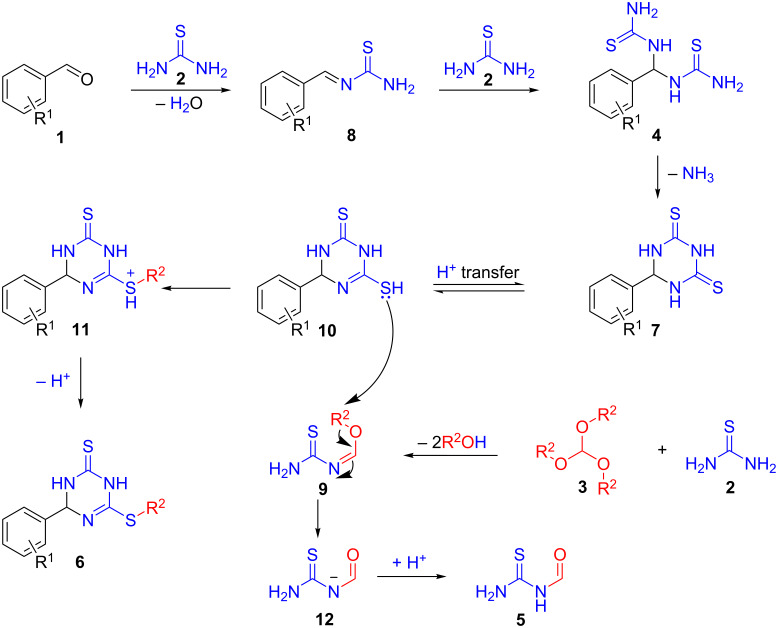
Plausible mechanism.

Based on previous literature reports and our experimental observations, a plausible mechanism for the synthesis of **6** is proposed in Scheme 6. Initially, the reversible nucleophilic attack of thiourea **2** on aldehyde **1** forms imine **8**. Then, a nucleophilic addition of another molecule **2** takes place to furnish the tricomponent adduct **4,** which undergoes elimination of ammonia to afford the corresponding ring-closed intermediate **7** [37]. The latter undergoes a proton-transfer process to form intermediate **10** [38]. Thereafter, the subsequent step involves the reaction of trialkyl orthoformate **3** with thiourea (**2**) to produce imidate intermediate **9**, which is nucleophilically attacked by intermediate **10** and transfers the alkyl group R^2^ to deliver the alkylated intermediate **11**. Meanwhile, *N*,*N*-dimethylformamide dialkyl acetal might also play a role in this alkylation process [39]. Finally, proton transfer processes afford the desired product **6** and byproduct **5**.

## Conclusion

In summary, we have developed a simple and efficient method for the synthesis of 4-aryl-6-(alkylthio)-3,4-dihydro-1,3,5-triazine-2(1*H*)-thiones through a catalyst-free multicomponent reaction. The products were obtained in moderate to good yields by the one-pot reaction of substituted aldehydes, thiourea, and trialkyl orthoformates. The strategy exhibits the use of inexpensive and easily available reagents and substrates to afford the targeted substituted triazinethione derivatives.

## Supporting Information

Supporting information features experimental procedures, characterization data, copies of NMR spectra and crystallographic data.

File 1 Experimental procedures, characterization data, copies of NMR spectra and crystallographic data.

File 2 Crystallographic information file of compound **6aa**.
