# Supplementary material for: One-pot synthesis of 1,3,5-triazine-2,4-dithione derivatives via three-component reactions
Source: Beilstein J Org Chem. 2020 Jun 24;16:1447–55. doi: 10.3762/bjoc.16.120 (PMC7323627; doi:10.3762/bjoc.16.120)
Supplement: File 1 — Experimental procedures, characterization data, copies of NMR spectra and crystallographic data. [file Beilstein_J_Org_Chem-16-1447-s001.pdf]

BEILSTEIN JOURNAL OF ORGANIC CHEMISTRY

Supporting Information

for

One-pot synthesis of 1,3,5-triazine-2,4-dithione derivatives via three-component reactions

Gui-Feng Kang and Gang Zhang

Beilstein J. Org. Chem. **2020,** *16,* 1447–1455. doi:10.3762/bjoc.16.120

Experimental procedures, characterization data, copies of NMR spectra and crystallographic data

License and Terms: This is a supporting information file under the terms of the Creative Commons Attribution License (http://creativecommons.org/licenses/by/4.0). Please note that the reuse, redistribution and reproduction in particular requires that the authors and source are credited.
The license is subject to the *Beilstein Journal of Organic Chemistry* terms and conditions: (https://www.beilstein-journals.org/bjoc)

Table of contents **Pages**

General information....................S2

Synthesis and characterization data....................S2

^{1}H NMR, ^{13}C NMR and ^{19}F NMR spectra of Compounds....................S10

Crystal structure description of **6aa**....................S46

References....................S55

Experimental section

General information: All starting materials were purchased from either Aladdin Chemical Ltd. or J&K Chemical Ltd. and were used without further purification. Reactions were monitored by TLC on Merck silica gel (60 F254) visualized by UV light. Column chromatography was performed by employing Qingdao Haiyang Chemical 200–300 mesh silica gel. NMR spectra were recorded on a Bruker AV300 operating at 300 MHz for ^{1}H, 75 MHz for ^{13}C, and 282 MHz for ^{19}F in DMSO-*d*$_6$. Chemical shift values were reported in δ values in parts per million (ppm), referenced to tetramethylsilane (TMS) or dimethyl sulfoxide (DMSO) by using the residual solvent signal as an internal reference. The HRMS measurements were recorded on an ICR (Fourier transform ion cyclotron resonance, FTICR) analyzer using an ESI source. Infrared spectra (IR) were recorded on a Thermo Scientific Nicolet iS5 FT-IR spectrophotometer and were reported as wavenumbers (cm^{-1}). Melting points were determined with an X-4A microscopic melting point apparatus and are uncorrected.

1,1'-(Phenylmethylene)bis(thiourea) (4)

Benzaldehyde (1.0 mmol) was dissolved in DMF (1.0 mL) in a reaction vial (10 mL). Thiourea (1.0 mmol) nd trimethyl orthoformate (1.0 mmol) were added. The mixture was stirred at rt for 24 h. The solvent was removed under reduced pressure. The residue was purified by chromatography on silica gel (ethyl acetate/petroleum ether 1:10 to 1:1) to give **4**. White solid (67 mg, 28%); m.p. 183.2-185.3 ^{o}C; ^{1}H NMR (300 MHz, DMSO-*d6*) δ = 8.28 (s, 2H, 2NH), 7.41-7.33 (m, 10H); ^{13}C NMR (75 MHz, DMSO-*d6*) δ = 184.3, 182.7, 141.1, 128.7, 128.2, 126.7, 66.4; IR ν_{max} (neat): 3433 (w), 3350 (w), 3234 (br), 3134 (br), 3035 (w), 1605 (vs), 1532 (vs), 1403 (m), 1359 (vs), 1328 (m), 1201 (w), 1150 (m), 1113 (m), 785 (w), 744 (m), 721 (s), 696 (m); HRMS (ESI): *m*/*z* calcd. for $C_9H_{12}N_4S_2$+H$^+$: 241.0576 [*M*+H]$^+$; found: 241.0578.

***N*-Carbamothioylformamide (5)**

Thiourea (1.0 mmol) was dissolved in DMF (1.0 mL) in a reaction vial (10 mL). Trimethyl orthoformate (1.0 mmol) was added. The mixture was stirred at 80 °C for 5 h. The solvent was removed under reduced pressure. The residue was purified by chromatography on silica gel (ethyl acetate/petroleum ether 1:10 to 1:2) to give **5**. White solid (64 mg, 62%); m.p. 169.1-170.8 ^{o}C; ^{1}H NMR (300 MHz, DMSO-*d6*) δ = 10.92 (s, 1H, NH), 9.20-8.11 (m, 3H); ^{13}C NMR (75 MHz, DMSO-*d6*) δ = 182.0, 164.6; IR ν_{max} (neat): 3325 (br), 3160 (br), 3001 (br), 2848 (w), 1672 (m), 1620 (vs), 1531 (m), 1348 (vs), 1213 (vs), 1156 (s), 679 (vs); HRMS (ESI): *m*/*z* calcd. for $C_4H_8N_4O_2S_2$+H$^+$: 209.0161 [2*M*+H]$^+$; found: 209.0161.

General procedure for the synthesis of 4-aryl-6-(alkylthio)-3,4-dihydro-1,3,5-triazine-2(1*H*)-thiones (6aa–za, 6ab–ad): The aldehyde (1.0 mmol) was dissolved in DMF (1.0 mL) in a reaction vial (10 mL). Thiourea (2.5 mmol) and trialkyl orthoformate (1.0 mmol) were added. The mixture was stirred at 80 °C for 5 h. After cooling the reaction mixture to room temperature, the solvent was removed under reduced

pressure. The residue was purified by chromatography on silica gel (ethyl acetate/petroleum ether 1:10 to 1:3) to give the desired product.

6-(Methylthio)-4-phenyl-3,4-dihydro-1,3,5-triazine-2(1*H*)-thione (6aa)

White solid (213 mg, 90%); m.p. 170.3-172.3 °C; ^{1}H NMR (300 MHz, DMSO-*d6*) δ = 11.19 (s, 1H, NH), 9.73 (s, 1H, NH), 7.42-7.29 (m, 5H, Ar-H), 5.80 (s, 1H, CH), 2.28 (s, 3H, SCH_3); ^{13}C NMR (75 MHz, DMSO-*d6*) δ = 174.6, 151.6, 142.1, 128.9, 128.5, 126.5, 70.5, 12.8; IR ν_{max} (neat): 3166 (br), 2962 (w), 1646 (s), 1573 (s), 1469 (m), 1348 (w), 1308 (w), 1187 (s), 1147 (vs), 761 (m), 696 (vs); HRMS (ESI): *m*/*z* calcd. for $C_{10}H_{11}N_3S_2+H^+$: 238.0467 [*M*+H]$^+$; found: 238.0474.

6-(Methylthio)-4-(*p*-tolyl)-3,4-dihydro-1,3,5-triazine-2(1*H*)-thione (6ba)

White solid (231 mg, 92%); m.p. 185.2-187.0 °C; ^{1}H NMR (300 MHz, DMSO-*d6*) δ = 11.15 (s, 1H, NH), 9.69 (s, 1H, NH), 7.19 (m, 4H, Ar-H), 5.75 (s, 1H, CH), 2.29 (s, 3H, SCH_3), 2.28 (s, 3H, CCH_3); ^{13}C NMR (75 MHz, DMSO-*d6*) δ = 174.5, 151.5, 139.3, 137.8, 129.4, 126.4, 70.3, 21.1, 12.8; IR ν_{max} (neat): 3166 (br), 2955 (w), 1651 (vs), 1571 (vs), 1463 (vs), 1180 (vs), 1144 (vs), 843 (m) 812 (vs), 704 (vs); HRMS (ESI): *m*/*z* calcd. for $C_{11}H_{13}N_3S_2+H^+$: 252.0623 [*M*+H]$^+$; found: 252.0629.

6-(Methylthio)-4-(*m*-tolyl)-3,4-dihydro-1,3,5-triazine-2(1*H*)-thione (6ca)

White solid (181 mg, 72%); m.p. 190.0-191.3 °C; ^{1}H NMR (300 MHz, DMSO-*d6*) δ = 11.17 (s, 1H, NH), 9.69 (s, 1H, NH), 7.30-7.25 (m, 1H, Ar-H), 7.15-7.08 (m, 3H, Ar-H), 5.76 (d, J = 2.1 Hz, 1H, CH), 2.31 (s, 3H, CCH_3), 2.28 (s, 3H, SCH_3); ^{13}C NMR (75 MHz, DMSO-*d6*) δ = 174.5, 151.4, 142.1, 138.1, 129.2, 128.9, 127.1, 123.6, 70.5, 21.6, 12.8; IR ν_{max} (neat): 3123 (br), 2976 (w), 2901 (w), 1636 (vs), 1568 (s), 1475 (s), 1315 (w), 1280 (w), 1204 (s), 1161 (s), 1147 (vs), 830 (w), 794 (w), 778 (w), 698 (m), 640 (w); HRMS (ESI): *m*/*z* calcd. for $C_{11}H_{13}N_3S_2+H^+$: 252.0623 [*M*+H]$^+$; found: 252.0624.

6-(Methylthio)-4-(*o*-tolyl)-3,4-dihydro-1,3,5-triazine-2(1*H*)-thione (6da)

White solid (120 mg, 48%); m.p. 197.0-198.4 °C; ^{1}H NMR (300 MHz, DMSO-*d6*) δ = 11.18 (s, 1H, NH), 9.57 (s, 1H, NH), 7.25-7.14 (m, 4H, Ar-H), 5.96 (d, J = 1.5 Hz, 1H, CH), 2.41 (s, 3H, CCH_3), 2.23 (s, 3H, SCH_3); ^{13}C NMR (75 MHz, DMSO-*d6*) δ = 175.2, 151,1, 139.7, 135.9, 131.1, 128.4, 126.4, 126.1, 68.9, 19.1, 12.7; IR ν_{max} (neat): 3095 (br), 2960 (br), 1646 (s), 1563 (vs), 1470 (s), 1319 (v), 1177 (s), 1153 (vs), 1069 (m), 832 (m), 748 (vs), 721 (m), 639 (m); HRMS (ESI): *m*/*z* calcd. for $C_{11}H_{13}N_3S_2+H^+$: 252.0623 [*M*+H]$^+$; found: 252.0628.

4-(4-Ethylphenyl)-6-(methylthio)-3,4-dihydro-1,3,5-triazine-2(1*H*)-thione (6ea)

White solid (238 mg, 90%); m.p. 170.1-172.0 °C; ^{1}H NMR (300 MHz, DMSO-*d6*) δ = 11.16 (s, 1H, NH), 9.70 (s, 1H, NH), 7.22 (m, 4H, Ar-H), 5.76 (d, J = 1.5 Hz, 1H, CH), 2.59 (q, J = 7.5 Hz, 2H, CH2), 2.28 (s, 3H, SCH3), 1.16 (t, J = 7.5 Hz, 3H, CH3); ^{13}C NMR (75 MHz, DMSO-*d6*) δ = 174.5, 151.5, 144.2, 139.6, 128.3, 126.5, 70.3, 28.3, 16.1, 12.8; IR ν_{max} (neat): 3158 (br), 2959 (w), 2927 (w), 1650 (vs), 1573 (s), 1462 (s), 1338 (w), 1307 (w), 1278 (w), 1186 (s), 1146 (vs), 826 (vs), 706 (s); HRMS (ESI): *m*/*z* calcd. for $C_{12}H_{15}N_3S_2$+Na^+: 288.0599 [*M*+Na]$^+$; found: 288.0604.

4-(4-Isopropylphenyl)-6-(methylthio)-3,4-dihydro-1,3,5-triazine-2(1*H*)-thione (6fa)

White solid (248 mg, 89%); m.p. 170.2-171.5 °C; ^{1}H NMR (300 MHz, DMSO-*d6*) δ = 11.15 (s, 1H, NH), 9.68 (s, 1H, NH), 7.28-7.20 (m, 4H, Ar-H), 5.76 (d, J = 1.8 Hz, 1H, CH), 2.88 (m, J = 6.9 Hz, 1H, CH(CH3)2), 2.28 (s, 3H, SCH3), 1.19 (d, J = 6.9 Hz, 6H, 2CH3); ^{13}C NMR (75 MHz, DMSO-*d6*) δ = 174.3, 151.4, 148.8, 139.7, 126.8, 126.5, 70.2, 33.6, 24.3, 12.8; IR ν_{max} (neat): 3151 (br), 2959 (w), 1643 (s), 1567 (s), 1464 (s), 1381 (w), 1359 (w), 1288 (w), 1154 (vs), 834 (m), 822 (m), 700 (s), 641 (m); HRMS (ESI): *m*/*z* calcd. for $C_{26}H_{34}N_6S_4$+Na^+: 581.1620 [2*M*+Na]$^+$; found: 581.1621.

4-(4-Methoxyphenyl)-6-(methylthio)-3,4-dihydro-1,3,5-triazine-2(1*H*)-thione (6ga)

White solid (187 mg, 70%); m.p. 167.3-169.0 °C; ^{1}H NMR (300 MHz, DMSO-*d6*) δ = 11.15 (s, 1H, NH), 9.67 (s, 1H, NH), 7.21 (d, J = 8.7 Hz, 2H, Ar-H), 6.74 (d, J = 8.7 Hz, 2H, Ar-H), 5.74 (s, 1H, CH), 3.74 (s, 3H, OCH3), 2.28 (s, 3H, SCH3); ^{13}C NMR (75 MHz, DMSO-*d6*) δ = 174.5, 159.5, 151.4, 134.4, 127.8, 114.3, 70.1, 55.6, 12.8; IR ν_{max} (neat): 3162 (br), 2966 (w), 1650 (vs), 1614 (m), 1574 (vs), 1515 (s), 1461 (s), 1252 (s), 1190 (vs), 1148 (vs), 1030 (s), 838 (s), 818 (s), 724 (m); HRMS (ESI): *m*/*z* calcd. for $C_{22}H_{26}N_6O_2S_4$+Na^+: 557.0892 [2*M*+Na]$^+$; found: 557.0897.

6-(Methylthio)-4-(4-(methylthio)phenyl)-3,4-dihydro-1,3,5-triazine-2(1*H*)-thione (6ha)

White solid (198 mg, 70%); m.p. 180.0-181.3 °C; ^{1}H NMR (300 MHz, DMSO-*d6*) δ = 11.19 (s, 1H, NH), 9.71 (s, 1H, NH), 7.30-7.22 (m, 4H, Ar-H), 5.77 (d, J = 2.1 Hz, 1H, CH), 2.47 (s, 3H, SCH3), 2.28 (s, 3H, SCH3); ^{13}C NMR (75 MHz, DMSO-*d6*) δ = 174.6, 151.7, 138.8, 138.6, 127.1, 126.4, 70.1, 15.1, 12.8; IR ν_{max} (neat): 3174 (br), 2922 (w), 1651 (m), 1567 (s), 1461 (m), 1309 (w), 1181 (sm), 1145 (vs), 814 (vs), 703 (m), 638 (w); HRMS (ESI): *m*/*z* calcd. for $C_{11}H_{13}N_3S_3$-H^-: 282.0188 [*M*-H]$^-$; found: 282.0199.

4-([1,1'-Biphenyl]-4-yl)-6-(methylthio)-3,4-dihydro-1,3,5-triazine-2(1*H*)-thione (6ia)

White solid (141 mg, 45%); m.p. 208.2-210.0 °C; ^{1}H NMR (300 MHz, DMSO-*d6*) δ = 11.23 (s, 1H, NH), 9.79 (s, 1H, NH), 7.70-7.34 (m, 9H, Ar-H), 5.86 (d, J = 1.2 Hz, 1H, CH), 2.31 (s, 3H, SCH3); ^{13}C NMR (75 MHz, DMSO-*d6*) δ = 174.6, 151.8, 141.2, 140.5, 140.1, 129.4, 128.0, 127.3, 127.2, , 127.1, 70.2, 12.8; IR

ν_{max} (neat): 3137 (br), 2974 (w), 1647 (vs), 1585 (m), 1573 (vs), 1473 (vs), 1197 (s), 1182 (s), 1157 (vs), 1075 (m), 832 (s), 748 (s), 724 (s), 691 (vs); HRMS (ESI): *m*/*z* calcd. for $C_{16}H_{15}N_3S_2+H^+$: 314.0780 [*M*+H]$^+$; found: 314.0785.

6-(Methylthio)-4-(4-nitrophenyl)-3,4-dihydro-1,3,5-triazine-2(1*H*)-thione (6ja)

White solid (124 mg, 44%); m.p. 207.5-209.3 °C; ^{1}H NMR (300 MHz, DMSO-*d6*) δ = 11.35 (s, 1H, NH), 9.88 (s, 1H, NH), 8.28 (d, J = 8.7 Hz, 2H, Ar-H), 7.59 (d, J = 8.7 Hz, 2H, Ar-H), 5.98 (s, 1H, CH), 2.30 (s, 3H, SCH3); ^{13}C NMR (75 MHz, DMSO-*d6*) δ = 174.8, 152.7, 148.8, 147.7, 128.0, 124.3, 69.6, 12.9; IR ν_{max} (neat): 3156 (br), 2953 (w), 1651 (m), 1571 (m), 1512 (s), 1468 (m), 1349 (s), 1186 (s), 1145 (vs), 840 (m), 817 (m), 696 (s); HRMS (ESI): *m*/*z* calcd. for $C_{10}H_{10}N_4O_2S_2-H^-$: 281.0161 [*M*-H]$^-$; found: 281.0168.

6-(Methylthio)-4-(3-nitrophenyl)-3,4-dihydro-1,3,5-triazine-2(1*H*)-thione (6ka)

White solid (135 mg, 48%); m.p. 192.5-194.5 °C; ^{1}H NMR (300 MHz, DMSO-*d6*) δ = 11.36 (s, 1H, NH), 9.88 (s, 1H, NH), 8.22-8.18 (m, 2H, Ar-H), 7.80-7.69 (m, 2H, Ar-H), 6.01 (s, 1H, CH), 2.32 (s, 3H, SCH3); ^{13}C NMR (75 MHz, DMSO-*d6*) δ = 174.9, 152.8, 148.2, 143.9, 133.4, 130.7, 123.6, 121.4, 69.3, 12.9; IR ν_{max} (neat): 3096 (br), 2970 (w), 1634 (m), 1574 (m), 1532 (s), 1470 (m), 1344 (m), 1194 (s), 1155 (vs), 832 (m), 726 (vs), 642 (m); HRMS (ESI): *m*/*z* calcd. for $C_{10}H_{10}N_4O_2S_2-H^-$: 281.0161 [*M*-H]$^-$; found: 281.0172.

6-(Methylthio)-4-(4-(trifluoromethyl)phenyl)-3,4-dihydro-1,3,5-triazine-2(1*H*)-thione (6la)

White solid (162 mg, 53%); m.p. 206.9-208.0 °C; ^{1}H NMR (300 MHz, DMSO-*d6*) δ = 11.30 (s, 1H, NH), 9.83 (s, 1H, NH), 7.78 (d, J = 8.1 Hz, 2H, Ar-H), 7.54 (d, J = 8.1 Hz, 2H, Ar-H), 5.93 (d, J = 1.5 Hz, 1H, CH), 2.30 (s, 3H, SCH3); ^{13}C NMR (75 MHz, DMSO-*d6*) δ = 174.8, 152.4, 146.3, 129.1 (q, J = 31.5 Hz), 127.5, 126.01 (q, J = 3.75 Hz), 124.6 (q, J = 270.75 Hz), 69.8, 12.8; ^{19}F NMR (282 MHz, DMSO-*d6*) δ = -60.9; IR ν_{max} (neat): 3156 (br), 3094 (w), 2952 (w), 2884 (w), 1651 (s), 1571 (vs), 1464 (s), 1422 (m), 1328 (vs), 1282 (w), 1188 (m), 1147 (vs), 1109 (vs), 1064 (s), 1017 (w), 833 (s), 703 (w), 609 (w); HRMS (ESI): *m*/*z* calcd. for $C_{11}H_{10}F_3N_3S_2+H^+$: 306.0341 [*M*+H]$^+$; found: 306.0350.

6-(Methylthio)-4-(3-(trifluoromethyl)phenyl)-3,4-dihydro-1,3,5-triazine-2(1*H*)-thione (6ma)

White solid (174 mg, 57%); m.p. 191.2-193.0 °C; ^{1}H NMR (300 MHz, DMSO-*d6*) δ = 11.32 (s, 1H, NH), 9.82 (s, 1H, NH), 7.73-7.64 (m, 4H, Ar-H), 5.96 (s, 1H, CH), 2.30 (s, 3H, SCH3); ^{13}C NMR (75 MHz, DMSO-*d6*) δ = 174.9, 152.5, 143.2, 130.84, 130.27, 129.5 (q, J = 31.5 Hz), 125.4 (q, J = 3.75 Hz), 124.5 (q, J = 270.75 Hz), 123.3 (q, J = 3.75 Hz), 69.7, 12.8; ^{19}F NMR (282 MHz, DMSO-*d6*) δ = -61.0; IR ν_{max} (neat): 3163 (br), 3093 (br), 2956 (w), 1651 (vs), 1565 (vs), 1453 (s), 1415 (s), 1324 (vs), 1265 (s), 1163 (vs), 1141 (s), 1110 (s), 1097 (s), 1065 (vs), 847 (m), 810 (s), 732 (m), 701 (m); HRMS (ESI): *m*/*z* calcd. for $C_{22}H_{20}F_6N_6S_4+Na^+$: 328.0160 [2*M*+Na]$^+$; found: 328.0158.

4-(4-(Methylthio)-6-thioxo-1,2,5,6-tetrahydro-1,3,5-triazin-2-yl)benzonitrile (6na)

White solid (123 mg, 47%); m.p. 210.0-211.8 °C; ^{1}H NMR (300 MHz, DMSO-*d6*) δ = 11.31 (s, 1H, NH), 9.82 (s, 1H, NH), 7.89 (d, J = 8.4 Hz, 2H, Ar-H), 7.51 (d, J = 8.1 Hz, Ar-H), 5.92 (d, J = 1.8 Hz, 1H, CH), 2.30 (s, 3H, SCH3); ^{13}C NMR (75 MHz, DMSO-*d6*) δ = 174.9, 152.6, 147.0, 133.1, 127.6, 119.1, 111.4, 69.8, 12.8; IR ν_{max} (neat): 3165 (br), 3086 (w), 2948 (w), 2230 (m), 1651 (vs), 1568 (vs), 1461 (s), 1411 (m), 1341 (w), 1302 (w), 1278 (w), 1212 (w), 1185 (vs), 1144 (vs), 828 (vs), 711 (m), 697 (m); HRMS (ESI): *m*/*z* calcd. for $C_{22}H_{20}N_8S_4+Na^+$: 547.0585 [2*M*+Na]$^+$; found: 547.0588.

4-(4-Fluorophenyl)-6-(methylthio)-3,4-dihydro-1,3,5-triazine-2(1*H*)-thione (6oa)

White solid (217 mg, 85%); m.p. 192.3-193.5 °C; ^{1}H NMR (300 MHz, DMSO-*d6*) δ = 11.23 (s, 1H, NH), 9.74 (s, 1H, NH), 7.37-7.32 (m, 2H, Ar-H), 7.26-7.19 (m, 2H, Ar-H), 5.82 (d, J = 1.8 Hz, 1H, CH), 2.29 (s, 3H, SCH3); ^{13}C NMR (75 MHz, DMSO-*d6*) δ = 174.6, 162.2 (d, J = 242.25 Hz), 151.9, 138.4 (d, J = 3.0 Hz), 128.7 (d, J = 8.25 Hz), 115.7 (d, J = 21.75 Hz), 69.7, 12.8; ^{19}F NMR (282 MHz, DMSO-*d6*) δ = -114.0; IR ν_{max} (neat): 3168 (br), 3091 (w), 2950 (w), 2875 (w), 1655 (s), 1571 (vs), 1507 (m), 1461 (s), 1335 (w), 1308 (w), 1276 (w), 1223 (m), 1181 (m), 1144 (vs), 829 (m), 706 (w), 647 (w); HRMS (ESI): *m*/*z* calcd. for $C_{10}H_{10}FN_3S_2+H^+$: 256.0372 [*M*+H]$^+$; found: 256.0379.

4-(4-Chlorophenyl)-6-(methylthio)-3,4-dihydro-1,3,5-triazine-2(1*H*)-thione (6pa)

White solid (249 mg, 92%); m.p. 194.1-195.7 °C; ^{1}H NMR (300 MHz, DMSO-*d6*) δ = 11.25 (s, 1H, NH), 9.76 (s, 1H, NH), 7.47 (d, J = 8.4 Hz, 2H, Ar-H), 7.32 (d, J = 8.4 Hz, 2H, Ar-H), 5.82 (s, 1H, CH), 2.28 (s, 3H, SCH3); ^{13}C NMR (75 MHz, DMSO-*d6*) δ = 174.7, 152.1, 141.0, 133.2, 129.0, 128.5, 69.7, 12.8; IR ν_{max} (neat): 3169 (br), 2953 (w), 1645 (s), 1569 (vs), 1464 (s), 1332 (w), 1211 (w), 1145 (vs), 1085 (s), 821 (vs), 705 (m); HRMS (ESI): *m*/*z* calcd. for $C_{10}H_{10}ClN_3S_2-H^-$: 269.9920 [*M*-H]$^-$; found: 269.9932.

4-(4-Bromophenyl)-6-(methylthio)-3,4-dihydro-1,3,5-triazine-2(1*H*)-thione (6qa)

White solid (275 mg, 87%); m.p. 202.9-204.0 °C; ^{1}H NMR (300 MHz, DMSO-*d6*) δ = 11.25 (s, 1H, NH), 9.75 (s, 1H, NH), 7.60 (d, J = 8.4 Hz, 2H, Ar-H), 7.26 (d, J = 8.4 Hz, 2H, Ar-H), 5.81 (s, 1H, CH), 2.29 (s, 3H, SCH3); ^{13}C NMR (75 MHz, DMSO-*d6*) δ = 174.7, 152.1, 141.4, 131.9, 128.8, 121.8, 69.8, 12.8; IR ν_{max} (neat): 3163 (br), 2955 (w), 1651 (vs), 1567 (vs), 1463 (vs), 1331 (w), 1280 (w), 1210 (m), 1187 (vs), 1145 (vs), 1071 (m), 1007 (m), 830 (m), 818 (vs), 702 (s); HRMS (ESI): *m*/*z* calcd. for $C_{10}H_{10}BrN_3S_2+Na^+$: 337.9391 [*M*+Na]$^+$; found: 337.9398.

4-(4-Iodophenyl)-6-(methylthio)-3,4-dihydro-1,3,5-triazine-2(1*H*)-thione (6ra)

White solid (189 mg, 52%); m.p. 202.7-204.8 °C; ^{1}H NMR (300 MHz, DMSO-*d6*) δ = 11.22 (s, 1H, NH), 9.73 (s, 1H, NH), 7.77 (d, J = 8.1 Hz, 2H, Ar-H), 7.11 (d, J = 8.1 Hz, 2H, Ar-H), 5.78 (d, J = 1.5 Hz, 1H, CH),

2.28 (s, 3H, CH3); ^{13}C NMR (75 MHz, DMSO-*d6*) δ = 174.4, 151.9, 141.8, 137.7, 128.9, 94.9, 69.8, 12.8; IR $ν_{max}$ (neat): 3165 (br), 2958 (w), 1650 (s), 1571 (s), 1461 (s), 1330 (w), 1280 (w), 1187 (s), 1146 (vs), 818 (vs), 704 (m), 655 (w); HRMS (ESI): *m*/*z* calcd. for $C_{10}H_{10}IN_3S_2$-H^-: 361.9277 [*M*-H]$^-$; found: 361.9285.

4-(3-Fluorophenyl)-6-(methylthio)-3,4-dihydro-1,3,5-triazine-2(1*H*)-thione (6sa)

White solid (199 mg, 78%); m.p. 196.0-197.8 ^oC; ^1H NMR (300 MHz, DMSO-*d6*) δ = 11.26 (s, 1H, NH), 9.77 (s, 1H, NH), 7.49-7.08 (m, 4H, Ar-H), 5.85 (d, J = 1.8 Hz, 1H, CH), 2.30 (s, 3H, SCH3); ^{13}C NMR (75 MHz, DMSO-*d6*) δ = 174.8, 162.5 (d, J = 242.25 Hz), 152.2, 144.8 (d, J = 6.0 Hz), 131.1 (d, J = 7.5 Hz), 122.6 (d, J = 3.0 Hz), 115.4 (d, J = 21.0 Hz), 113.4 (d, J = 21.75 Hz), 69.7 (d, J = 1.50 Hz), 12.8; ^{19}F NMR (282 MHz, DMSO-*d6*) δ = -112.6; IR $ν_{max}$ (neat): 3120 (br), 2976 (m), 1640 (s), 1569 (vs), 1470 (vs), 1324 (v), 1205 (vs), 1163 (vs), 1126 (m), 867 (v), 836 (s), 727 (s), 682 (vs); HRMS (ESI): *m*/*z* calcd. for $C_{10}H_{10}FN_3S_2$+Na^+: 278.0192 [*M*+Na]$^+$; found: 278.0194.

4-(3-Chlorophenyl)-6-(methylthio)-3,4-dihydro-1,3,5-triazine-2(1*H*)-thione (6ta)

White solid (222 mg, 82%); m.p. 195.0-195.7 ^oC; ^1H NMR (300 MHz, DMSO-*d6*) δ = 11.28 (s, 1H, NH), 9.78 (s, 1H, NH), 7.47-7.39 (m, 2H, Ar-H), 7.34-7.26 (m, 2H, Ar-H), 5.84 (d, J = 2.1 Hz, 1H, CH), 2.30 (s, 3H, SCH3); ^{13}C NMR (75 MHz, DMSO-*d6*) δ = 174.8, 152.3, 144.4, 133.5, 131.0, 128.5, 126.5, 125.3, 69.7, 12.8; IR $ν_{max}$ (neat): 3120 (br), 2978 (w), 2897 (w), 1636 (vs), 1581 (vs), 1471 (s), 1319 (w), 1280 (w), 1192 (m), 1154 (s), 1075 (m), 830 (w), 786 (w), 731 (w), 690 (w), 642 (w); HRMS (ESI): *m*/*z* calcd. for $C_{20}H_{20}Cl_2N_6S_4$+Na^+: 564.9901 [2*M*+Na]$^+$; found: 564.9907.

4-(3-Bromophenyl)-6-(methylthio)-3,4-dihydro-1,3,5-triazine-2(1*H*)-thione (6ua)

White solid (240 mg, 76%); m.p. 201.4-202.2 ^oC; ^1H NMR (300 MHz, DMSO-*d6*) δ = 11.27 (s, 1H, NH), 9.76 (s, 1H, NH), 7.55-7.30 (m, 4H, Ar-H), 5.84 (d, J = 1.2 Hz, 1H, CH), 2.30 (s, 3H, SCH3); ^{13}C NMR (75 MHz, DMSO-*d6*) δ = 174.7, 152.3, 144.6, 131.4, 131.3, 129.4, 125.7, 122.1, 69.6, 12.8; IR $ν_{max}$ (neat): 3121 (br), 2977 (w), 2906 (w), 1638 (vs), 1579 (s), 1471 (s), 1423 (s), 1318 (w), 1280 (w), 1188 (s), 1152 (vs), 1074 (s), 829 (s), 785 (s), 728 (s), 689 (vs); HRMS (ESI): *m*/*z* calcd. for $C_{20}H_{20}Br_2N_6S_4$+Na^+: 654.8870 [2*M*+Na]$^+$; found: 654.8861.

4-(3-Iodophenyl)-6-(methylthio)-3,4-dihydro-1,3,5-triazine-2(1*H*)-thione (6va)

White solid (174 mg, 48%); m.p. 201.9-203.2 ^oC; ^1H NMR (300 MHz, DMSO-*d6*) δ = 11.26 (s, 1H, NH), 9.75 (s, 1H, NH), 7.72-7.66 (m, 2H, Ar-H), 7.34-7.31 (m, 1H, Ar-H), 7.24-7.18 (m, 1H, Ar-H), 5.80 (d, J = 1.8 Hz, 1H, CH), 2.29 (s, 3H, SCH3); ^{13}C NMR (75 MHz, DMSO-*d6*) δ = 174.7, 152.1, 144.5, 137.3, 135.3, 131.3, 126.0, 95.3, 69.6, 12.8; IR $ν_{max}$ (neat): 3131 (br), 2972 (w), 2908 (w), 1637 (vs), 1573 (vs), 1467 (s), 1418 (m), 1317 (w), 1278 (w), 1184 (s), 1149 (vs), 1072 (v), 828 (m), 786 (m), 723 (w), 689 (m), 644 (w); HRMS (ESI): *m*/*z* calcd. for $C_{10}H_{10}IN_3S_2$-H^-: 361.9277 [*M*-H]$^-$; found: 361.9288.

6-(Methylthio)-4-(naphthalen-2-yl)-3,4-dihydro-1,3,5-triazine-2(1*H*)-thione (6wa)

White solid (215 mg, 75%); m.p. 197.5-199.1 ^oC; ^1H NMR (300 MHz, DMSO-*d6*) δ = 11.26 (s, 1H, NH), 9.83 (s, 1H, NH), 7.97-7.90 (m, 3H, Ar-H), 7.79 (m ,1H, Ar-H), 7.56-7.47 (m, 3H, Ar-H), 5.99 (s, 1H, CH), 2.31 (s, 3H, SCH3); ^{13}C NMR (75 MHz, DMSO-*d6*) δ = 174.7, 151.8, 139.5, 133.1, 132.9, 128.9, 128.5, 128.0, 126.9, 126.8, 125.1, 124.8, 70.8, 12.8; IR v_{max} (neat): 3117 (br), 2974 (w), 2921 (w), 2897 (w), 1639 (vs), 1573 (m), 1473 (m), 1295 (w), 1205 (s), 1152 (vs), 813 (m), 750 (m), 735 (s), 645 (m); HRMS (ESI): *m*/*z* calcd. for $C_{14}H_{13}N_3S_2$-H^-: 286.0467 [*M*-H]$^-$; found: 286.0478.

4-(Furan-2-yl)-6-(methylthio)-3,4-dihydro-1,3,5-triazine-2(1*H*)-thione (6xa)

White solid (184 mg, 81%); m.p. 187.5-189.4 ^oC; ^1H NMR (300 MHz, DMSO-*d6*) δ = 11.27 (s, 1H, NH), 9.72 (s, 1H, NH), 7.65-7.64 (m, 1H, Ar-H), 6.43-6.42 (m, 1H, Ar-H), 6.31-6.30 (m, 1H, Ar-H), 5.83 (d, J = 2.1 Hz, 1H, CH), 2.27 (s, 3H, SCH3); ^{13}C NMR (75 MHz, DMSO-*d6*) δ = 174.9, 153.4, 153.0, 143.6, 110.8, 107.4, 64.6, 12.8; IR v_{max} (neat): 3145 (br), 2959 (w), 1643 (s), 1567 (s), 1469 (m), 1310 (w), 1235 (m), 1145 (vs), 728 (s), 637 (m); HRMS (ESI): *m*/*z* calcd. for $C_{16}H_{18}N_6O_2S_4$+Na^+: 477.0266 [2*M*+Na]$^+$; found: 477.0271.

6-(Methylthio)-4-(thiophen-2-yl)-3,4-dihydro-1,3,5-triazine-2(1*H*)-thione (6ya)

White solid (158 mg, 65%); m.p. 179.0-180.1 ^oC; ^1H NMR (300 MHz, DMSO-*d6*) δ = 11.31 (s, 1H, NH), 9.90 (s, 1H, NH), 7.48-7.47 (m, 1H, Ar-H), 7.06-6.99 (m, 2H, Ar-H), 6.05 (d, J = 2.4 Hz, 1H, CH), 2.31 (s, 3H, SCH3); ^{13}C NMR (75 MHz, DMSO-*d6*) δ = 174.5, 152.9, 146.1, 127.2, 126.3, 125.0, 66.4, 12.9; IR v_{max} (neat): 3128 (br), 2968 (w), 2926 (w), 1635 (s), 1573 (vs), 1471 (m), 1293 (w), 1208 (s), 1152 (vs), 831 (m), 703 (vs), 640 (s); HRMS (ESI): *m*/*z* calcd. for $C_8H_9N_3S_3$+Na^+: 265.9850 [*M*+Na]$^+$; found: 265.9857.

4-Isopropyl-6-(methylthio)-3,4-dihydro-1,3,5-triazine-2(1*H*)-thione (6za)

White solid (63 mg, 31%); m.p. 179.3-181.3 ^oC; ^1H NMR (300 MHz, DMSO-*d6*) δ = 10.88 (s, 1H, NH), 9.16 (s, 1H, NH), 4.57-4.55 (dd, J1 = 1.5 Hz, J2 = 3.9 Hz, 1H, CH), 2.27 (s, 3H, SCH3), 1.88-1.78 (m, 1H, CH(CH3)2), 0.90 (d, J = 6.9 Hz, 3H, CH3), 0.80 (d, J = 6.9 Hz, 3H, CH3); ^{13}C NMR (75 MHz, DMSO-*d6*) δ = 175.4, 150.8, 73.5, 34.4, 17.7, 16.2, 12.6; IR v_{max} (neat): 3173 (br), 2959 (s), 2930 (m), 2871 (w), 1724 (w), 1655 (vs), 1576 (vs), 1464 (m), 1380 (m), 1331 (w), 1309 (w), 1278 (w), 1203 (m), 1150 (m), 1131 (m); HRMS (ESI): *m*/*z* calcd. for $C_{14}H_{26}N_6S_4$+Na^+: 429.0994 [2*M*+Na]$^+$; found: 429.0994.

6-(Ethylthio)-4-phenyl-3,4-dihydro-1,3,5-triazine-2(1*H*)-thione (6ab)

White solid (95 mg, 38%); m.p. 144.8-145.7 ^oC; ^1H NMR (300 MHz, DMSO-*d6*) δ = 11.12 (s, 1H, NH), 9.72 (s, 1H, NH), 7.43-7.29 (m, 5H, Ar-H), 5.79 (d, J = 2.1 Hz, 1H, CH), 2.99-2.79 (m, 2H, CH2), 1.18 (t, J = 7.2 Hz, 3H, CH3); ^{13}C NMR (75 MHz, DMSO-*d6*) δ = 174.7, 150.8, 142.1, 128.9, 128.6, 126.5, 70.4, 24.1, 14.9;

IR ν_{max} (neat): 3433 (br), 3129 (br), 2981 (w), 2850 (br), 1622 (m), 1582 (s), 1491 (m), 1378 (w), 1216 (s), 1170 (vs), 823 (s), 714 (vs), 692 (s), 653 (s); HRMS (ESI): *m*/*z* calcd. for $C_{11}H_{13}N_3S_2+Na^+$: 274.0443 $[M+Na]^+$; found: 274.0451.

4-Phenyl-6-(propylthio)-3,4-dihydro-1,3,5-triazine-2(1*H*)-thione (6ac)

White solid (85 mg, 32%); m.p. 109.6-110.2 ºC; ^{1}H NMR (300 MHz, DMSO-*d6*) δ = 11.12 (s, 1H, NH), 9.72 (s, 1H, NH), 7.42-7.29 (m, 5H, Ar-H), 5.79 (d, J = 1.8 Hz, 1H, CH), 2.97-2.80 (m, 2H, CH2), 1.56 (m, J = 7.2 Hz, 2H, CH2CH3), 0.88 (t, J = 7.2 Hz, 3H, CH2CH3); ^{13}C NMR (75 MHz, DMSO-*d6*) δ = 174.6, 151.0, 142.1, 128.9, 128.5, 126.5, 70.4, 31.5, 22.5, 13.6; IR ν_{max} (neat): 3188 (br), 2965 (w), 2871 (w), 1651 (s), 1564 (s), 1455 (s), 1402 (w), 1183 (s), 1143 (vs), 760 (m), 695 (s); HRMS (ESI): *m*/*z* calcd. for $C_{12}H_{15}N_3S_2+H^+$: 266.0780 $[M+H]^+$; found: 266.0783.

6-(Butylthio)-4-phenyl-3,4-dihydro-1,3,5-triazine-2(1*H*)-thione (6ad)

White solid (112 mg, 40%); m.p. 87.1-88.0 ºC; ^{1}H NMR (300 MHz, DMSO-*d6*) δ = 11.13 (s, 1H, NH), 9.73 (s, 1H, NH), 7.42-7.28 (m, 5H, Ar-H), 5.79 (d, J = 2.1 Hz, 1H, CH), 2.99-2.81 (m, 2H, SCH2), 1.56-1.46 (m, 2H, CH2), 1.35-1.26 (m, 2H, CH2CH3), 0.81 (t, J = 7.2 Hz, 3H, CH3); ^{13}C NMR (75 MHz, DMSO-*d6*) δ = 174.6, 150.9, 142.1, 128.9, 128.5, 126.4, 70.4, 31.2, 29.3, 21.7, 12.9; IR ν_{max} (neat): 3126 (br), 2961 (w), 2928 (w), 2867 (w), 1642 (m), 1572 (s), 1470 (m), 1376 (w), 1190 (s), 1147 (vs), 827 (m), 760 (w), 692 (vs), 651 (m); HRMS (ESI): *m*/*z* calcd. for $C_{13}H_{17}N_3S_2+H^+$: 280.0936 $[M+H]^+$; found: 280.0937.

6-Phenyl-1,3,5-triazinane-2,4-dithione (7)

Benzaldehyde (1.0 mmol) was dissolved in DMF (1.0 mL) in a reaction vial (10 mL). Thiourea (1.0 mmol) was added. The mixture was stirred at 80 °C for 5 h. The solvent was removed under reduced pressure. The residue was purified by chromatography on silica gel (ethyl acetate/petroleum ether 1:10 to 1:2) to give **7**. White solid (56 mg, 25%); m.p. 241.2-242.8 ºC (lit. [1] 242–244 ºC); ^{1}H NMR (300 MHz, DMSO-*d6*) δ = 11.46 (s, 1H, NH), 10.44 (s, 2H, 2CHNH), 7.47-7.29 (m, 5H, Ar-H), 5.60 (s, 1H, CH); ^{13}C NMR (75 MHz, DMSO-*d6*) δ = 173.8, 140.0, 129.4, 129.2, 126.2, 65.1; IR ν_{max} (neat): 3087 (br), 2945 (br), 1574 (vs), 1456 (m), 1434 (m), 1168 (vs), 760 (s), 690 (vs); HRMS (ESI): m/z calcd. for $C_{18}H_{18}N_6S_4-H^-$: 445.0392 $[2M-H]^-$; found: 445.0396. NMR spectral data is consistent with the literature [1].

^1H NMR, ^{13}C NMR and ^{19}F NMR spectra of compounds in DMSO-d_6.

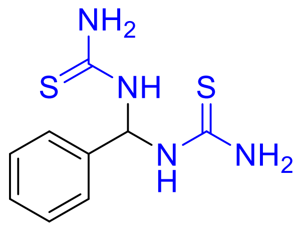

1,1'-(Phenylmethylene)bis(thiourea) (4)

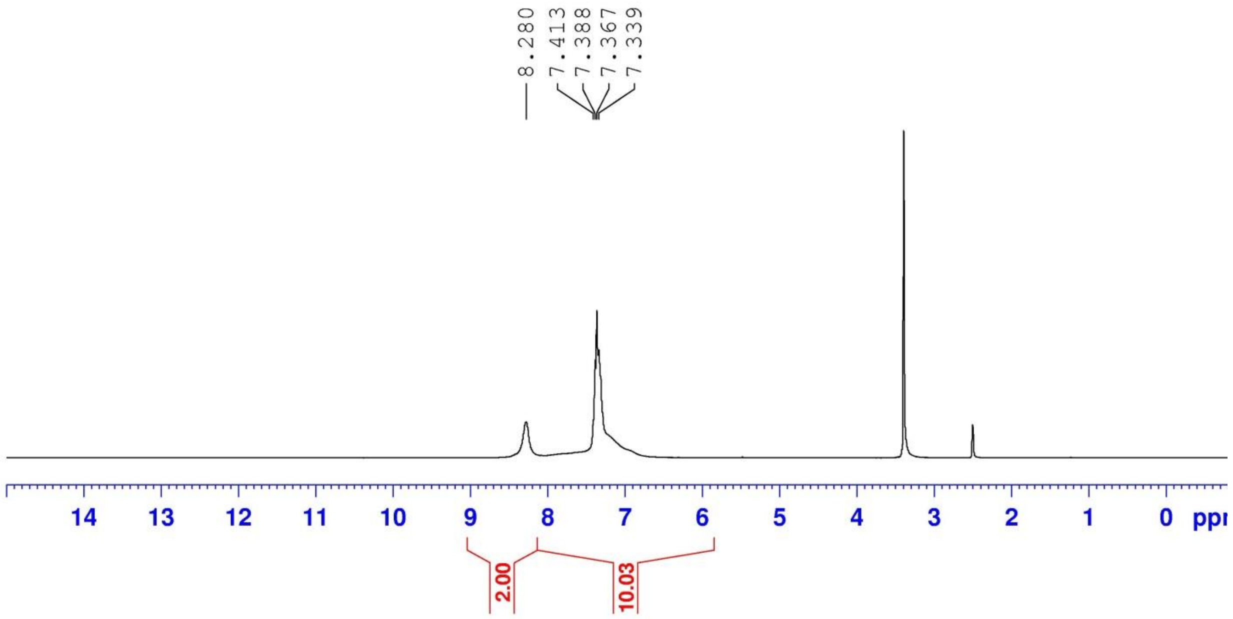

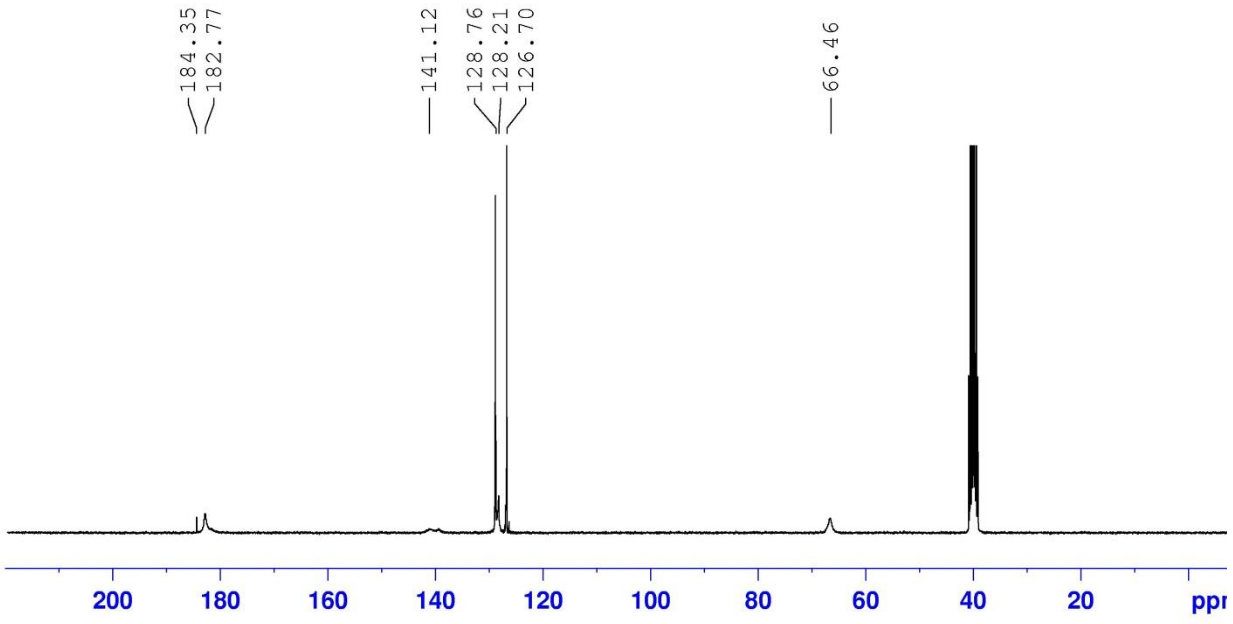

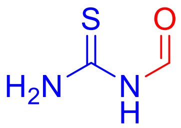

***N*-Carbamothioylformamide (5)**

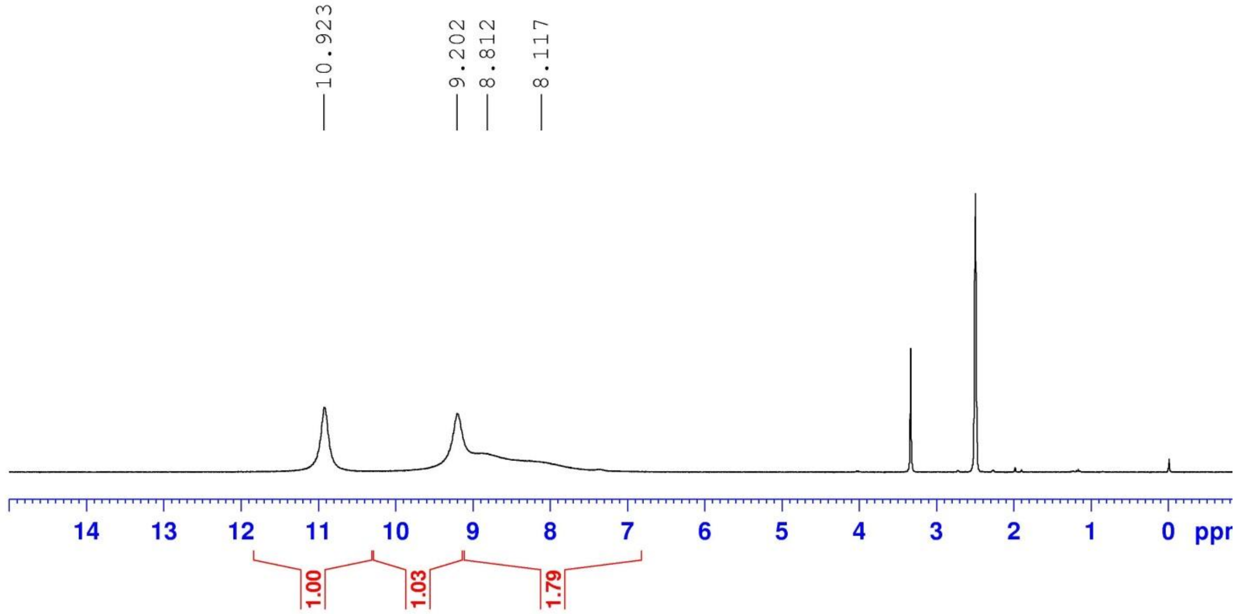

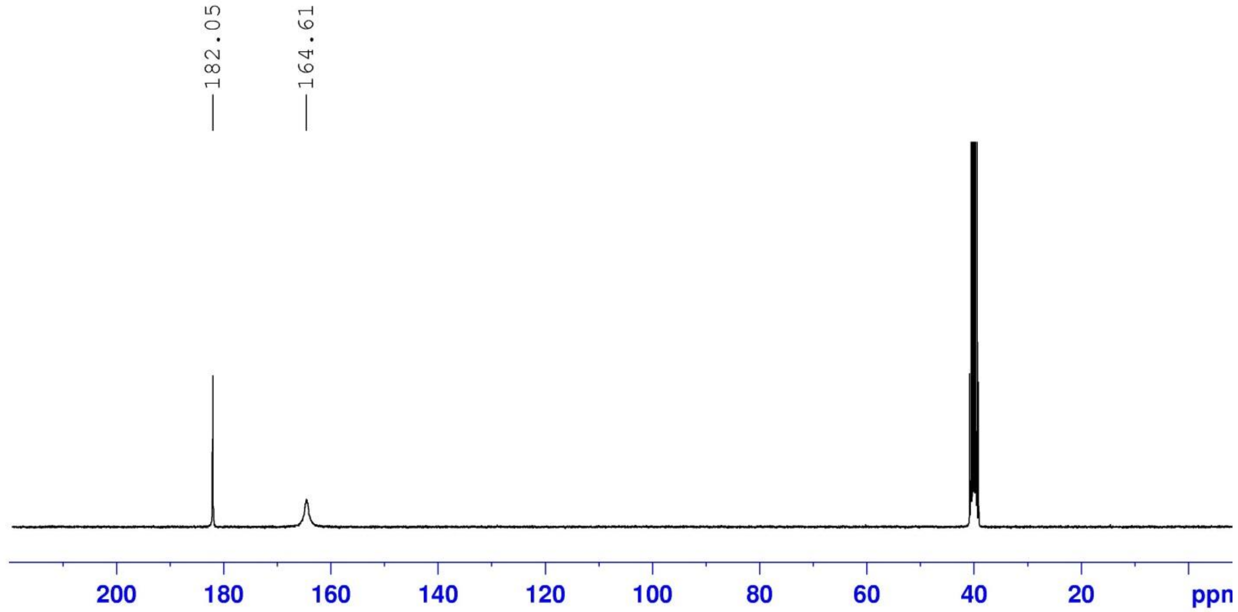

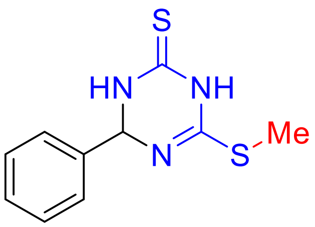

6-(Methylthio)-4-phenyl-3,4-dihydro-1,3,5-triazine-2(1*H*)-thione (6aa)

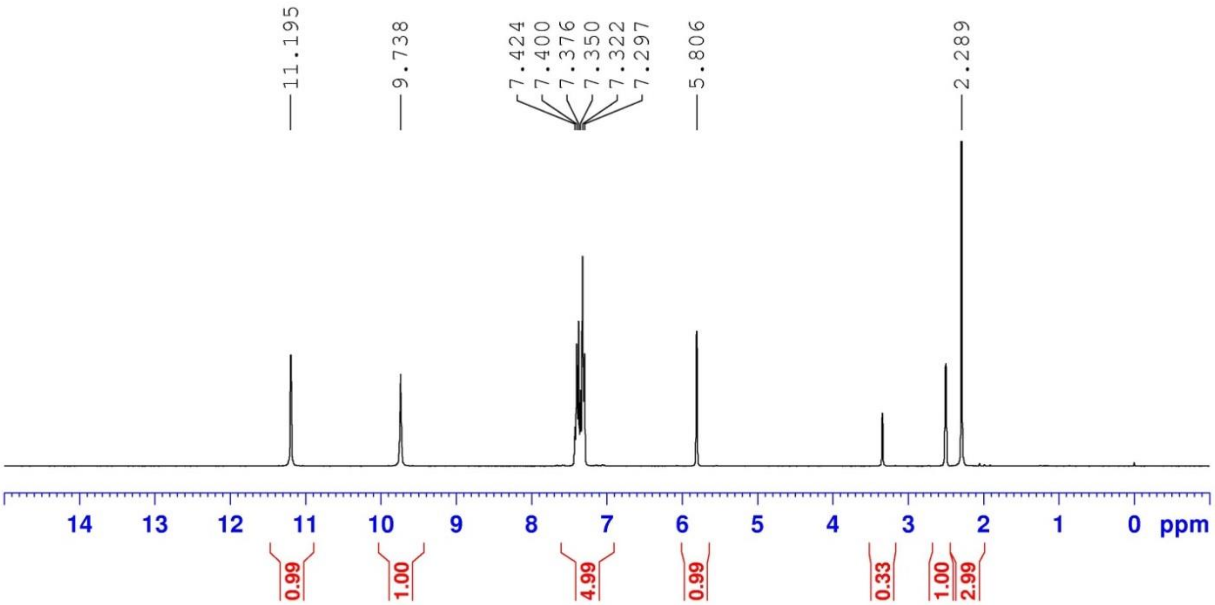

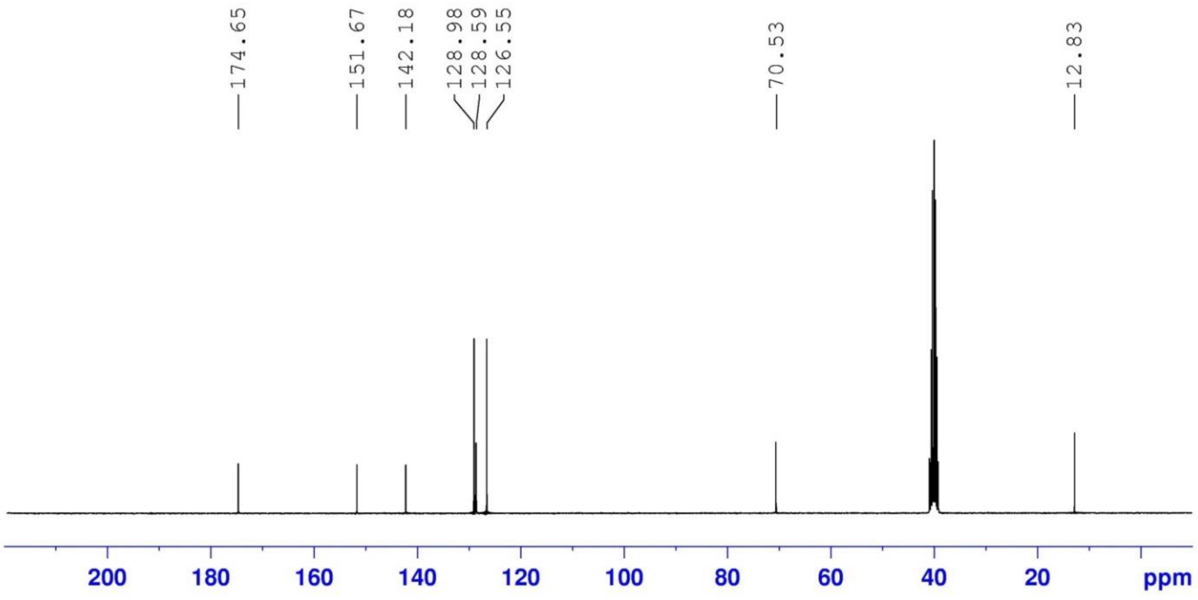

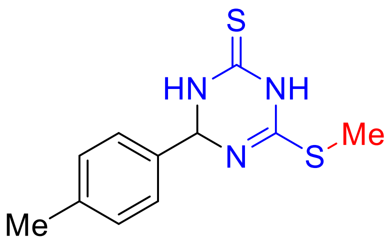

6-(Methylthio)-4-(*p*-tolyl)-3,4-dihydro-1,3,5-triazine-2(1*H*)-thione (6ba)

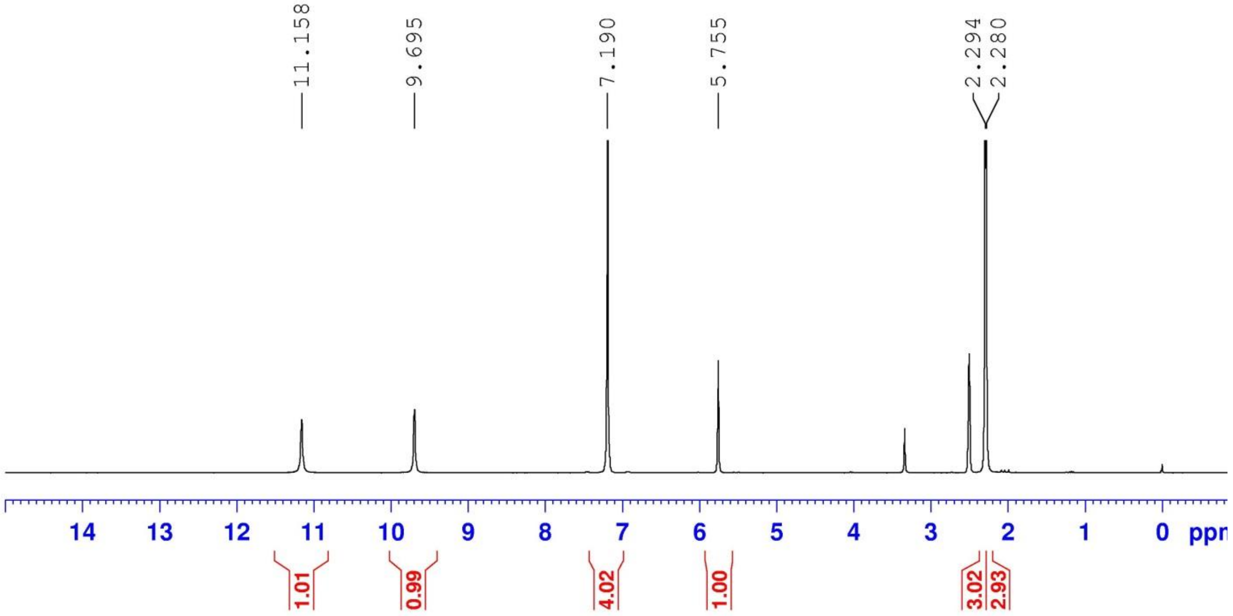

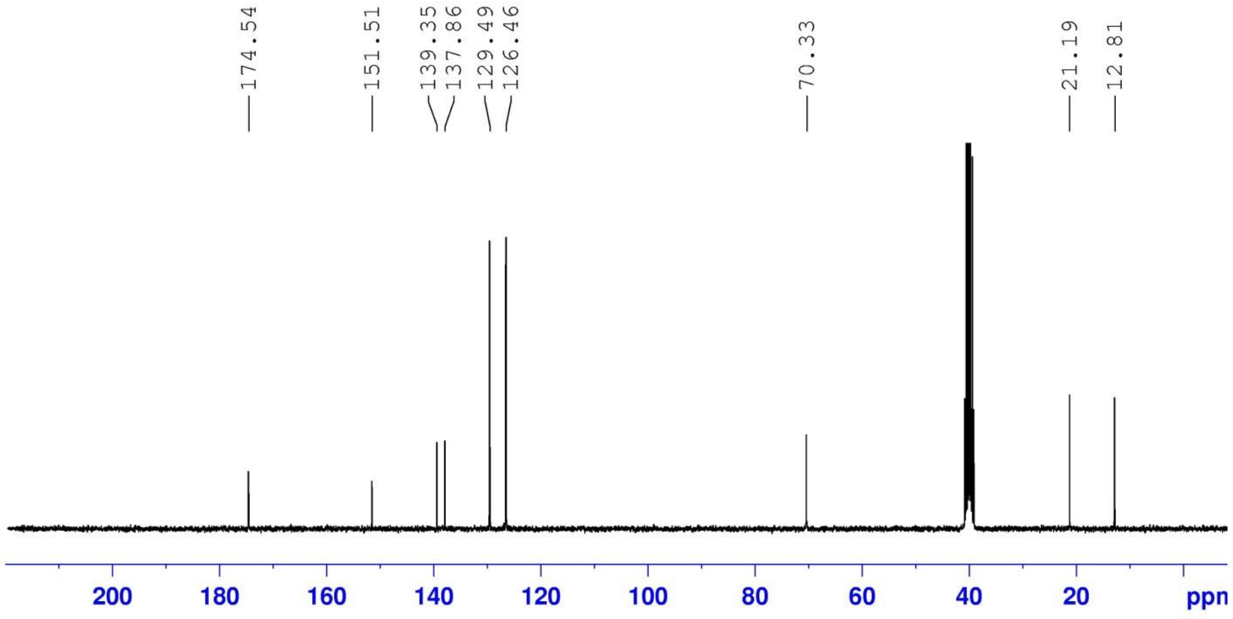

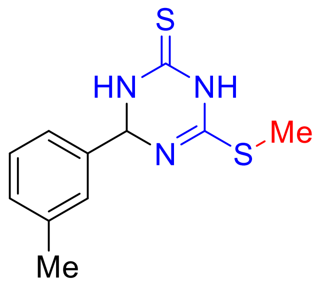

6-(Methylthio)-4-(*m*-tolyl)-3,4-dihydro-1,3,5-triazine-2(1*H*)-thione (6ca)

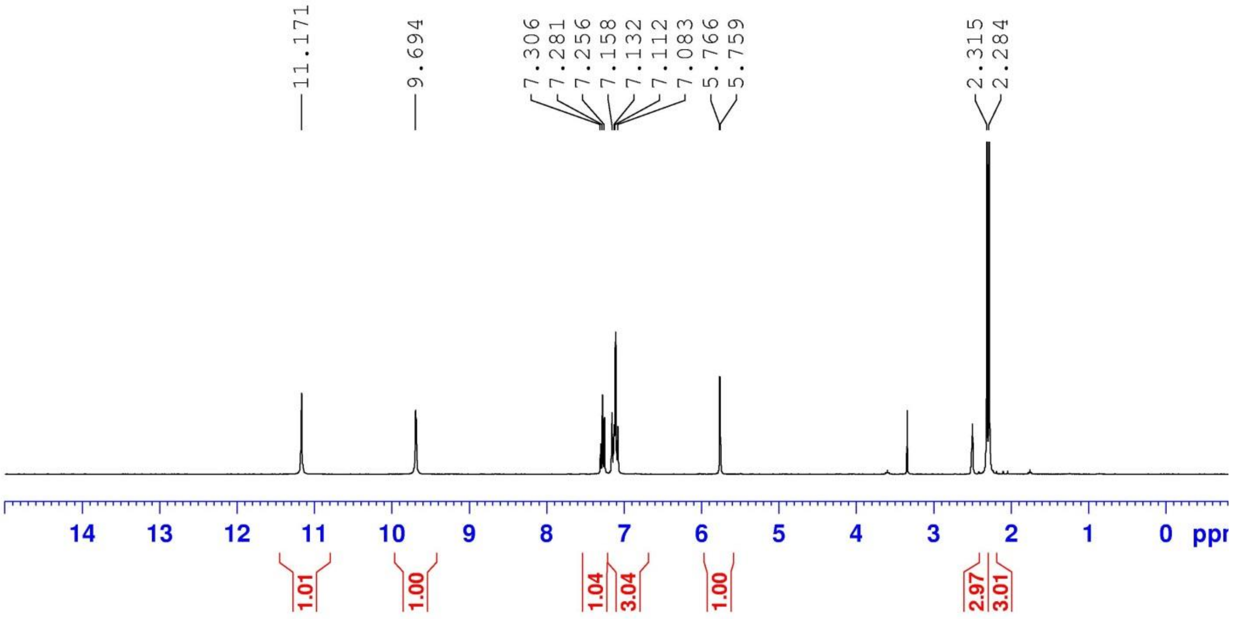

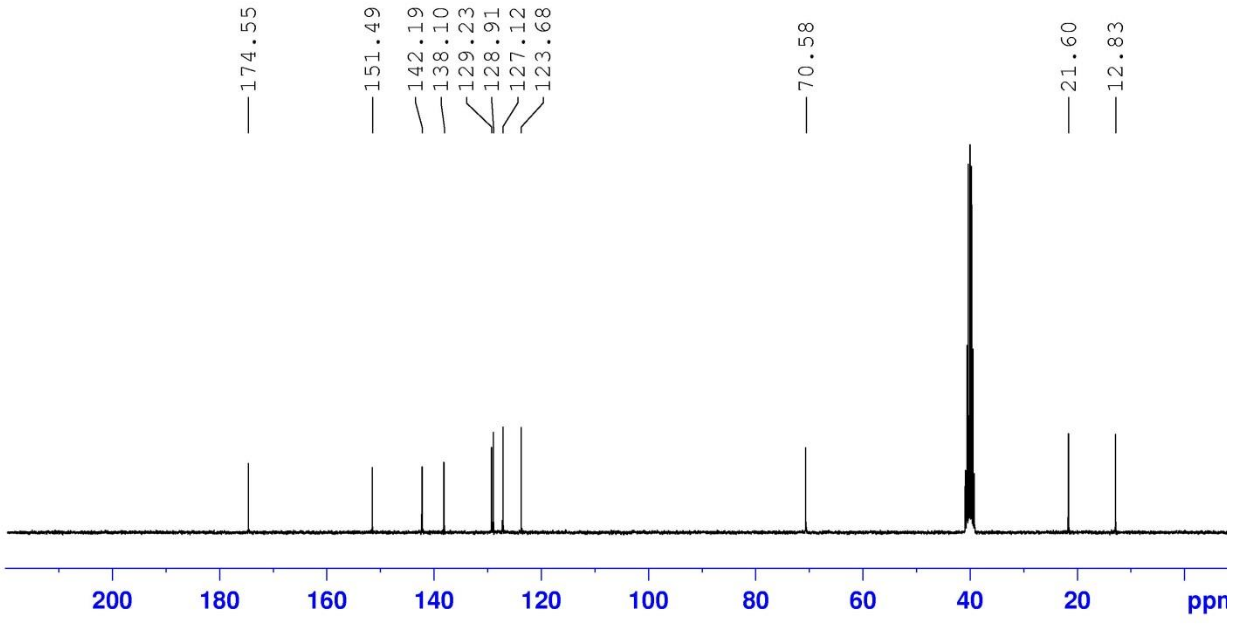

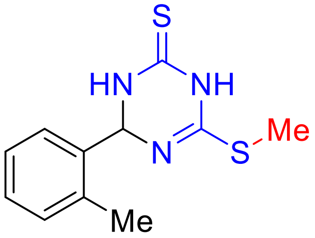

6-(Methylthio)-4-(*o*-tolyl)-3,4-dihydro-1,3,5-triazine-2(1*H*)-thione (6da)

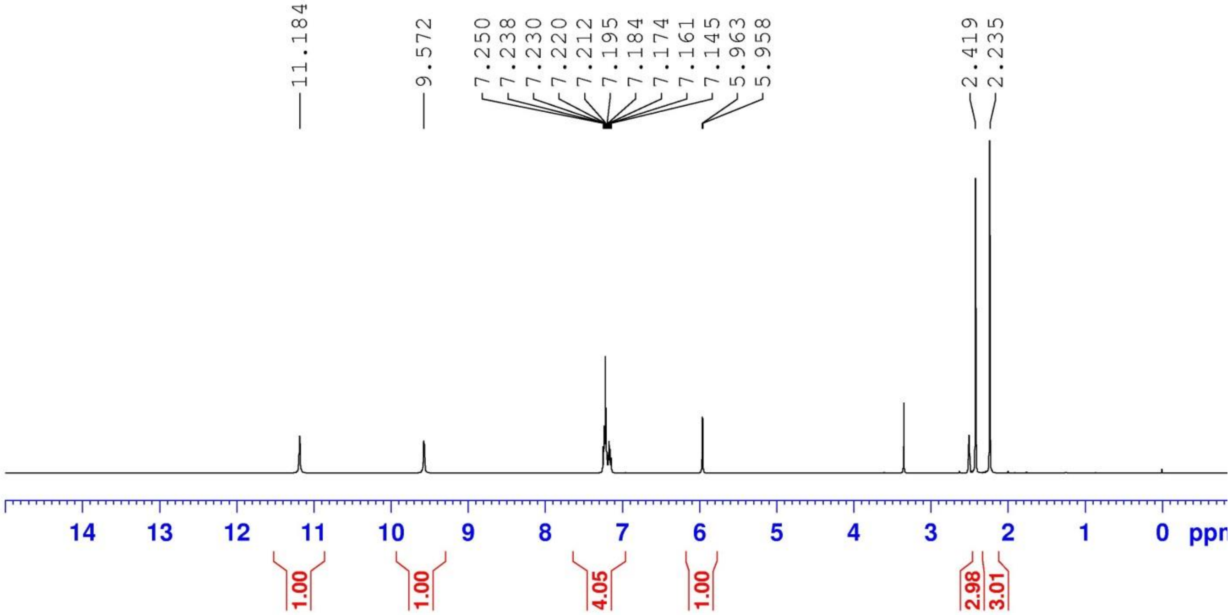

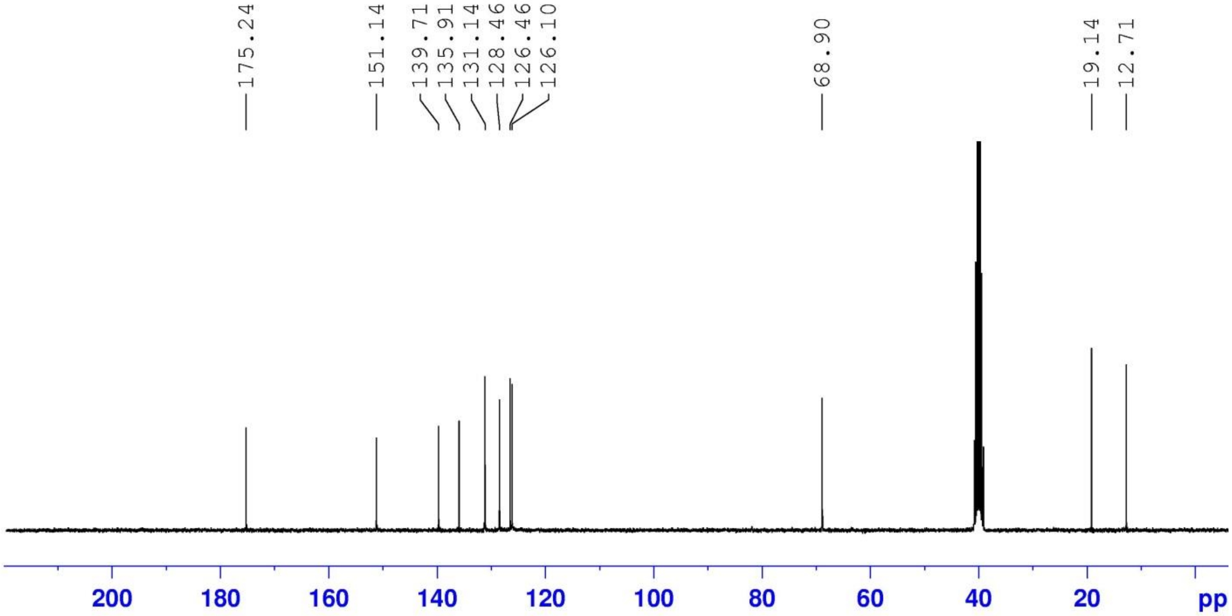

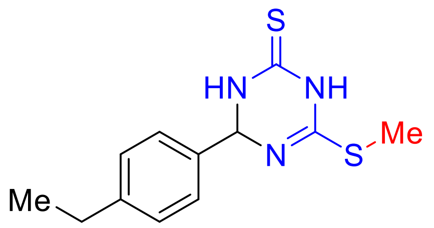

4-(4-Ethylphenyl)-6-(methylthio)-3,4-dihydro-1,3,5-triazine-2(1*H*)-thione (6ea)

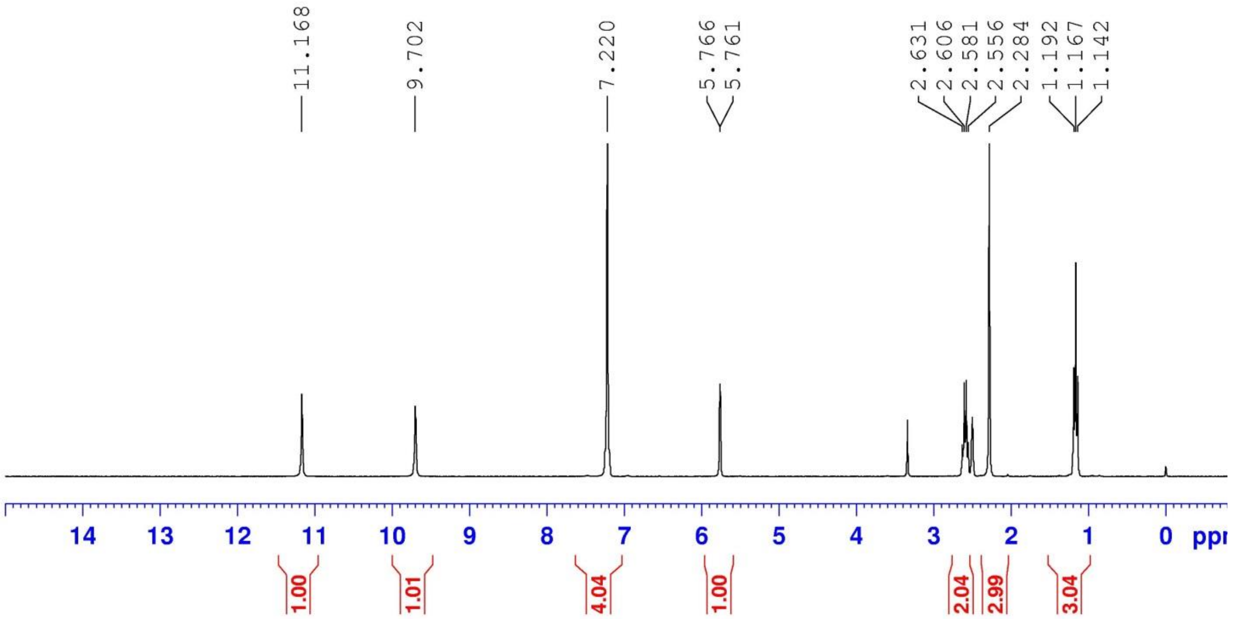

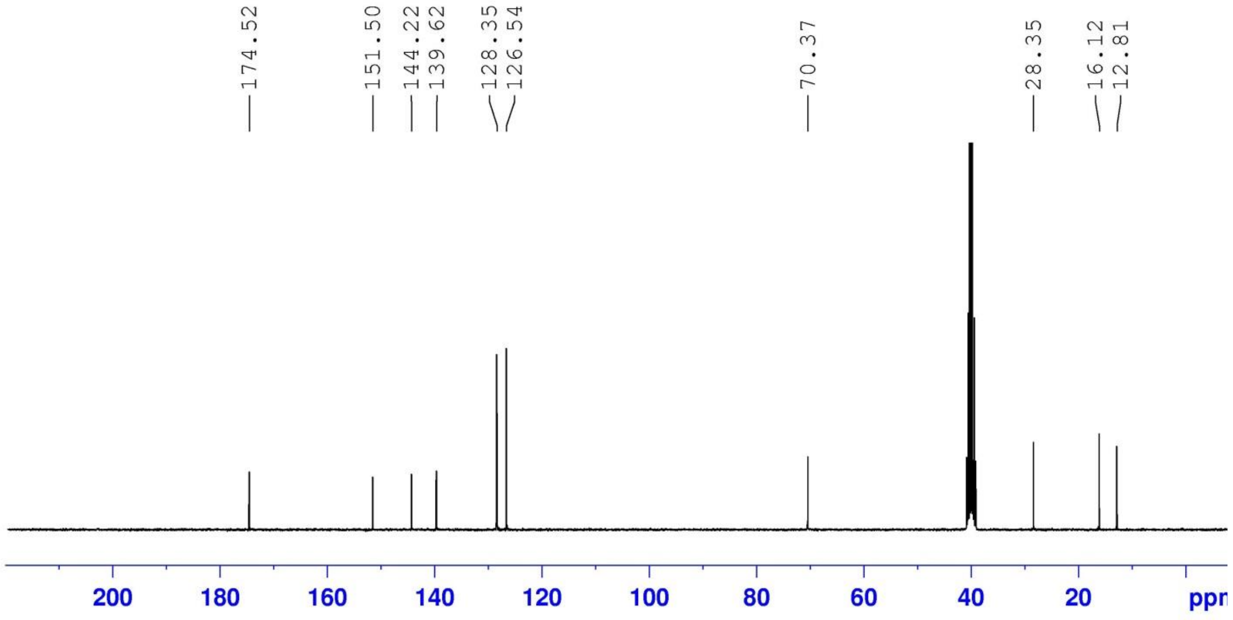

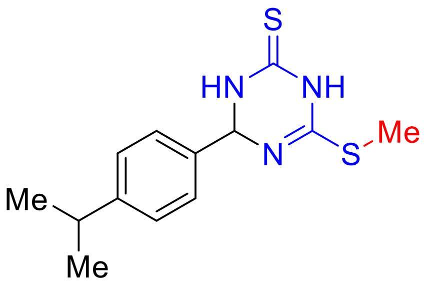

4-(4-Isopropylphenyl)-6-(methylthio)-3,4-dihydro-1,3,5-triazine-2(1*H*)-thione (6fa)

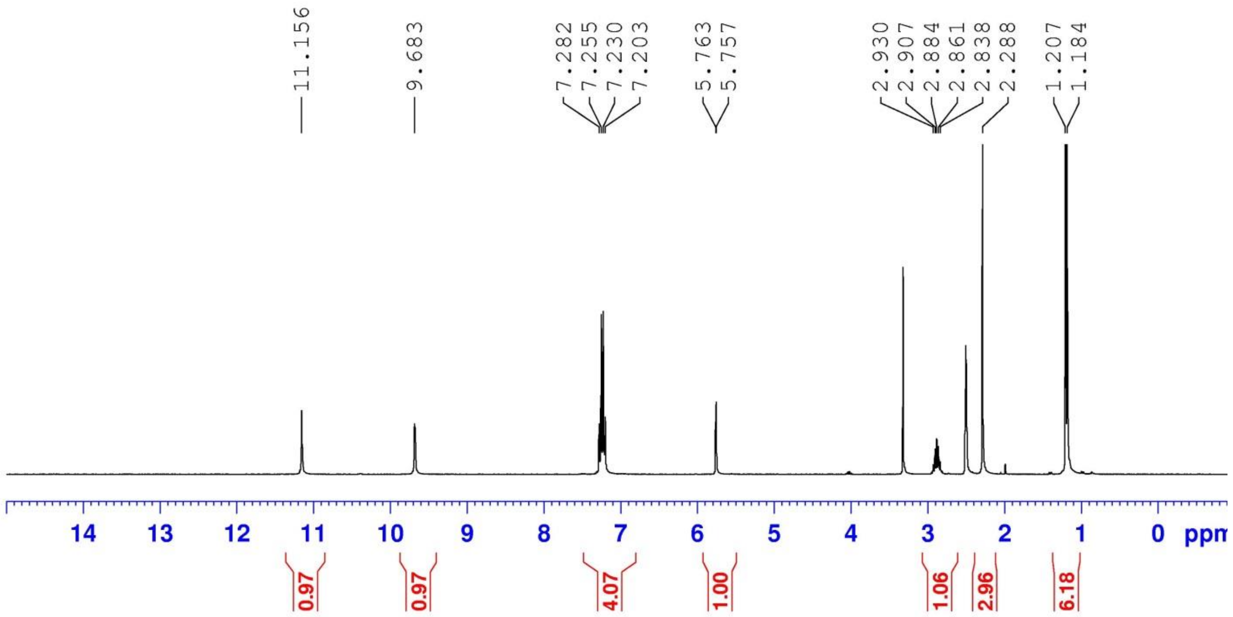

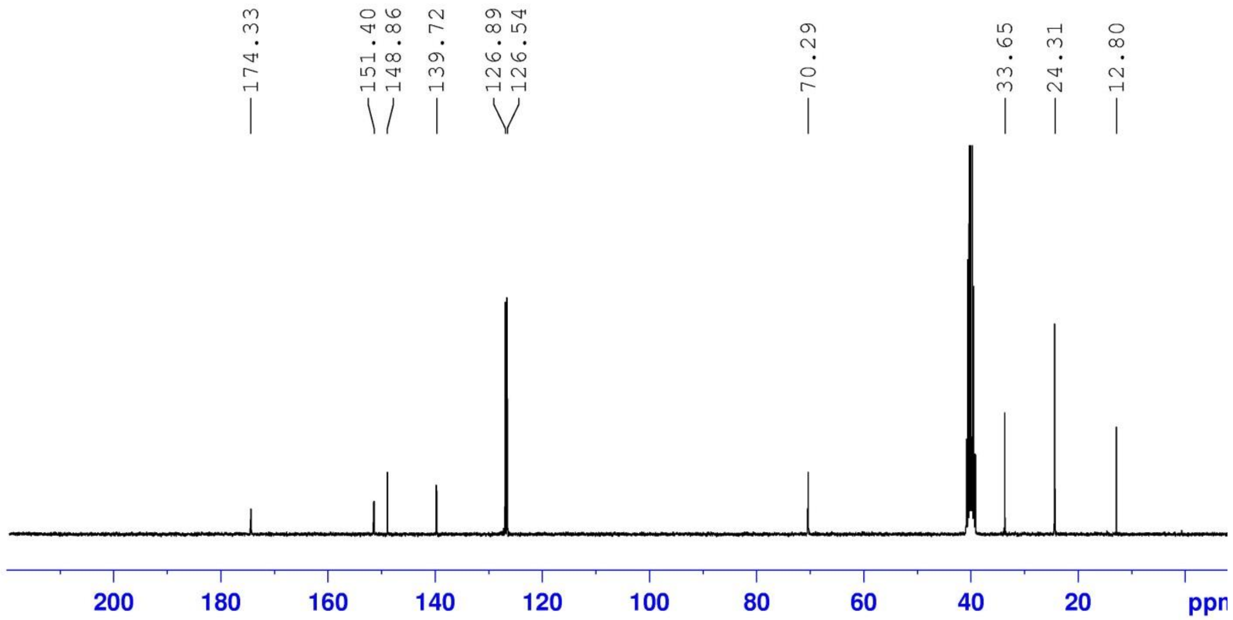

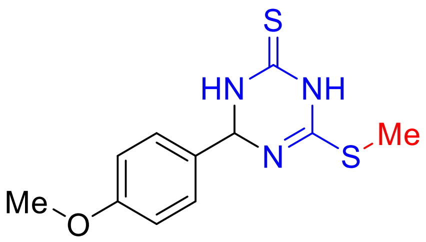

4-(4-Methoxyphenyl)-6-(methylthio)-3,4-dihydro-1,3,5-triazine-2(1*H*)-thione (6ga)

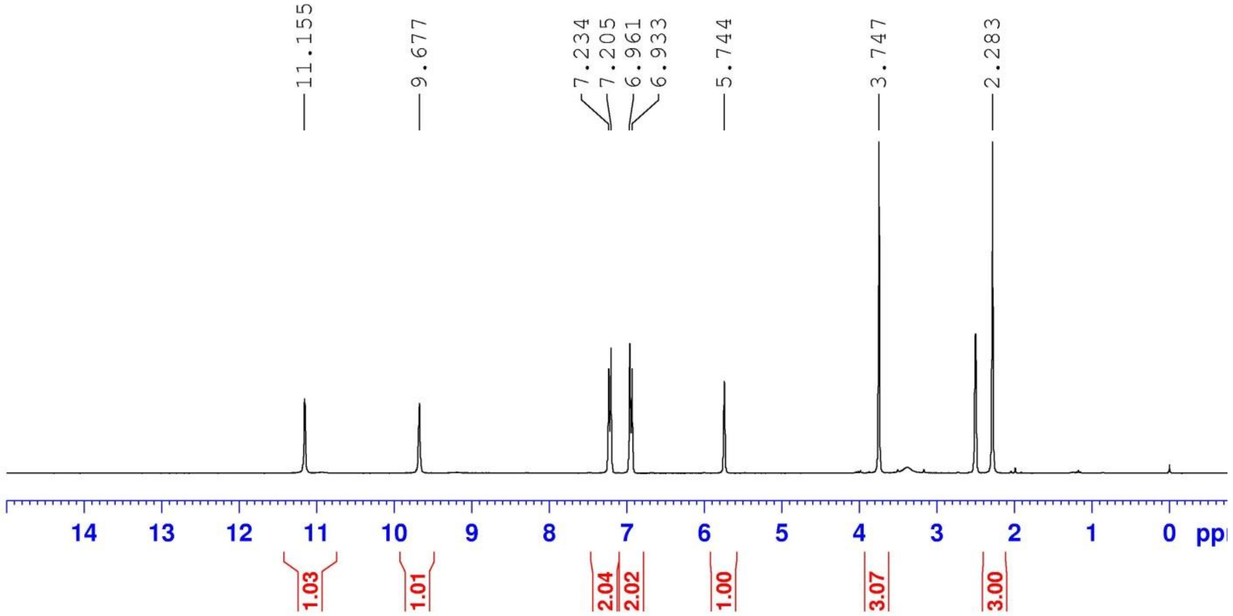

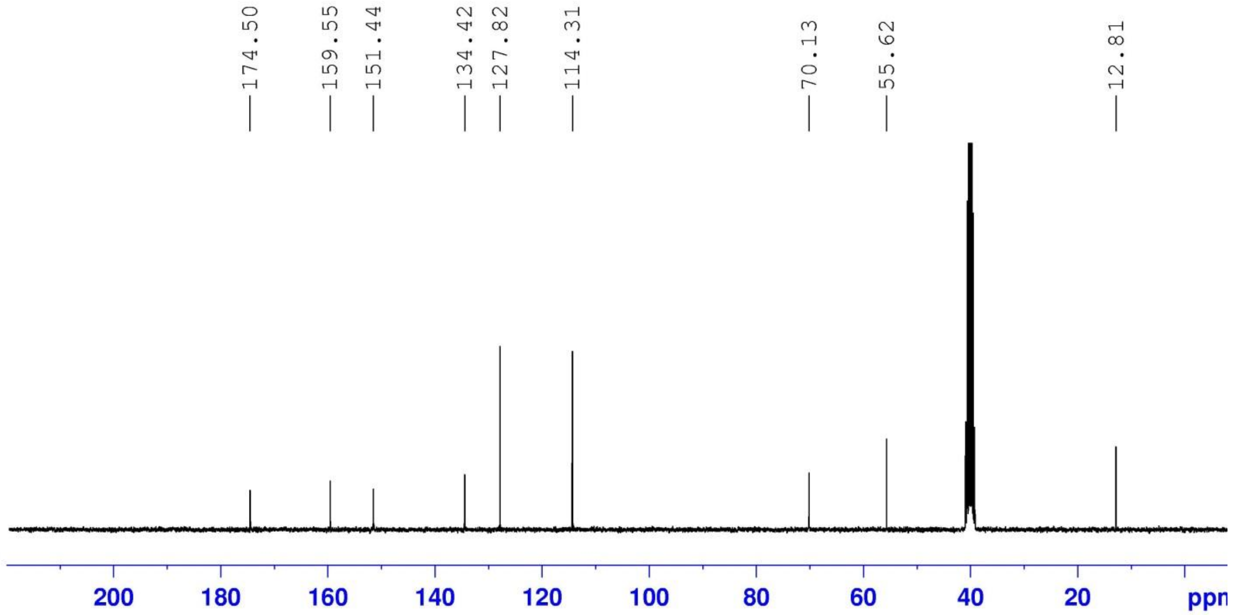

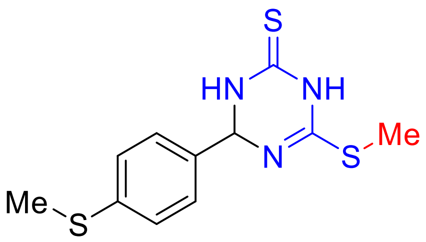

6-(Methylthio)-4-(4-(methylthio)phenyl)-3,4-dihydro-1,3,5-triazine-2(1*H*)-thione (6ha)

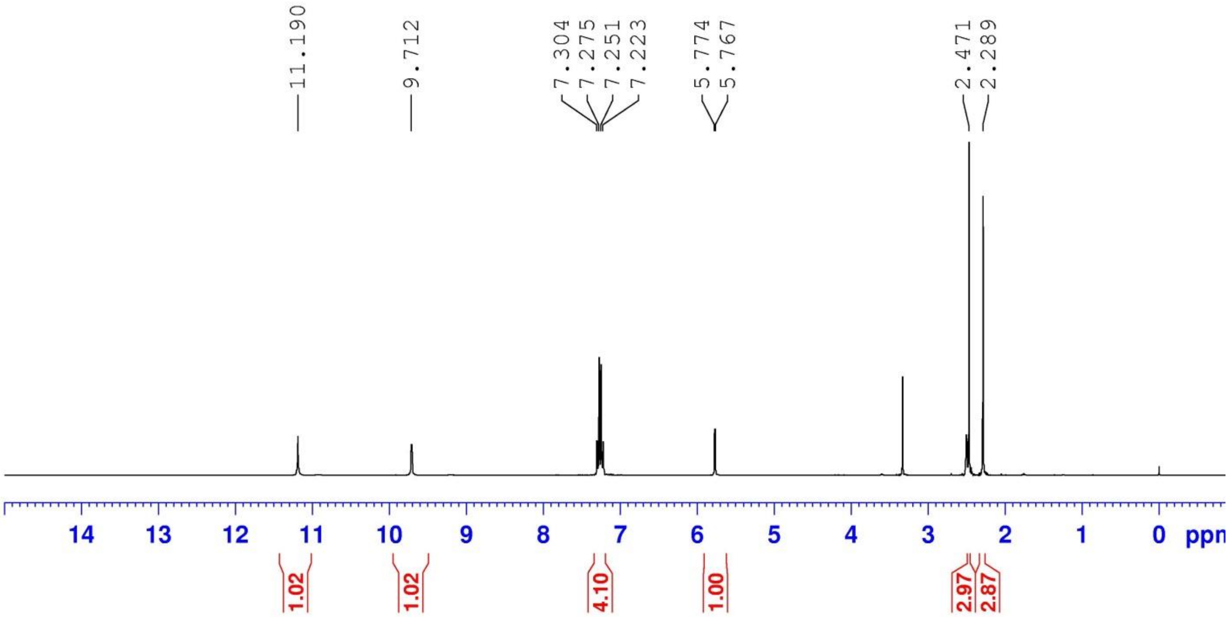

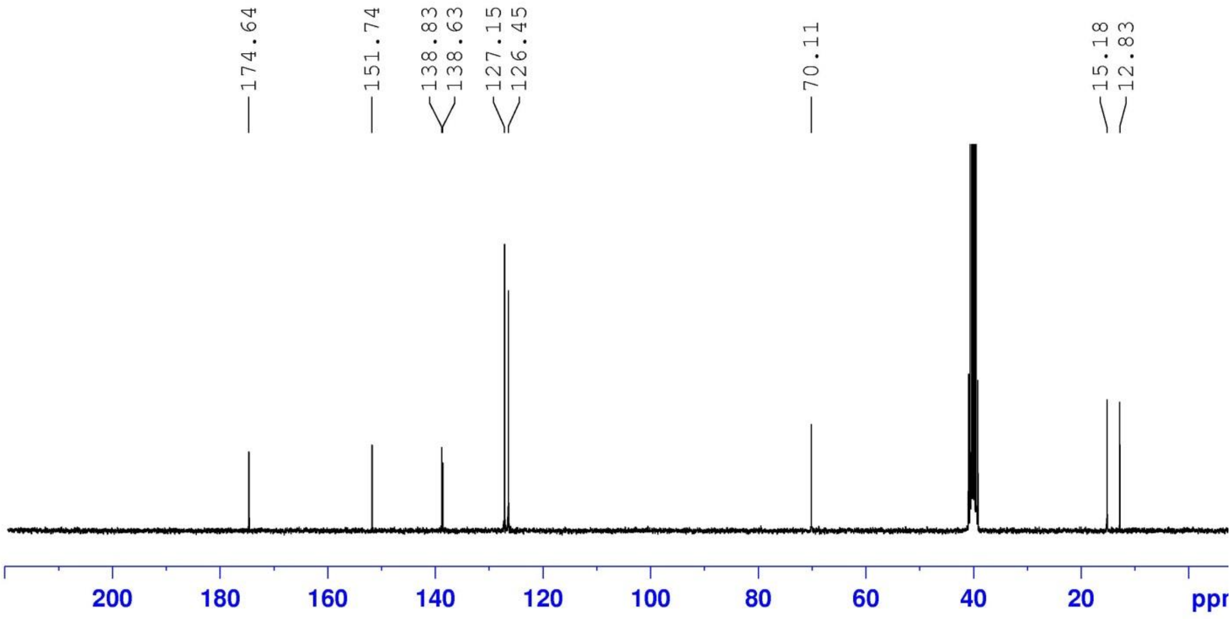

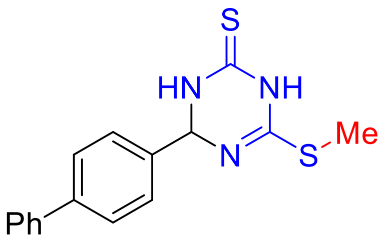

4-([1,1'-Biphenyl]-4-yl)-6-(methylthio)-3,4-dihydro-1,3,5-triazine-2(1*H*)-thione (6ia)

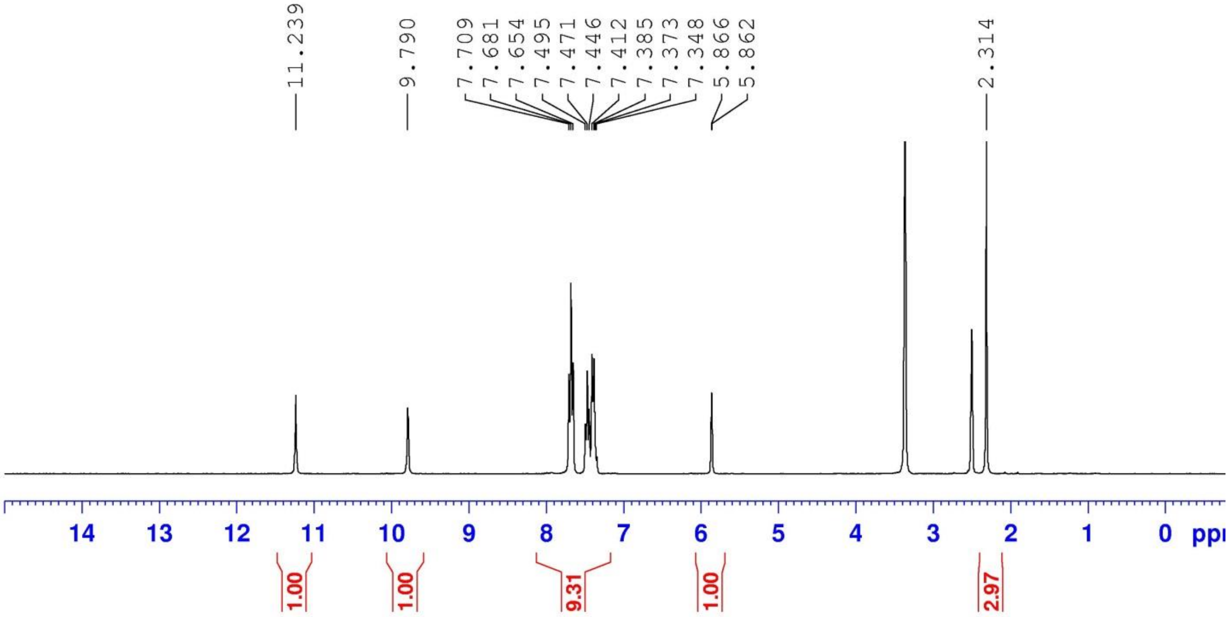

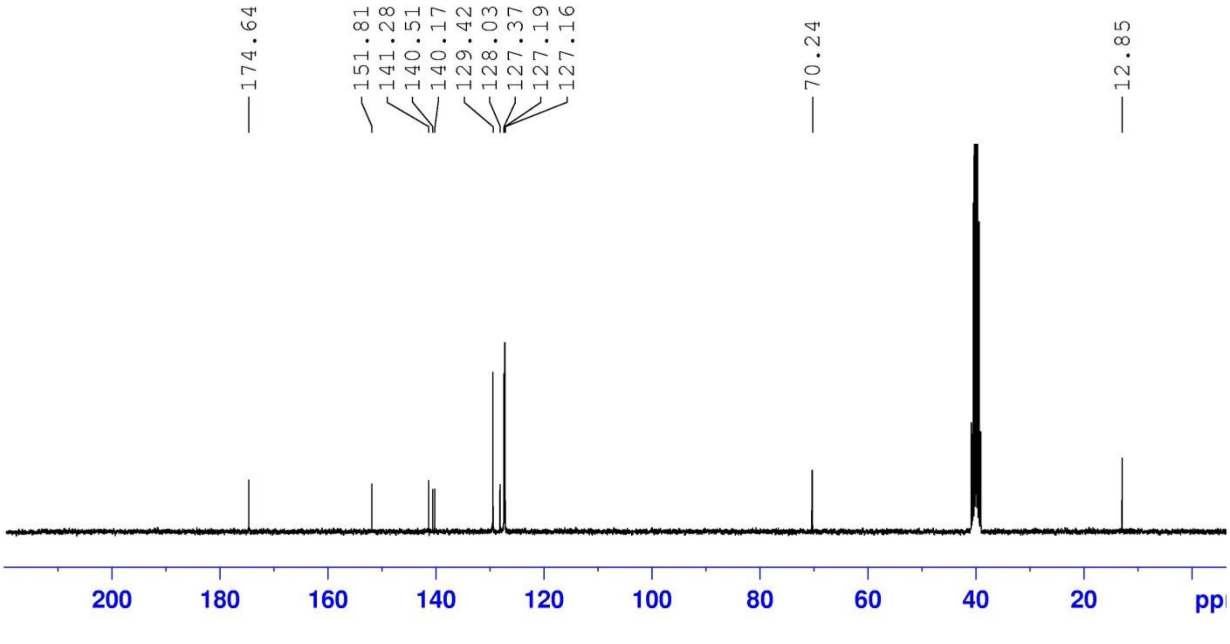

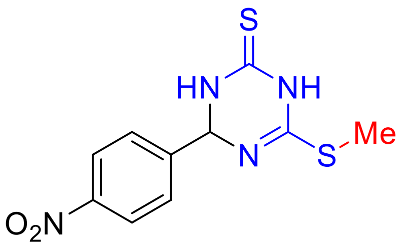

6-(Methylthio)-4-(4-nitrophenyl)-3,4-dihydro-1,3,5-triazine-2(1*H*)-thione (6ja)

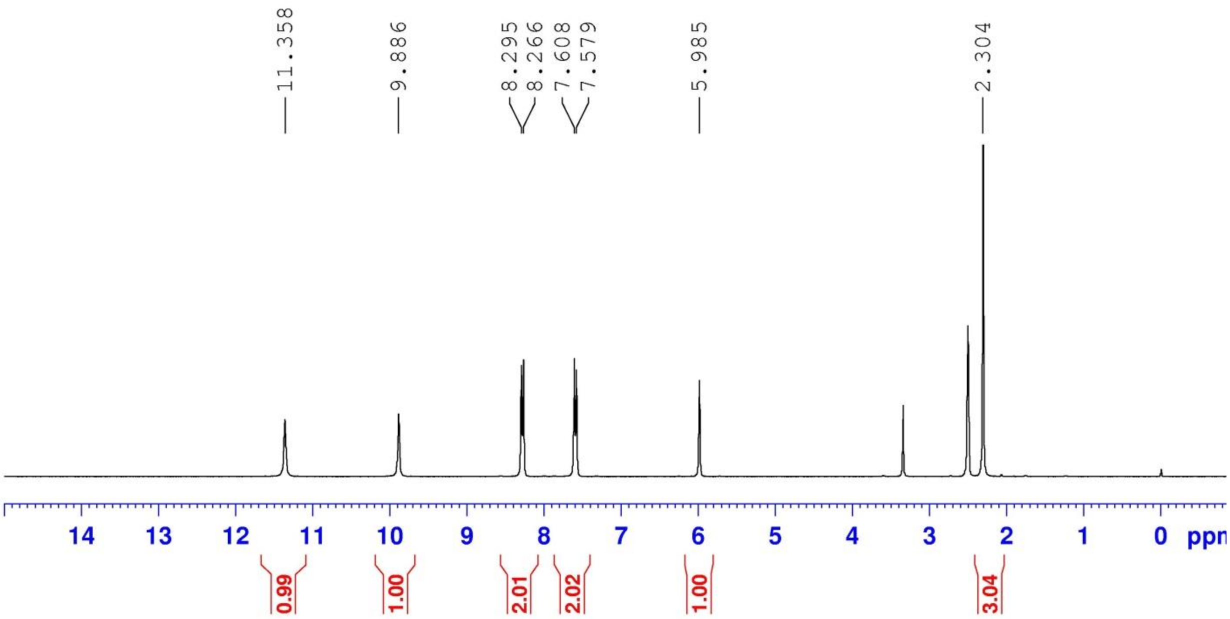

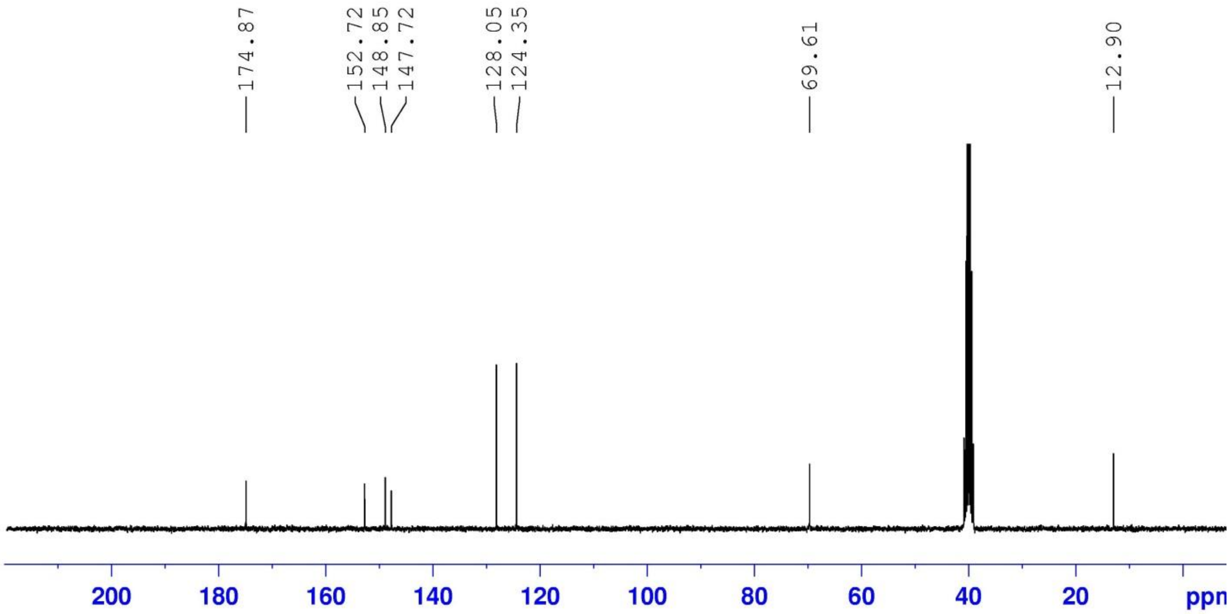

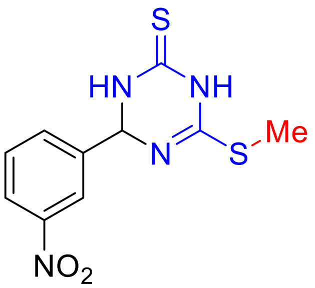

6-(Methylthio)-4-(3-nitrophenyl)-3,4-dihydro-1,3,5-triazine-2(1*H*)-thione (6ka)

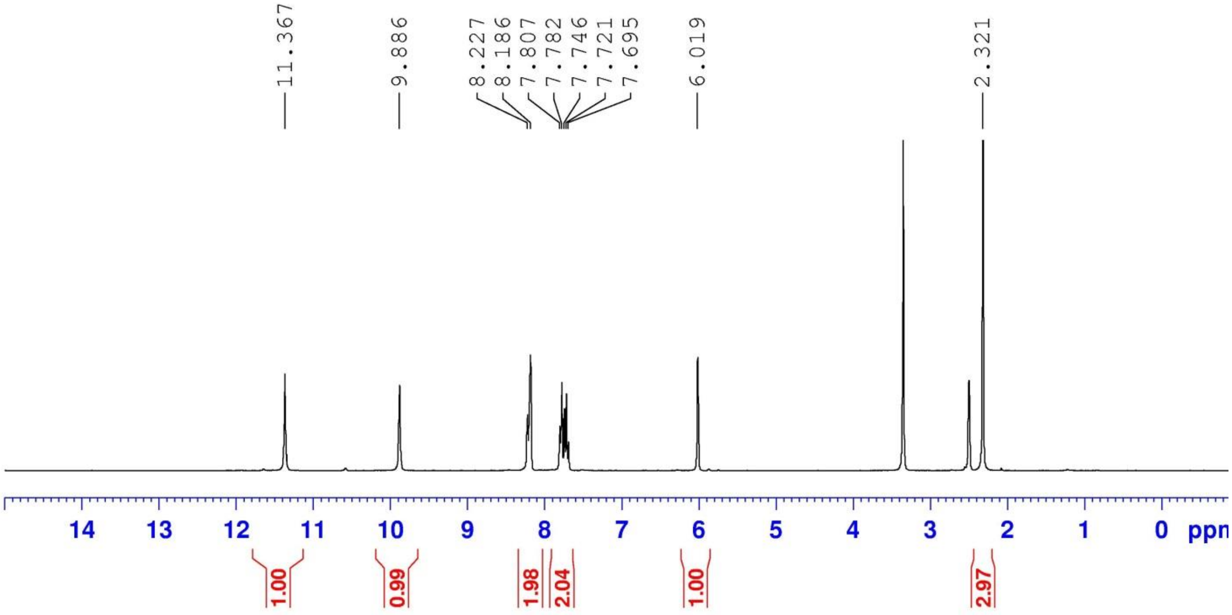

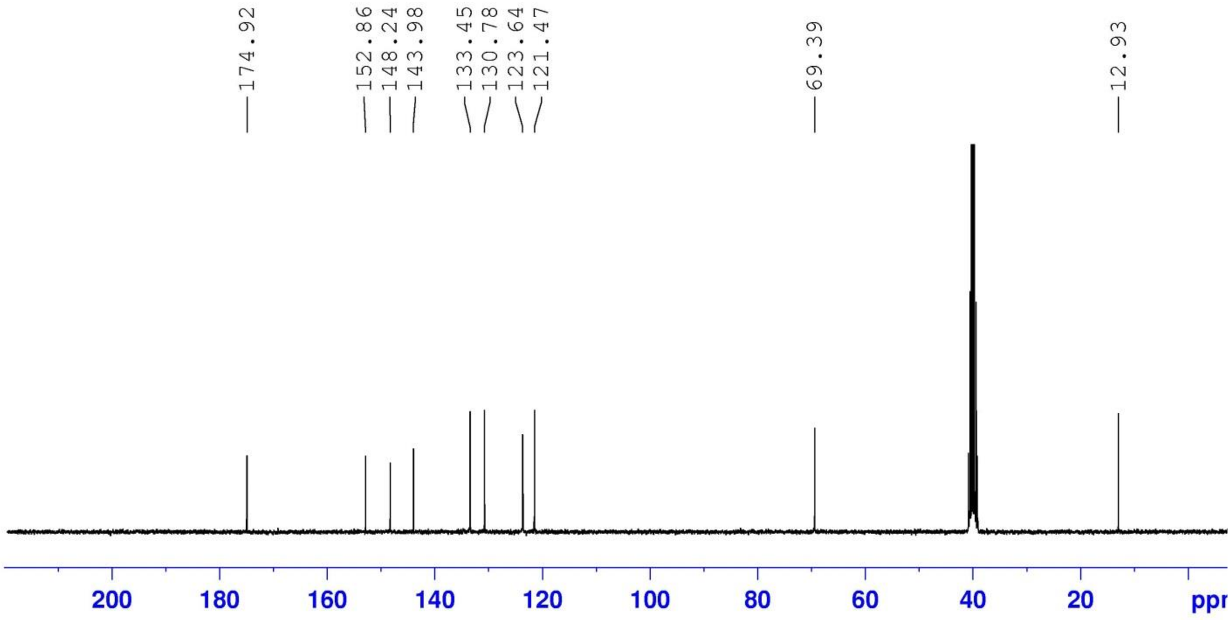

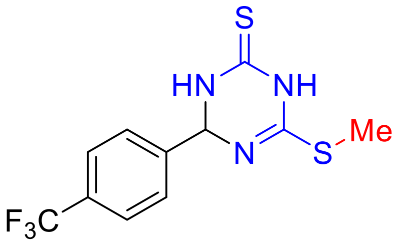

6-(Methylthio)-4-(4-(trifluoromethyl)phenyl)-3,4-dihydro-1,3,5-triazine-2(1*H*)-thione (6la)

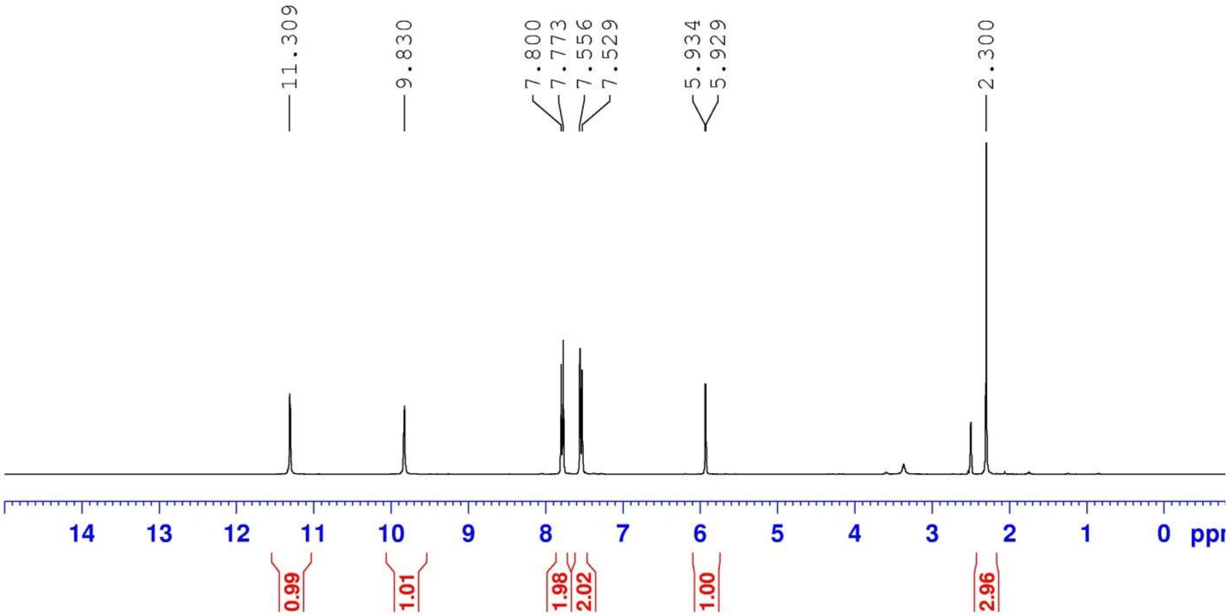

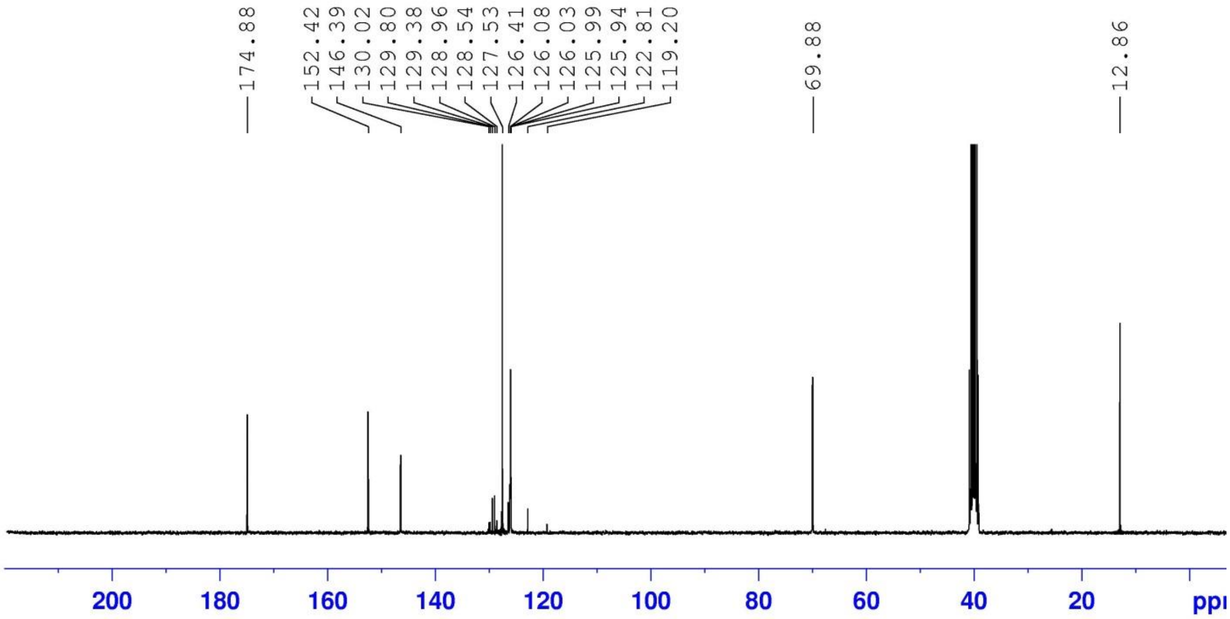
174.88
152.42
146.39
130.02
129.80
129.38
128.96
128.54
127.53
126.41
126.08
126.03
125.99
125.94
122.81
119.20
69.88
12.86
200 180 160 140 120 100 80 60 40 20 ppm

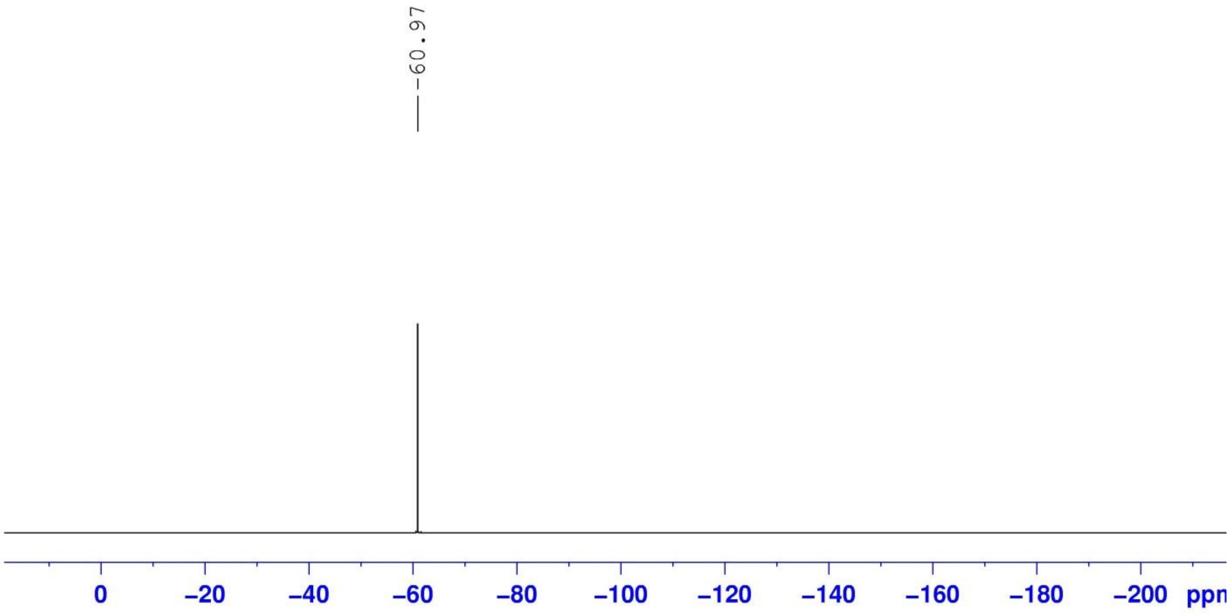
-60.97
0 −20 −40 −60 −80 −100 −120 −140 −160 −180 −200 ppm

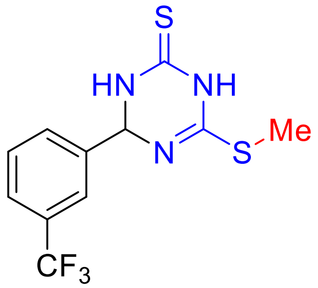

6-(Methylthio)-4-(3-(trifluoromethyl)phenyl)-3,4-dihydro-1,3,5-triazine-2(1*H*)-thione (6ma)

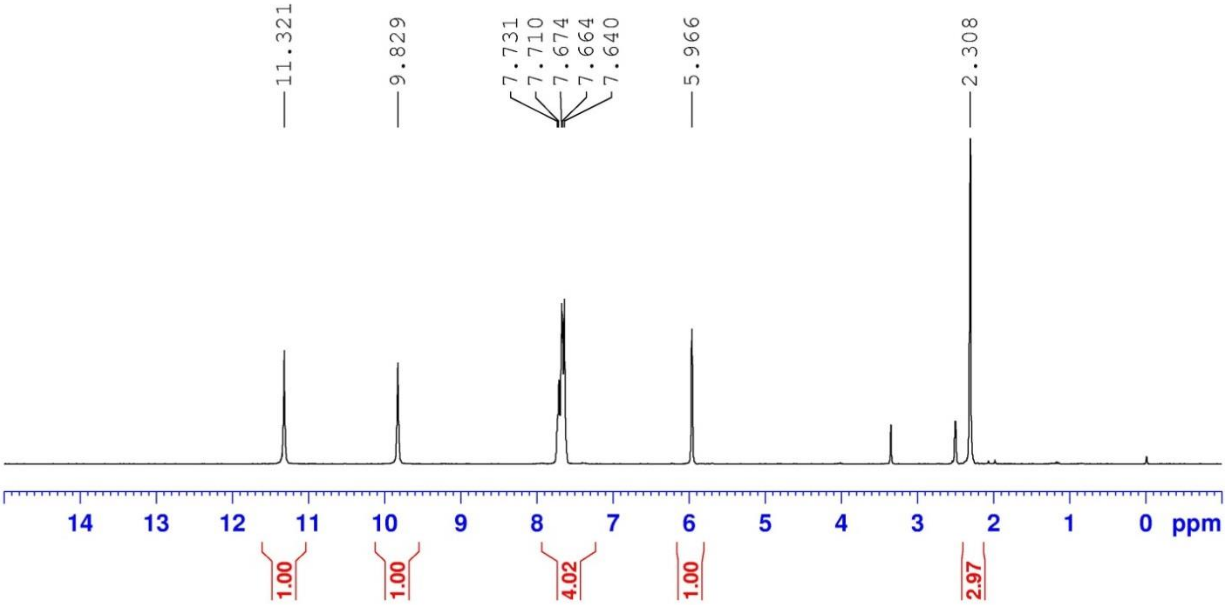

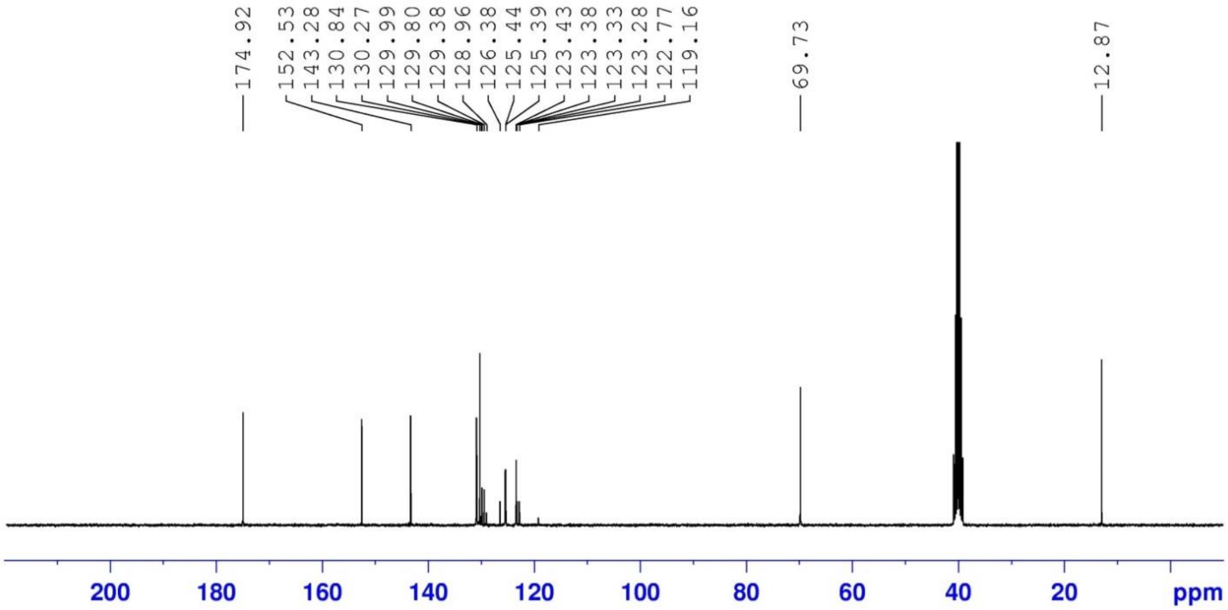
174.92
152.53
143.28
130.84
130.27
129.99
129.80
129.38
128.96
126.38
125.44
125.39
123.43
123.38
123.33
123.28
122.77
119.16
69.73
12.87
200 180 160 140 120 100 80 60 40 20 ppm

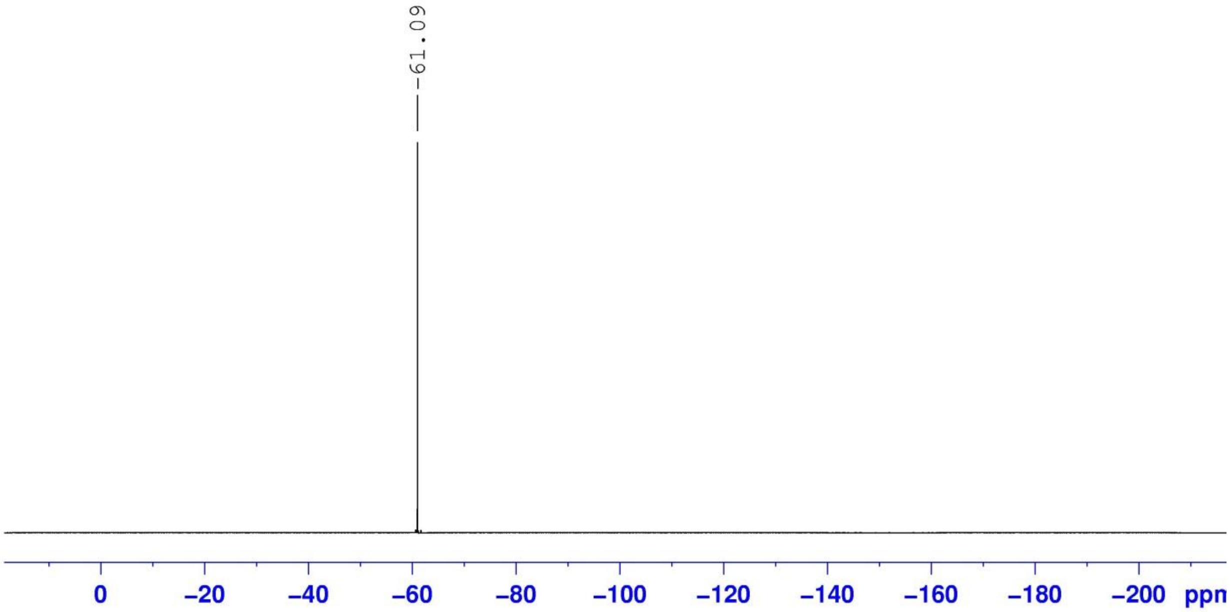
-61.09
0 −20 −40 −60 −80 −100 −120 −140 −160 −180 −200 ppm

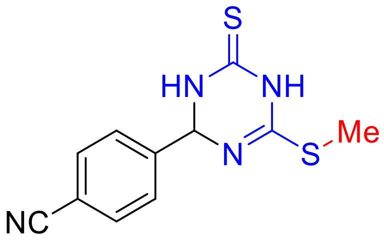

4-(4-(Methylthio)-6-thioxo-1,2,5,6-tetrahydro-1,3,5-triazin-2-yl)benzonitrile (6na)

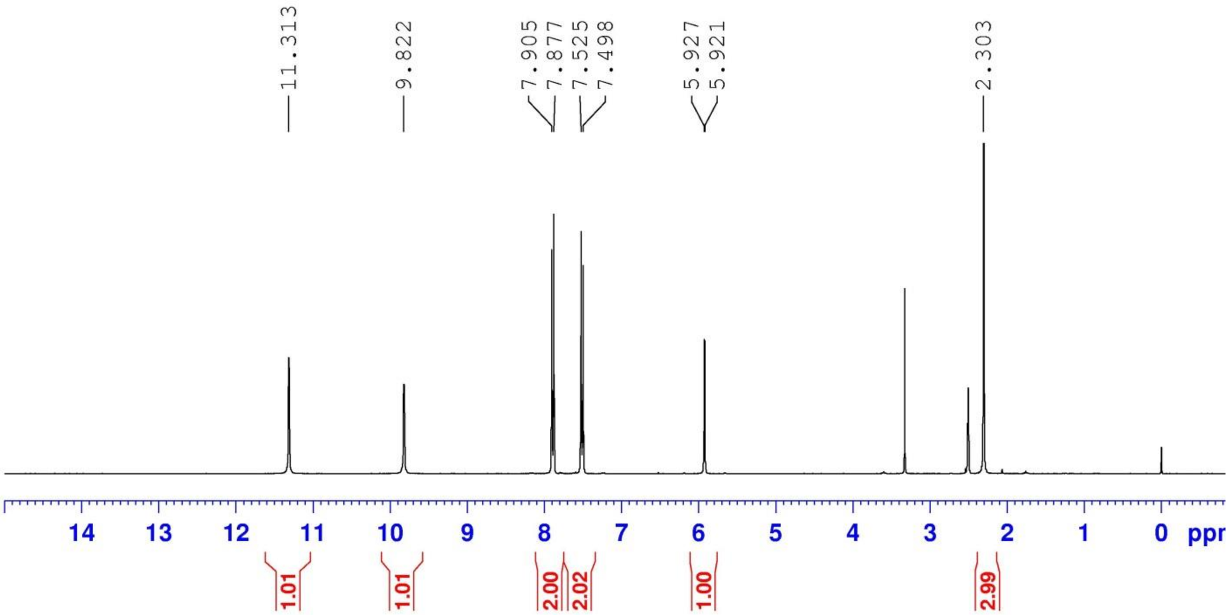

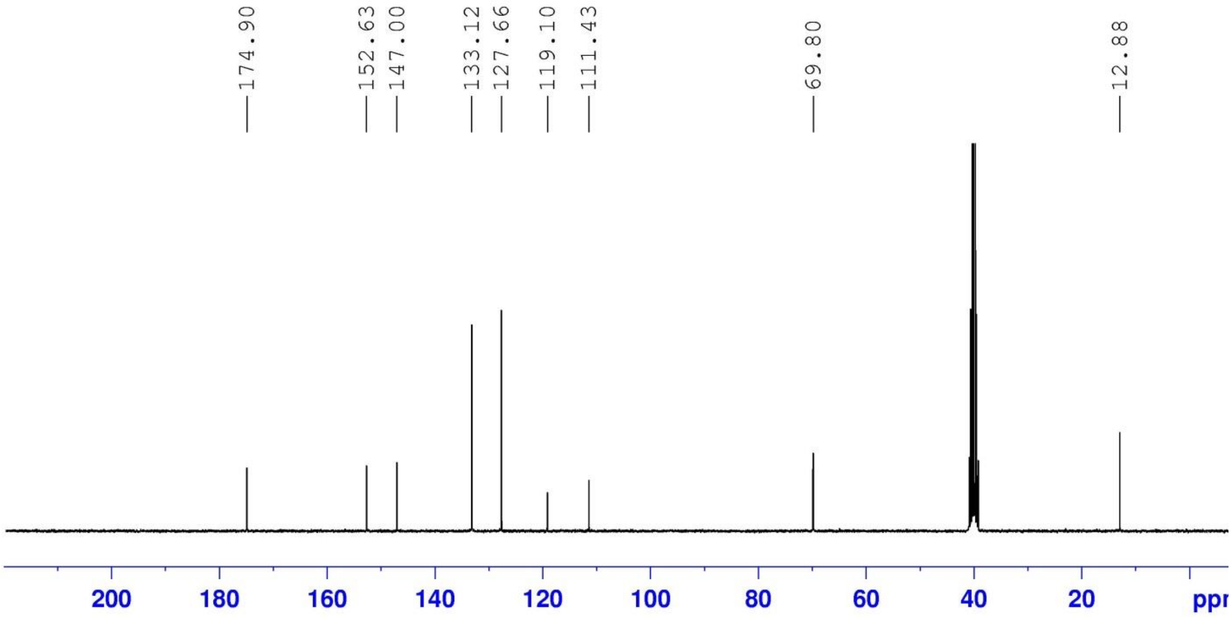

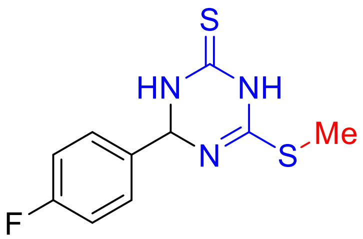

4-(4-Fluorophenyl)-6-(methylthio)-3,4-dihydro-1,3,5-triazine-2(1*H*)-thione (6oa)

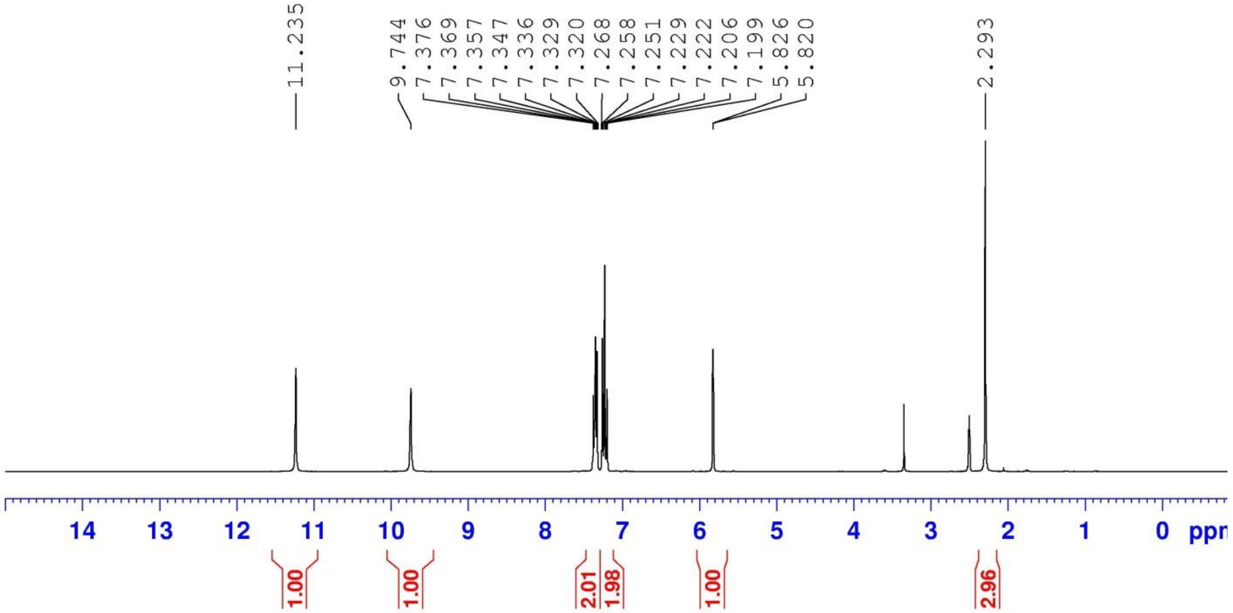

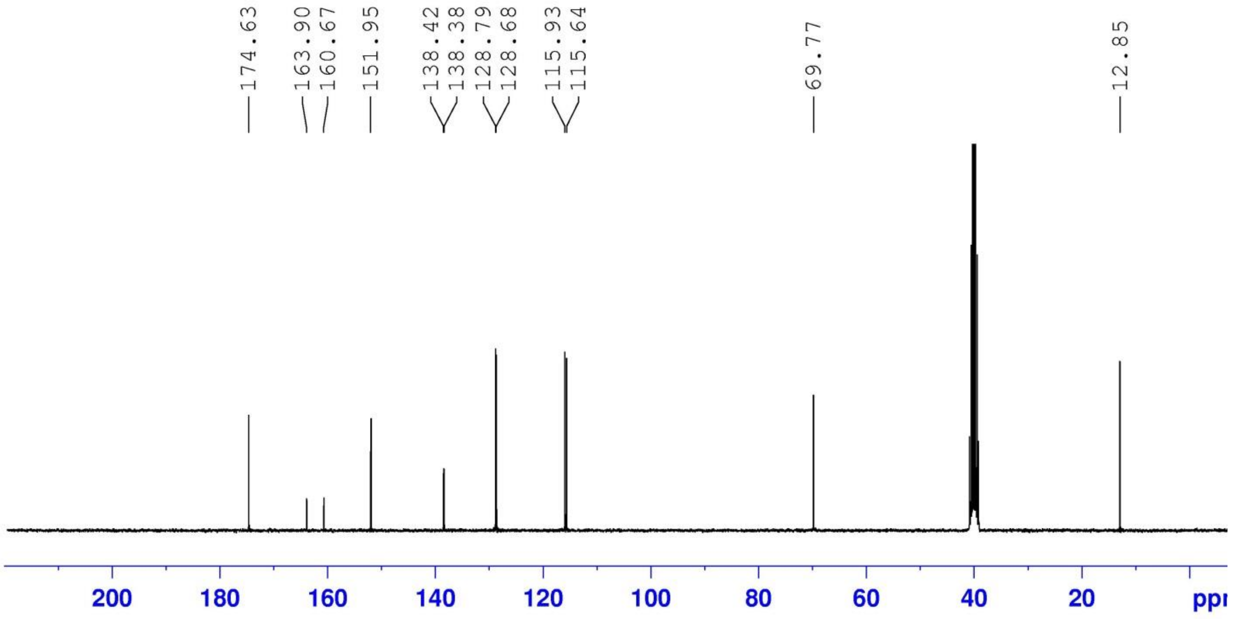

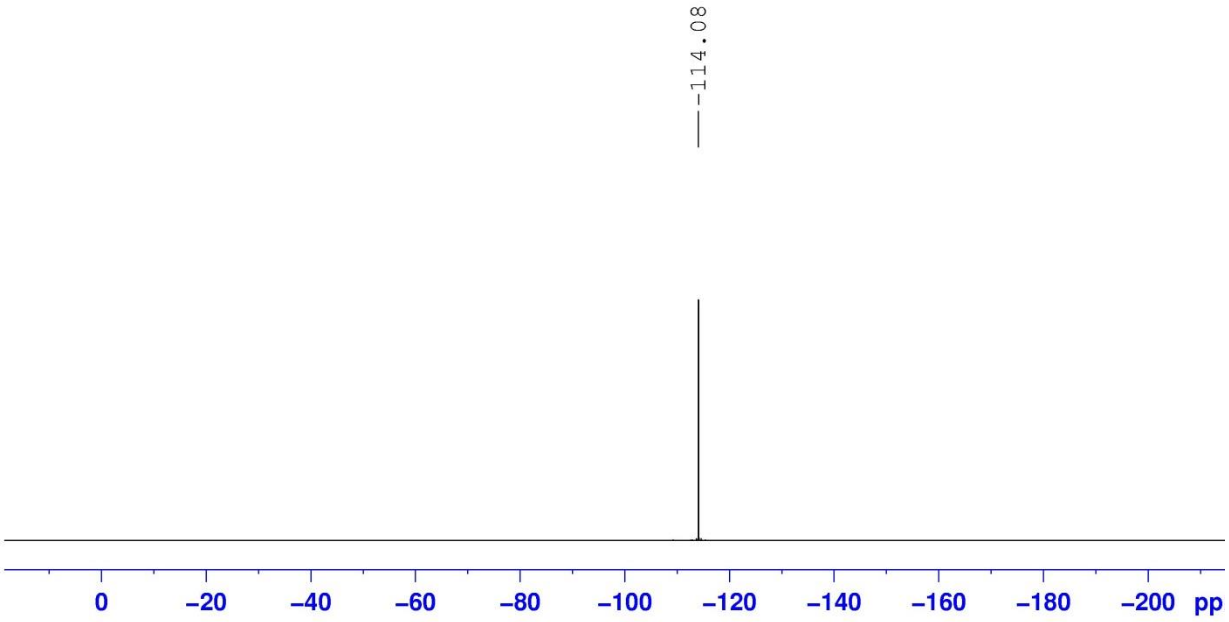
-114.08
0
−20
−40
−60
−80
−100
−120
−140
−160
−180
−200
pp

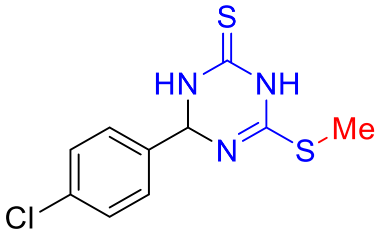

4-(4-Chlorophenyl)-6-(methylthio)-3,4-dihydro-1,3,5-triazine-2(1*H*)-thione (6pa)

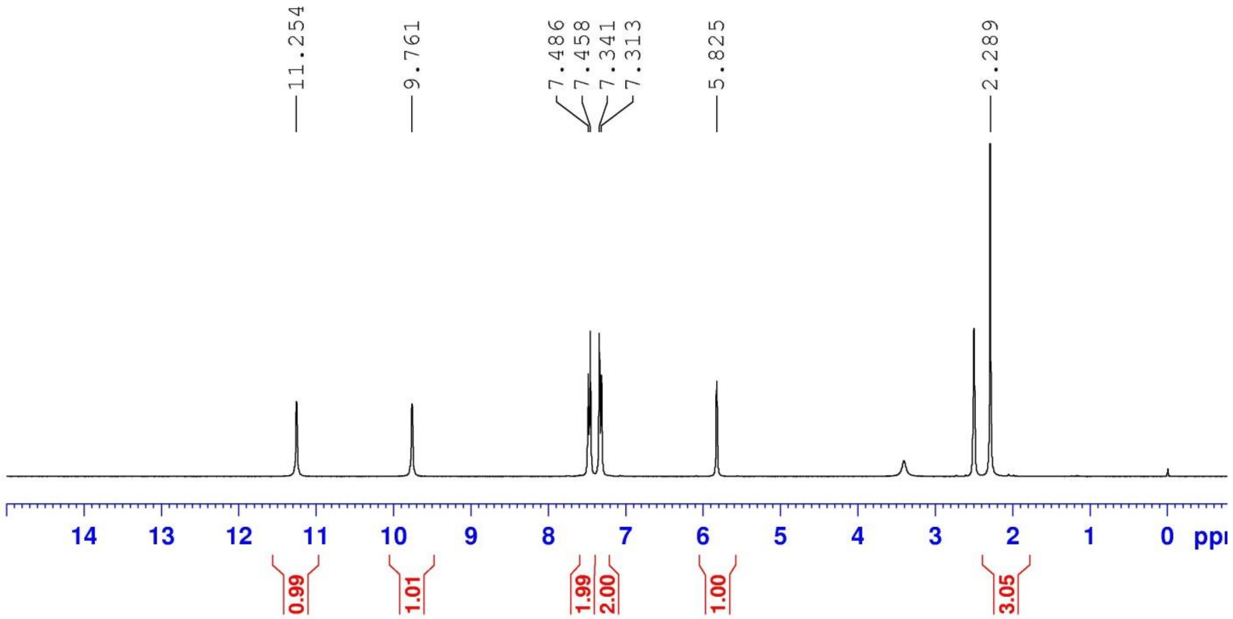

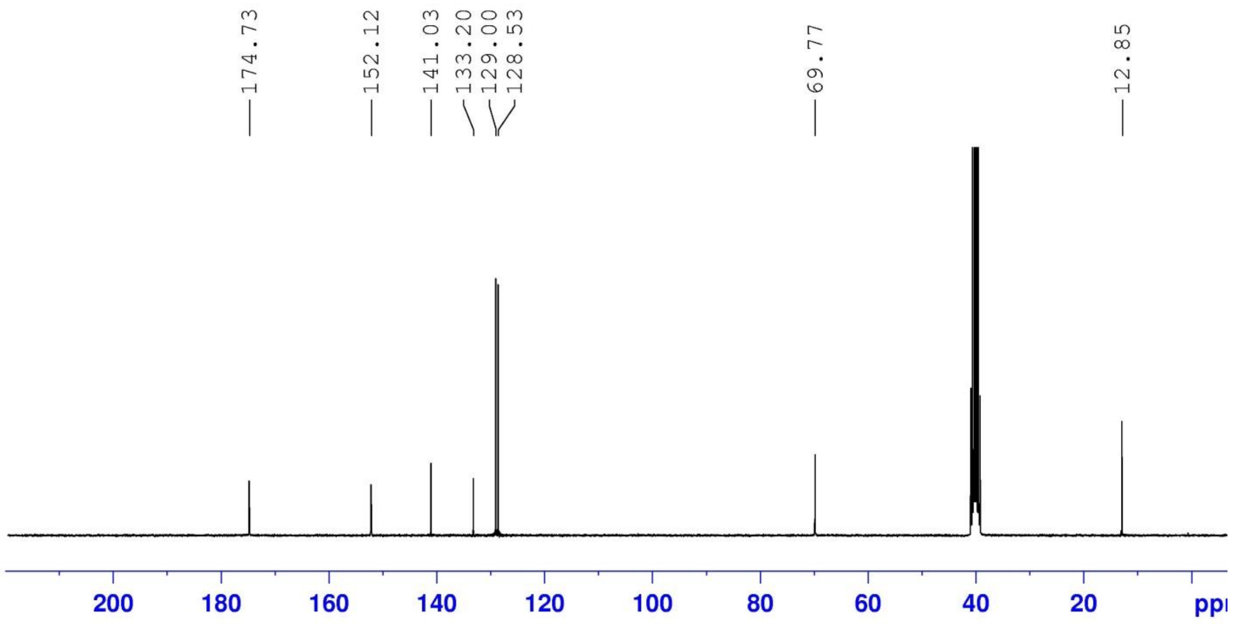

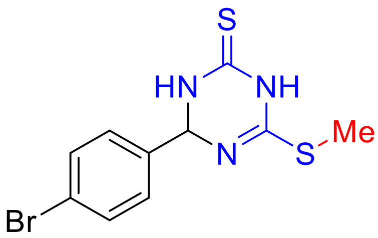

4-(4-Bromophenyl)-6-(methylthio)-3,4-dihydro-1,3,5-triazine-2(1*H*)-thione (6qa)

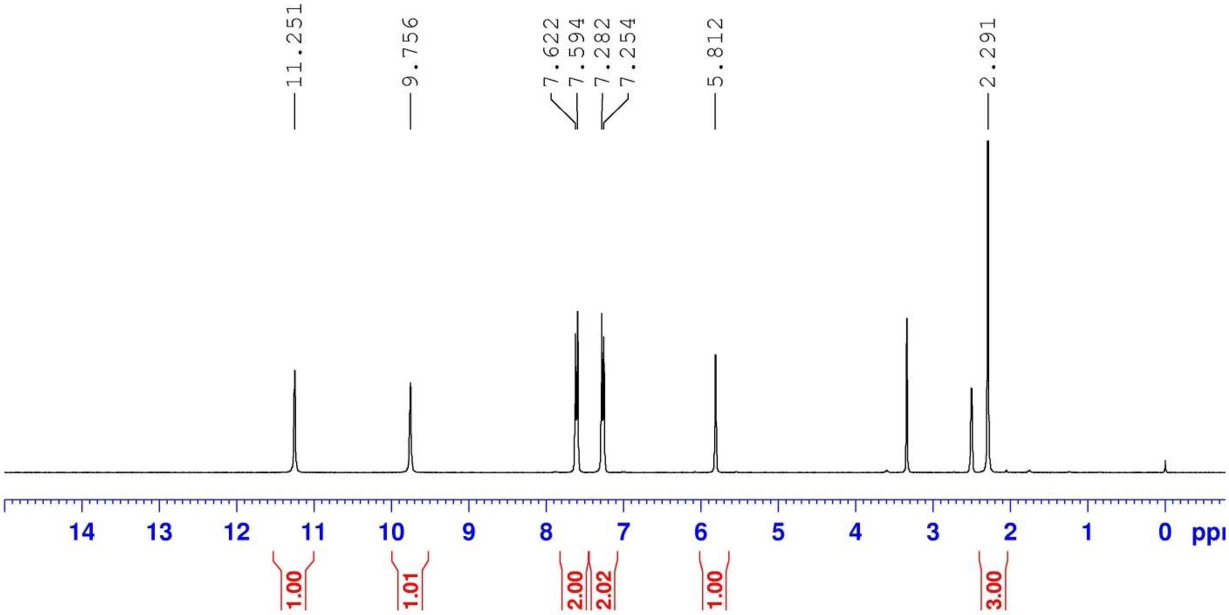

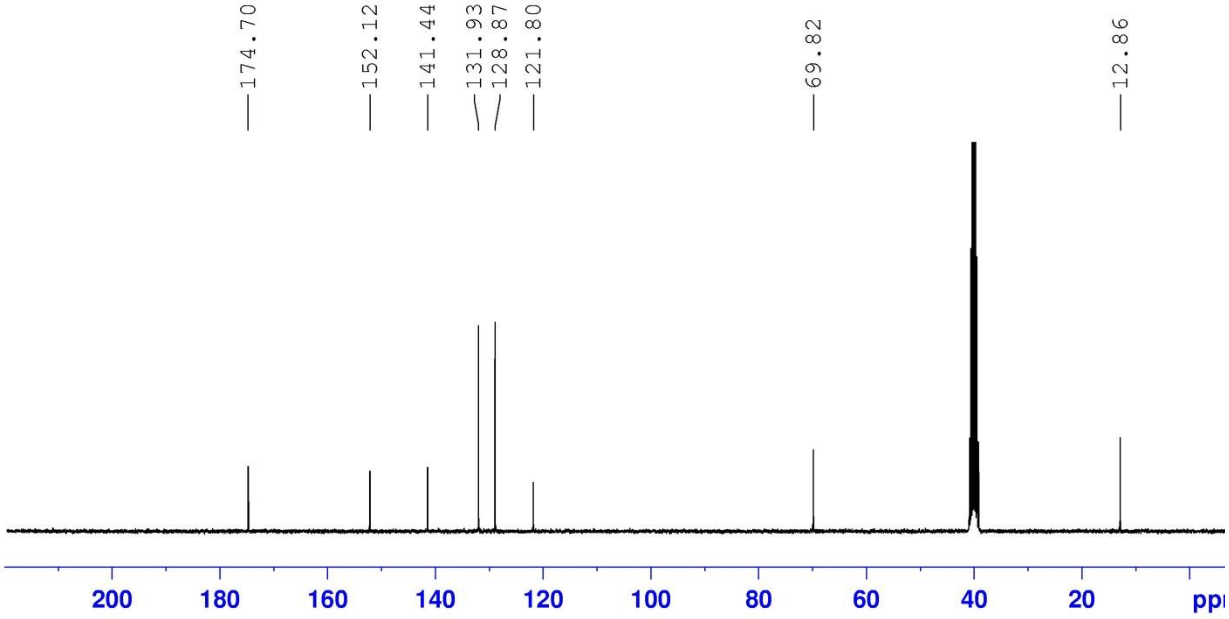

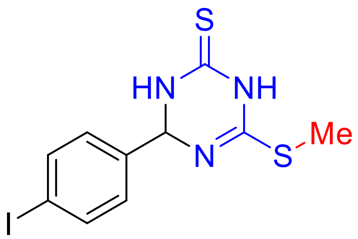

4-(4-Iodophenyl)-6-(methylthio)-3,4-dihydro-1,3,5-triazine-2(1*H*)-thione (6ra)

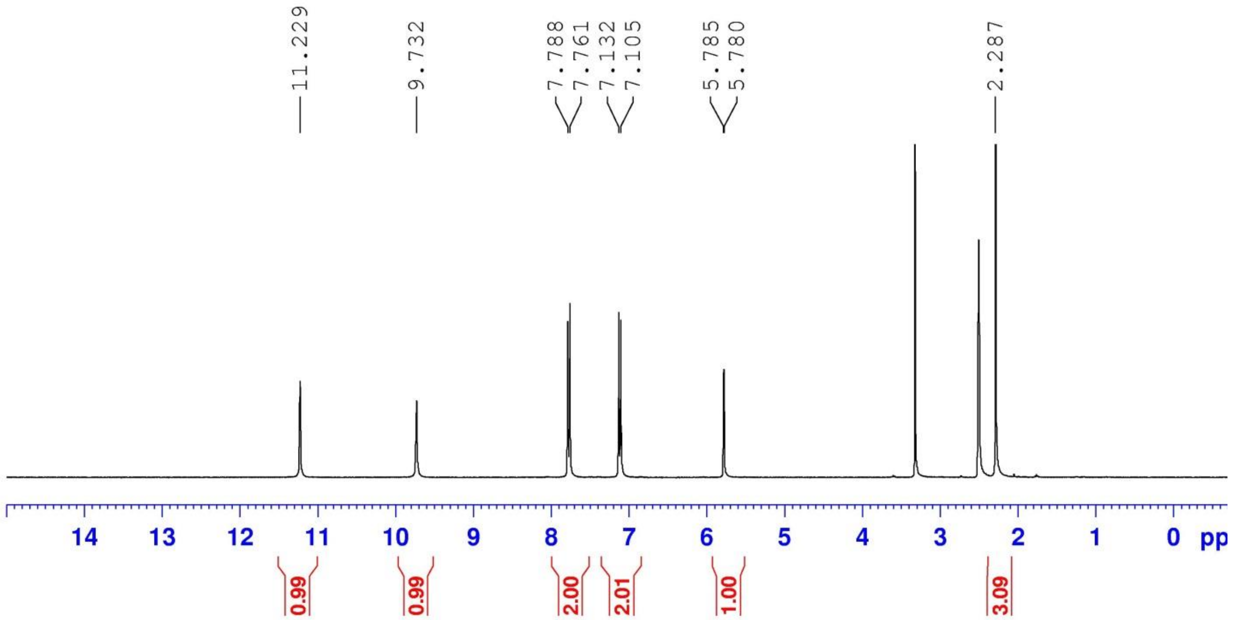

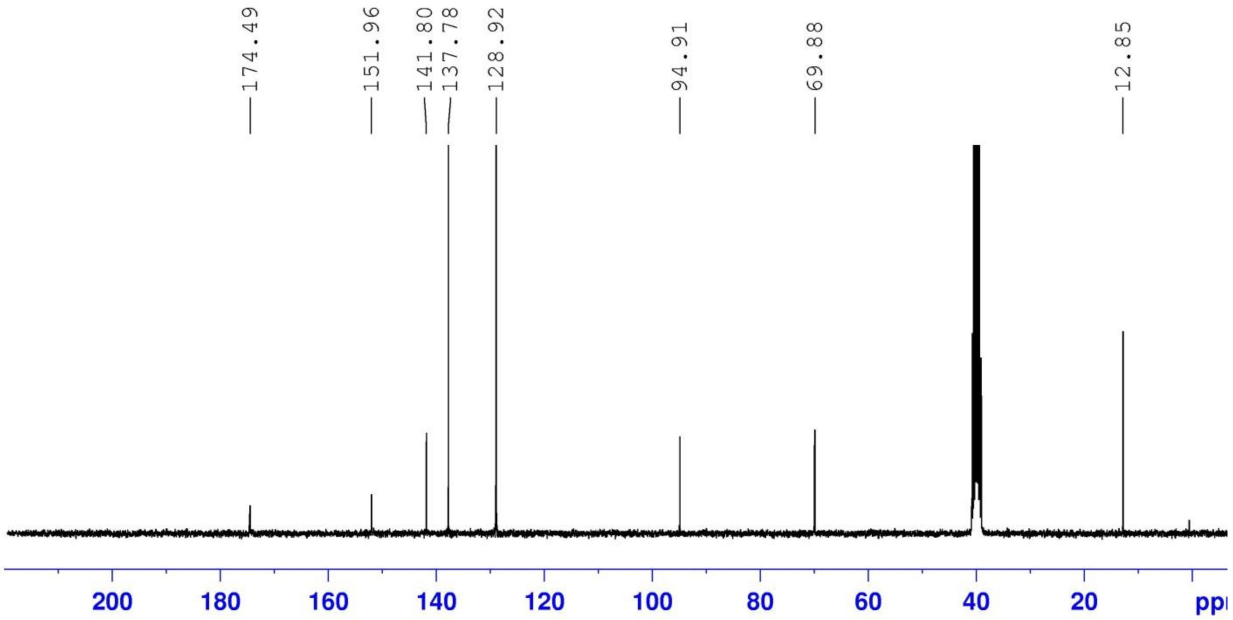

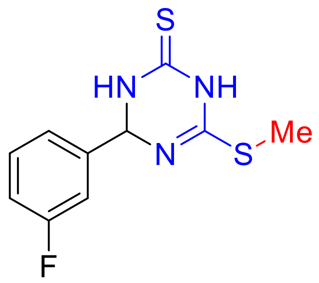

4-(3-Fluorophenyl)-6-(methylthio)-3,4-dihydro-1,3,5-triazine-2(1*H*)-thione (6sa)

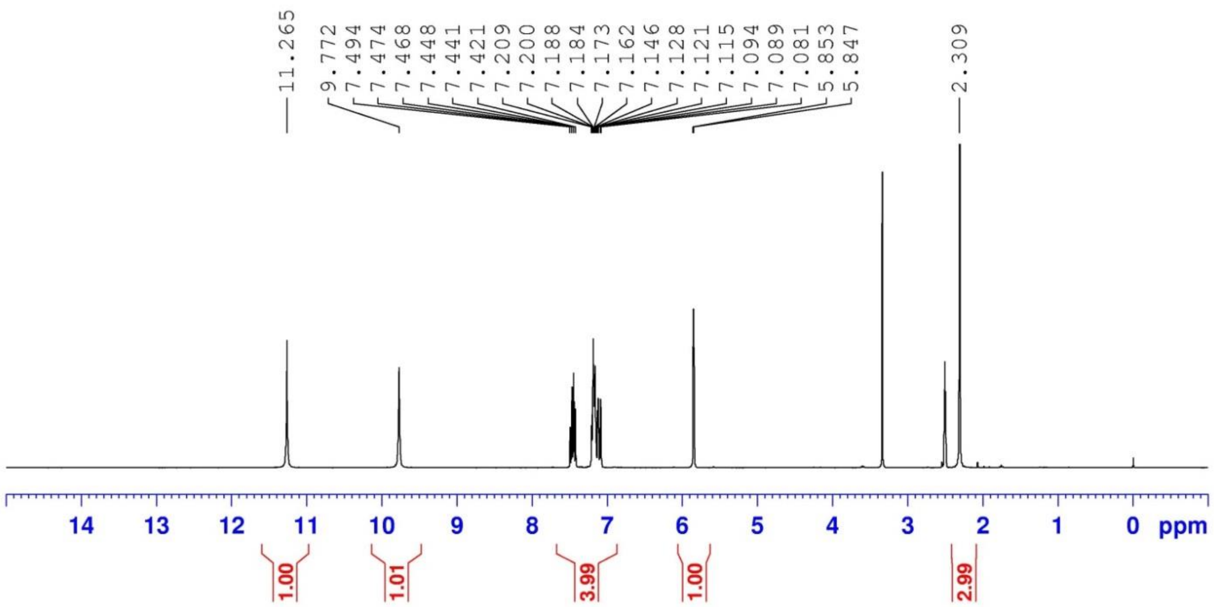

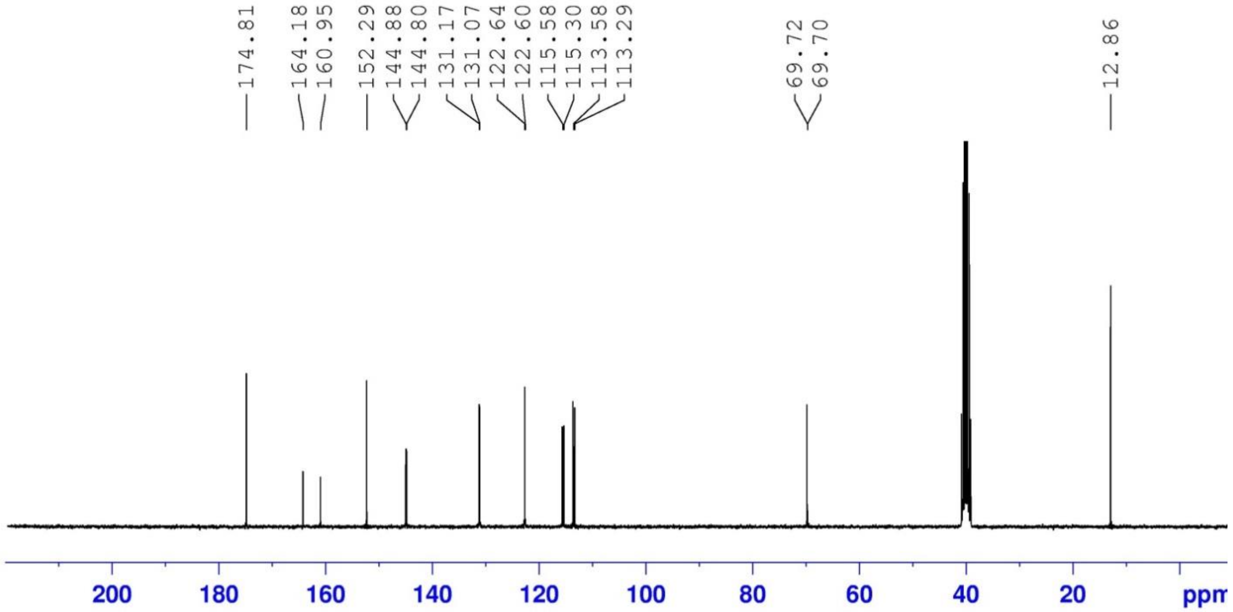

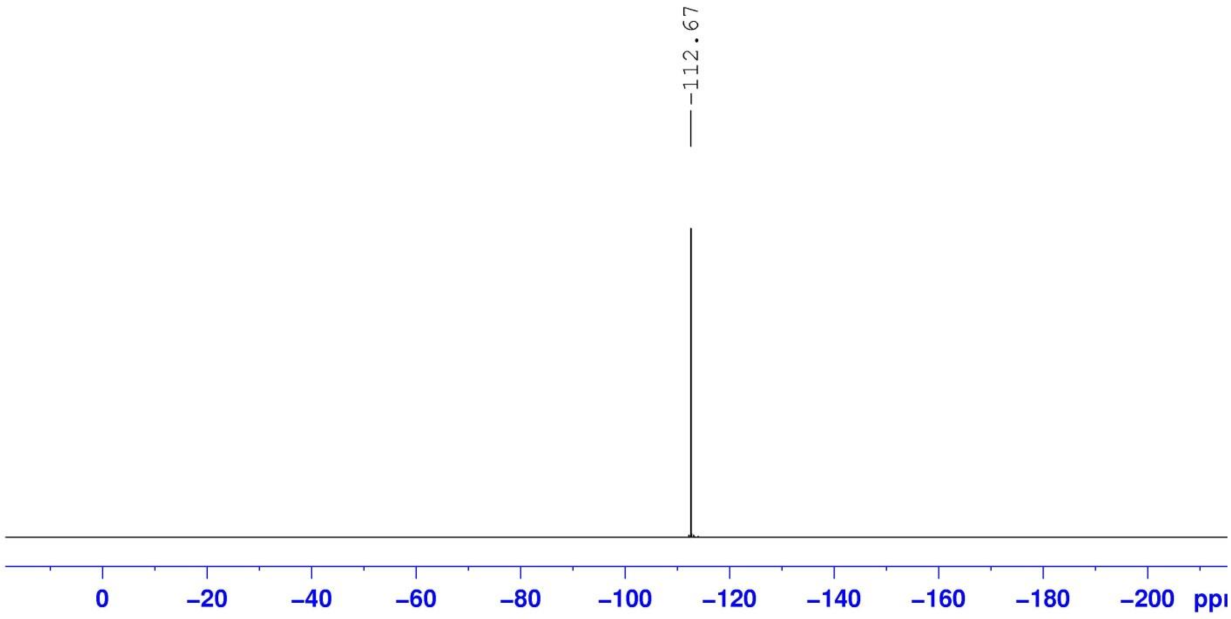

-112.67
0
–20
–40
–60
–80
–100
–120
–140
–160
–180
–200
ppm

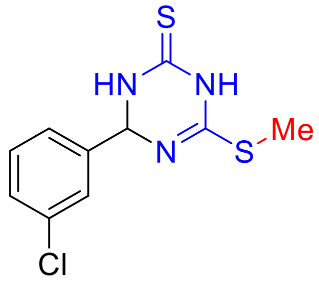

4-(3-Chlorophenyl)-6-(methylthio)-3,4-dihydro-1,3,5-triazine-2(1*H*)-thione (6ta)

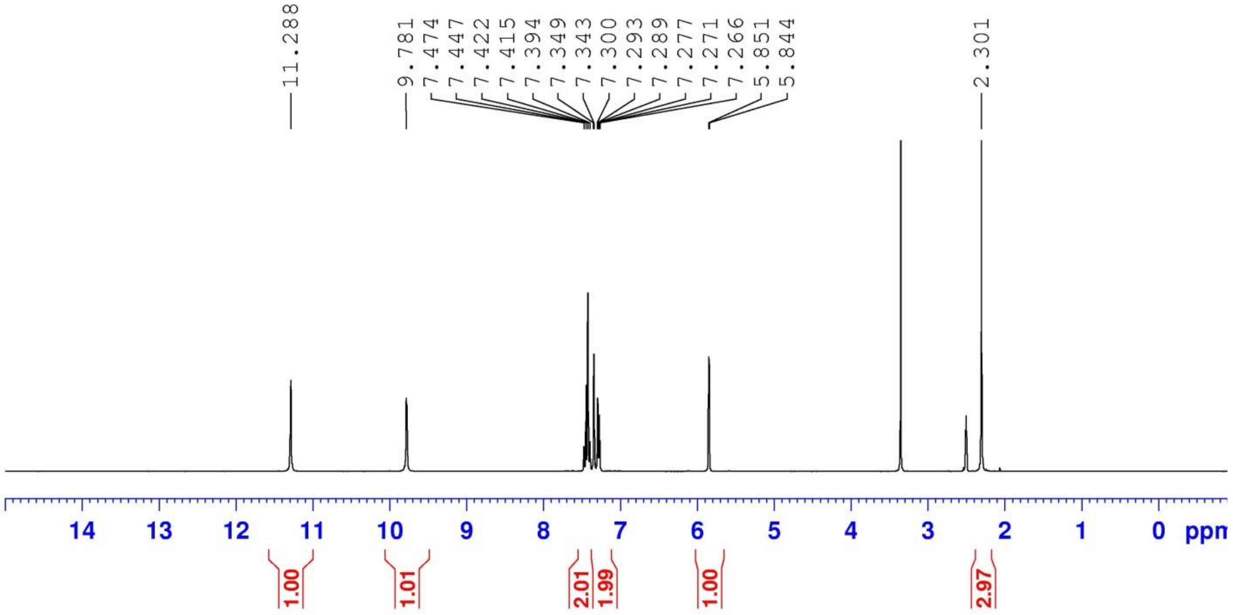

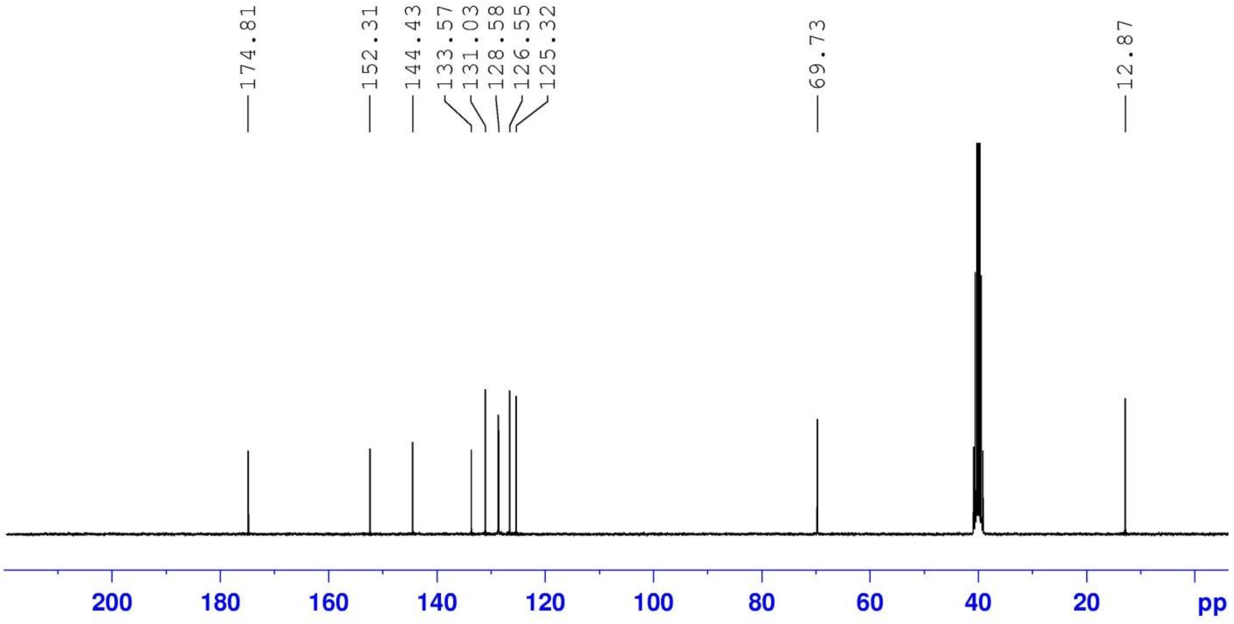

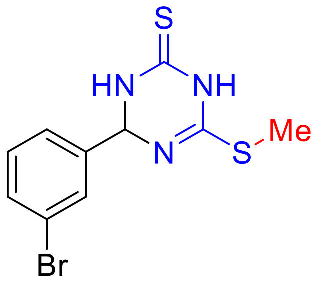

4-(3-Bromophenyl)-6-(methylthio)-3,4-dihydro-1,3,5-triazine-2(1*H*)-thione (6ua)

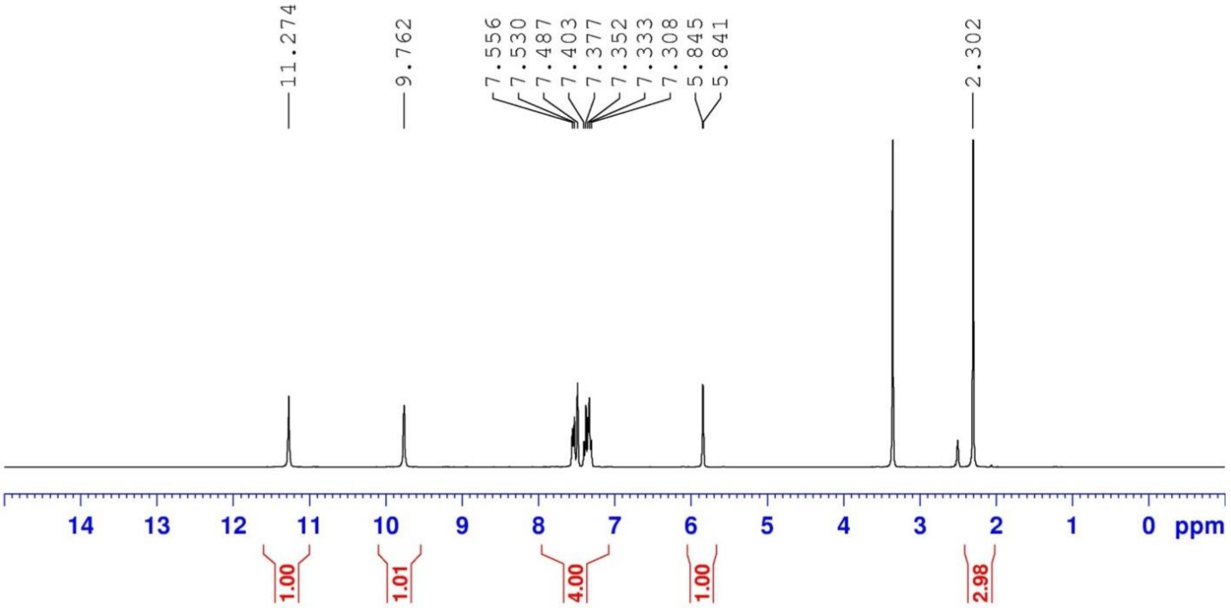

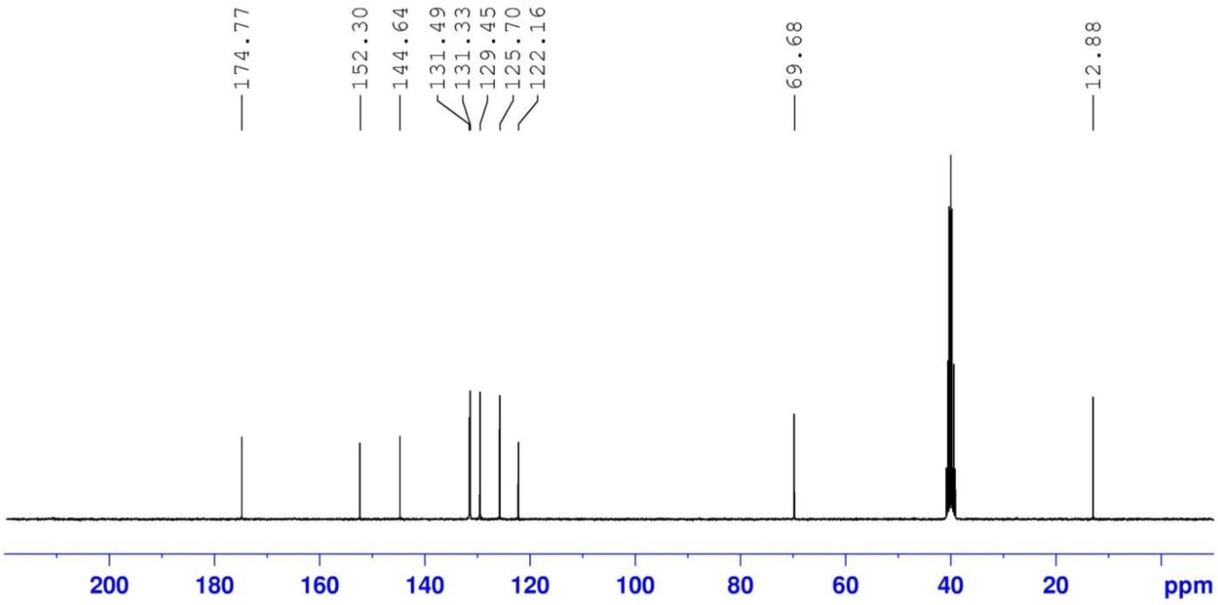

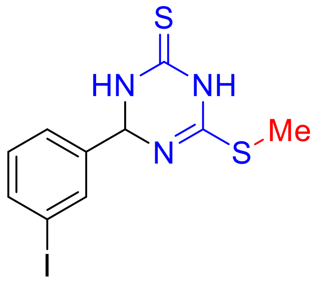

4-(3-Iodophenyl)-6-(methylthio)-3,4-dihydro-1,3,5-triazine-2(1*H*)-thione (6va)

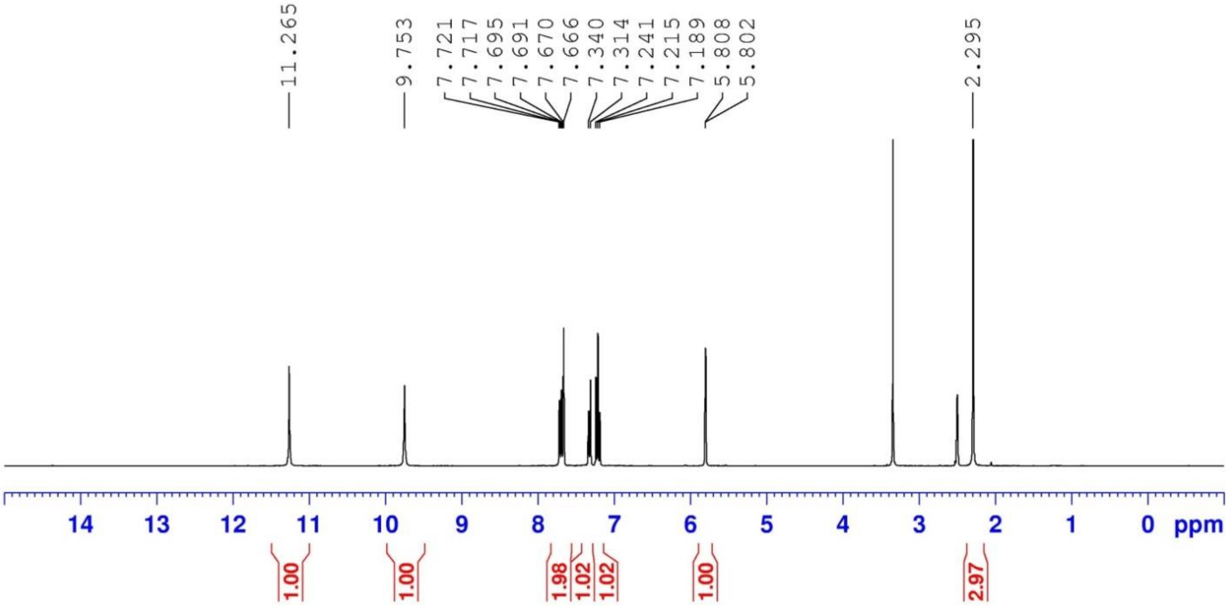

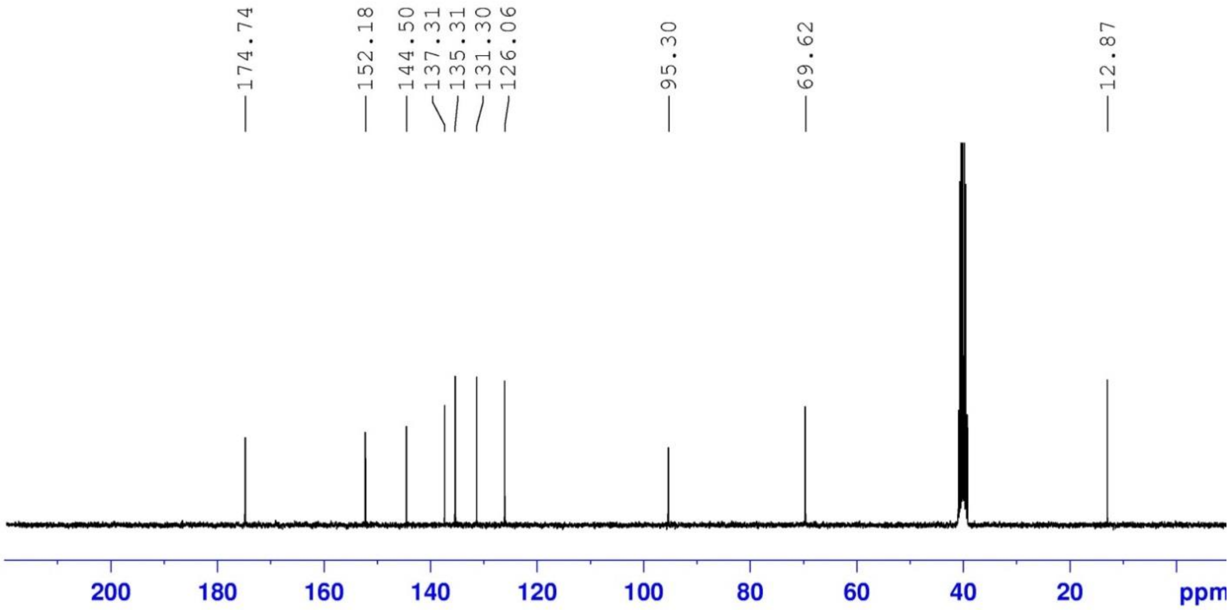

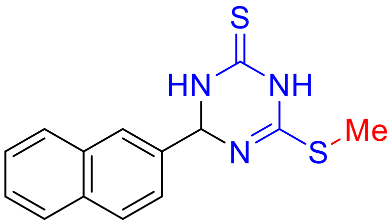

6-(Methylthio)-4-(naphthalen-2-yl)-3,4-dihydro-1,3,5-triazine-2(1*H*)-thione (6wa)

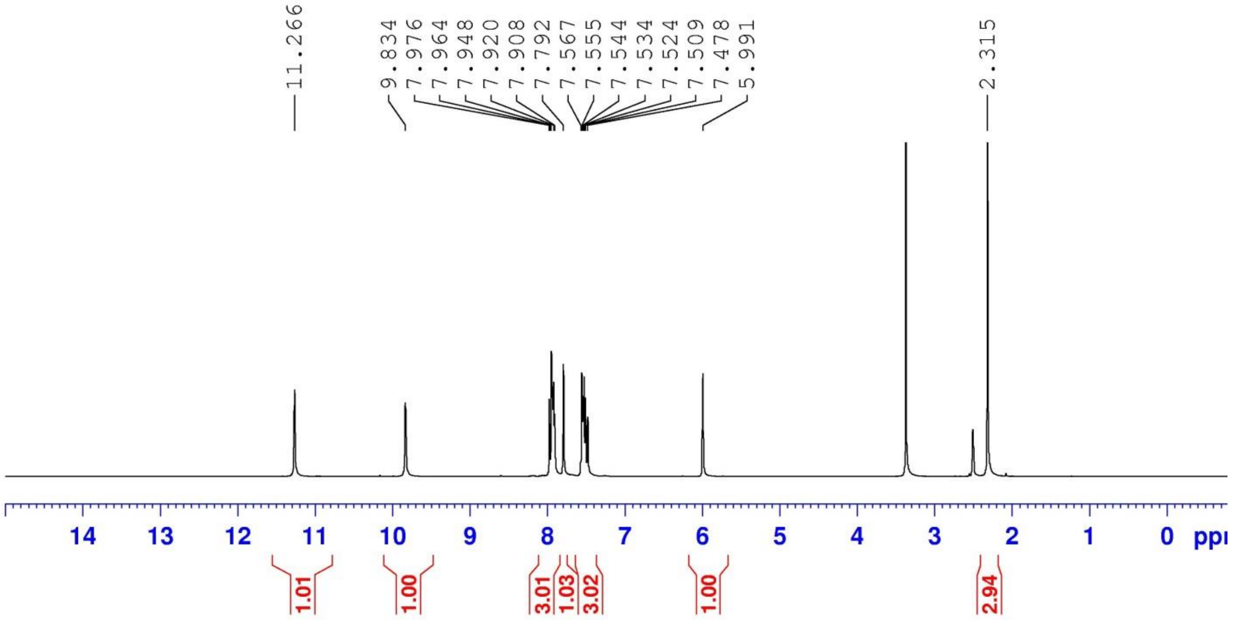

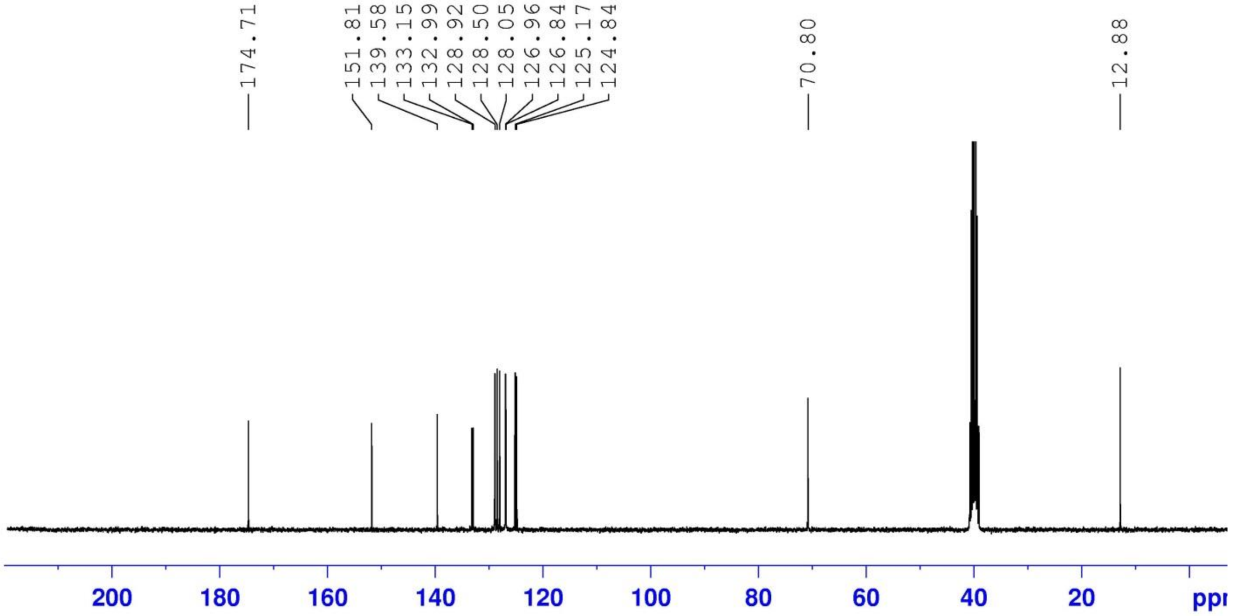

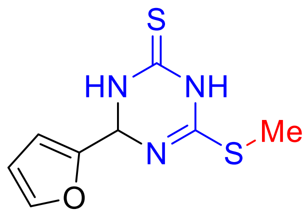

4-(Furan-2-yl)-6-(methylthio)-3,4-dihydro-1,3,5-triazine-2(1*H*)-thione (6xa)

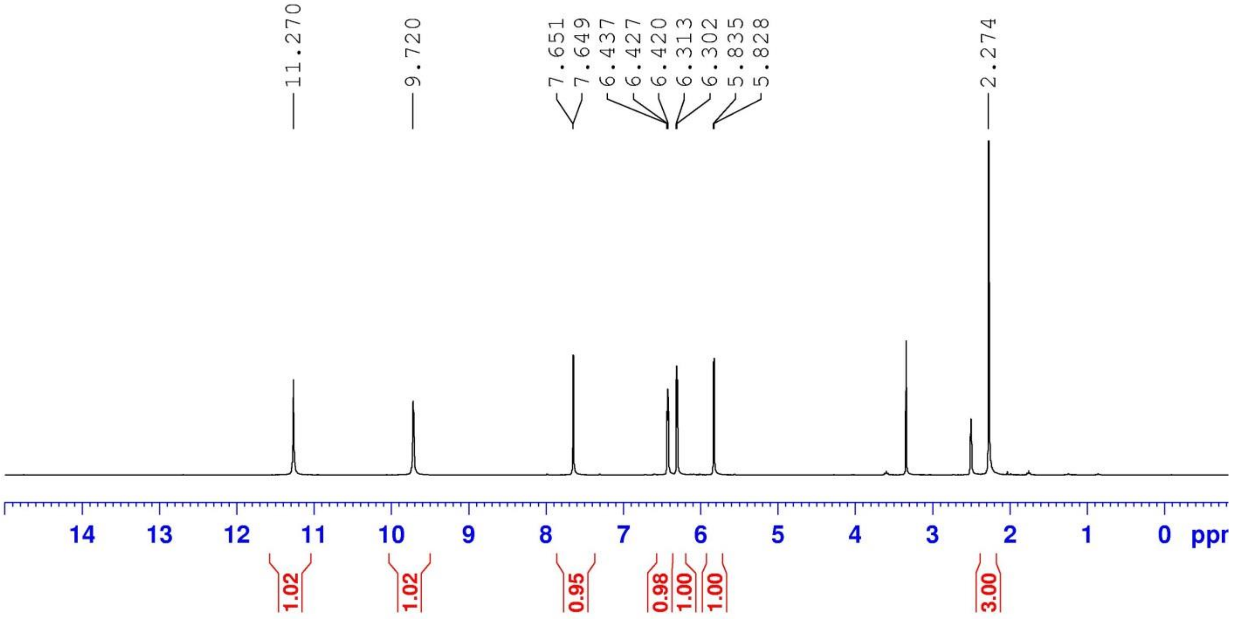

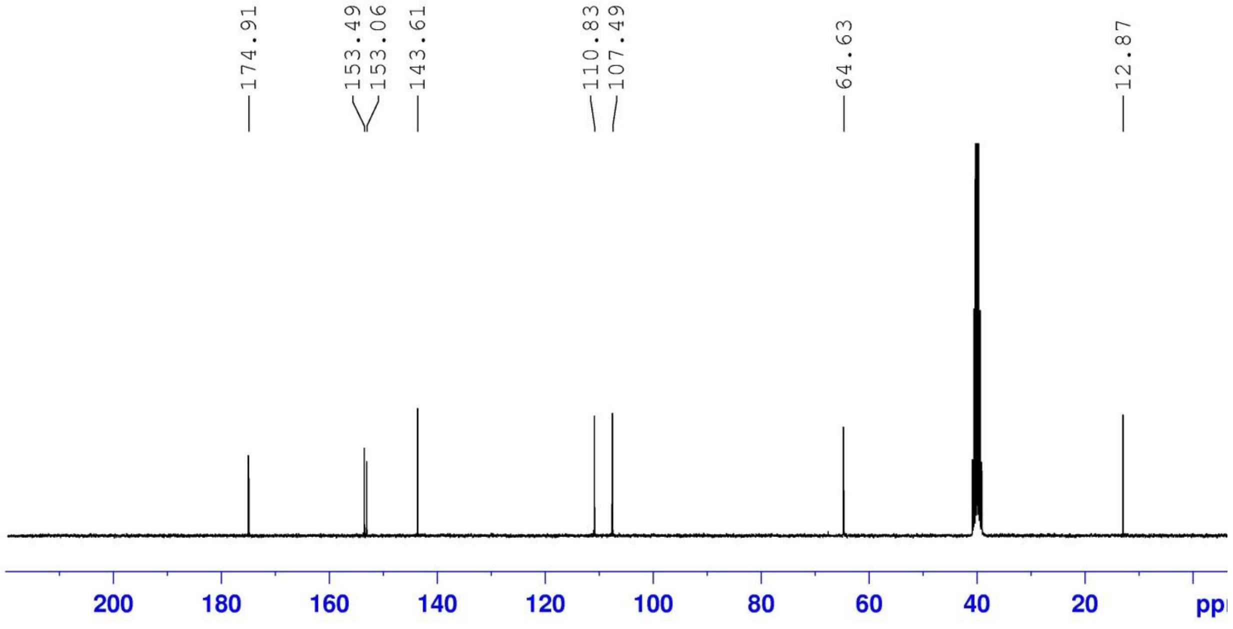

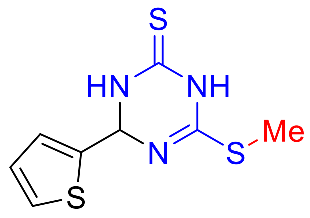

6-(Methylthio)-4-(thiophen-2-yl)-3,4-dihydro-1,3,5-triazine-2(1*H*)-thione (6ya)

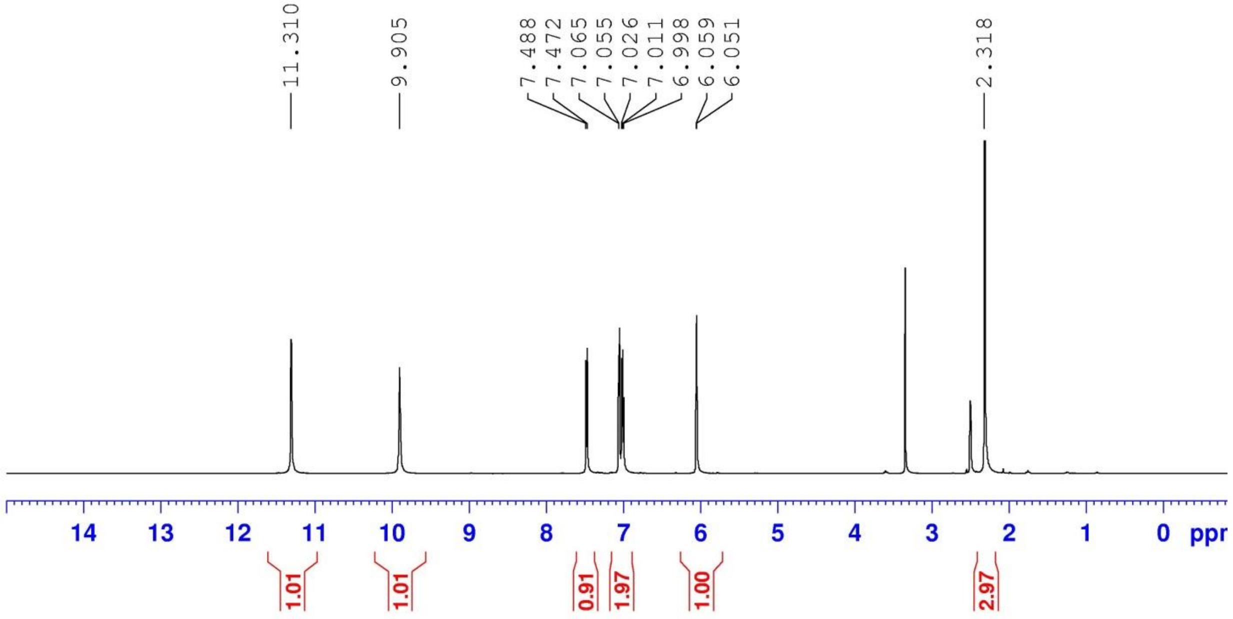

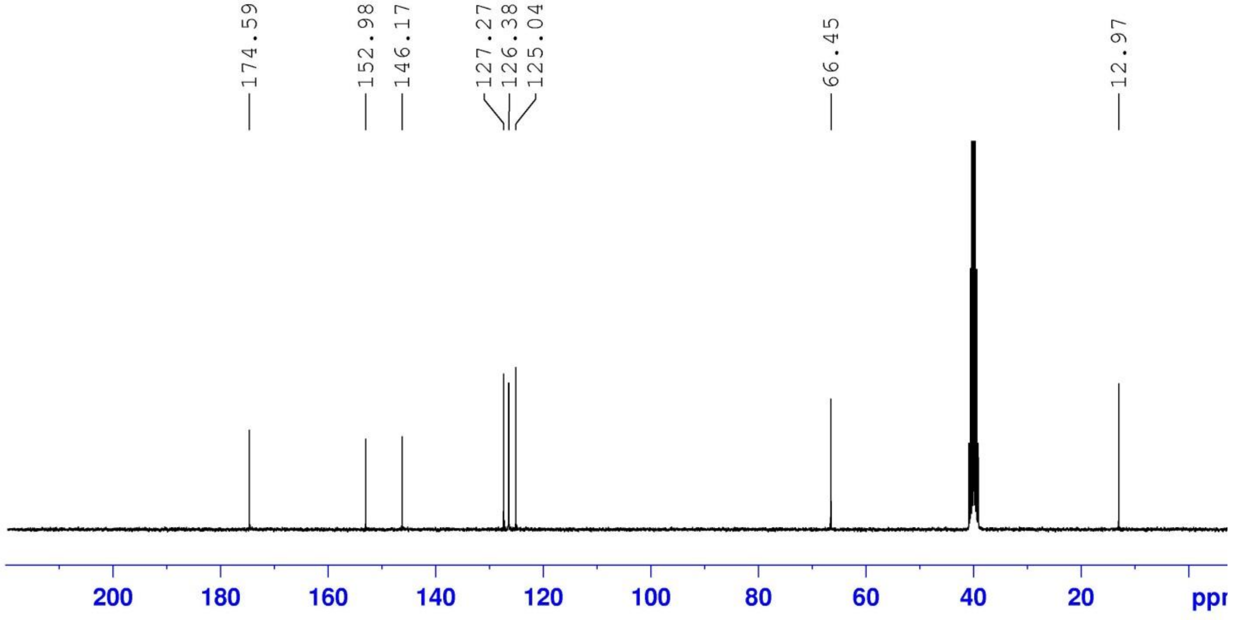

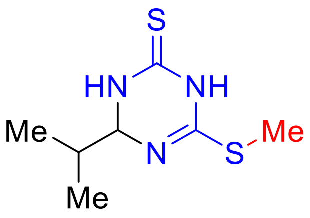

4-Isopropyl-6-(methylthio)-3,4-dihydro-1,3,5-triazine-2(1*H*)-thione (6za)

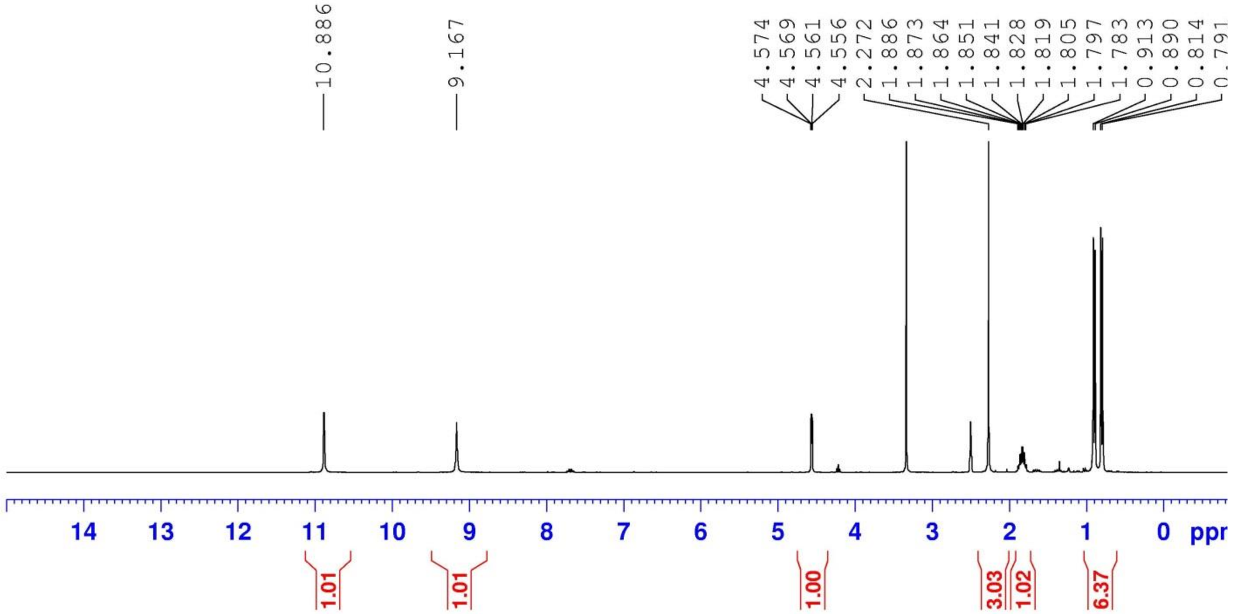

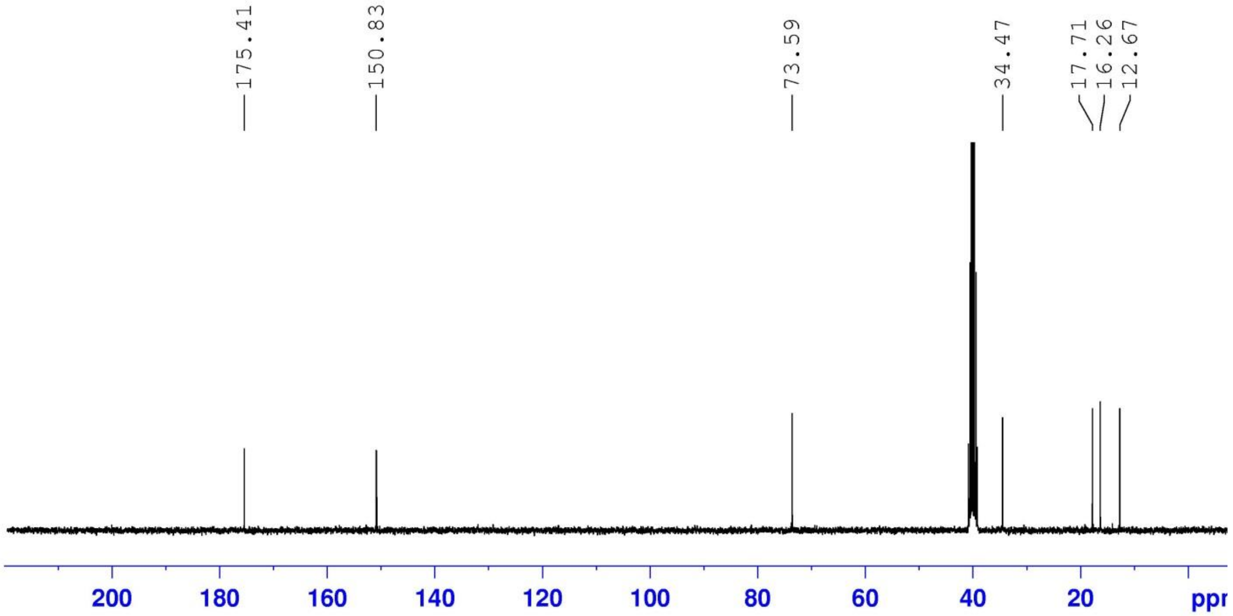

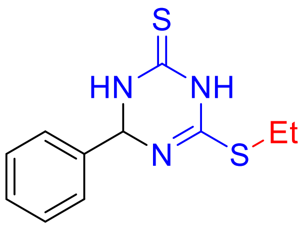

6-(Ethylthio)-4-phenyl-3,4-dihydro-1,3,5-triazine-2(1*H*)-thione (6ab)

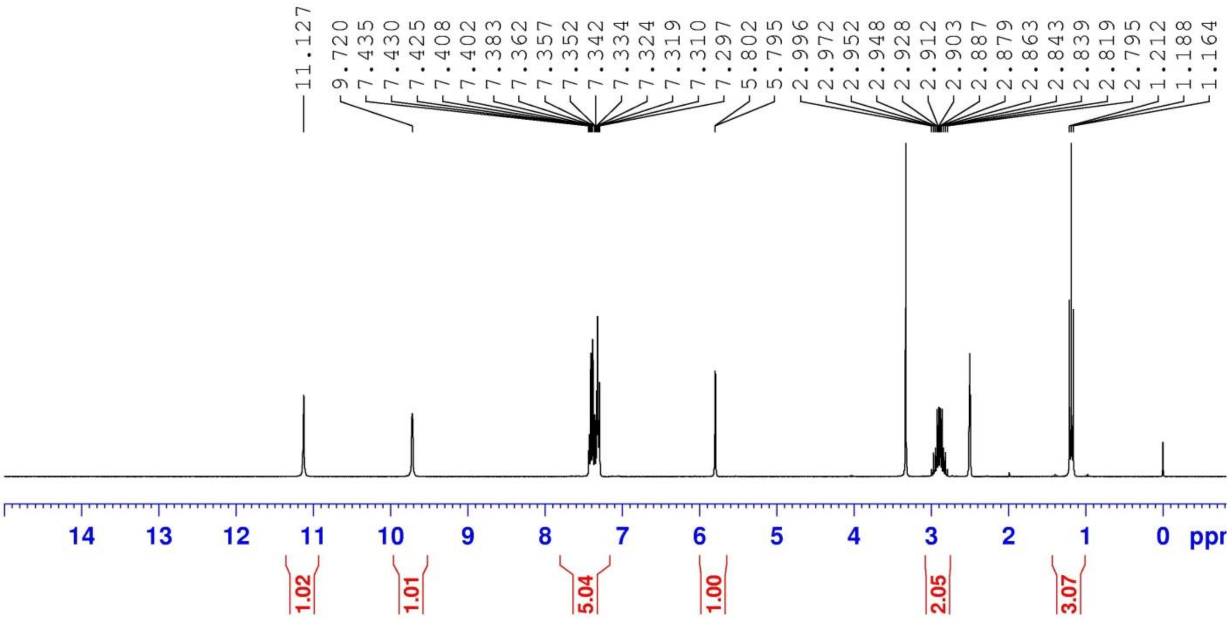

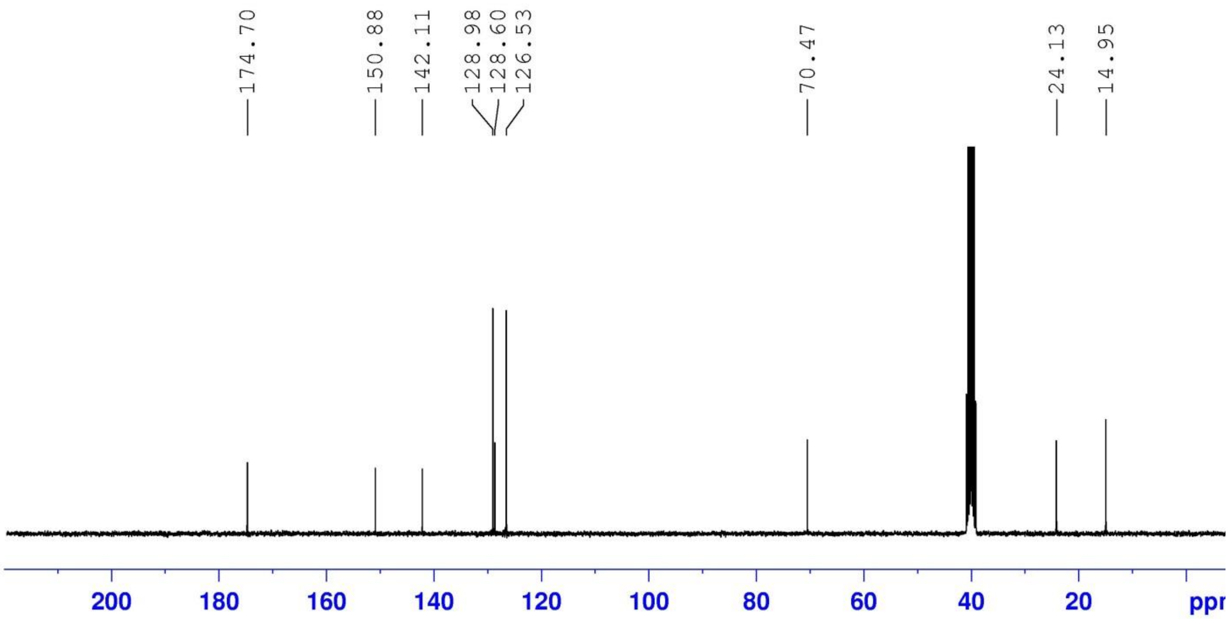

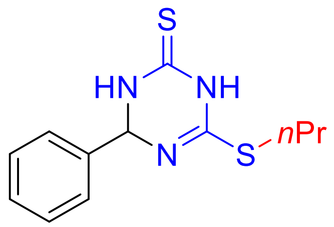

4-Phenyl-6-(propylthio)-3,4-dihydro-1,3,5-triazine-2(1*H*)-thione (6ac)

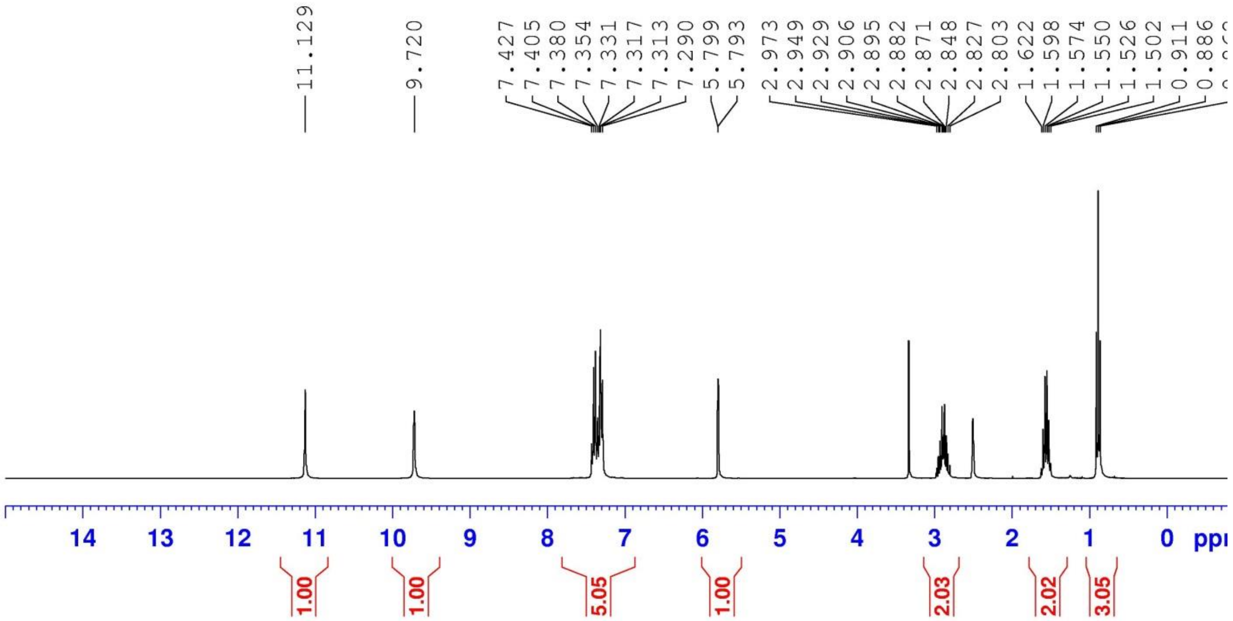

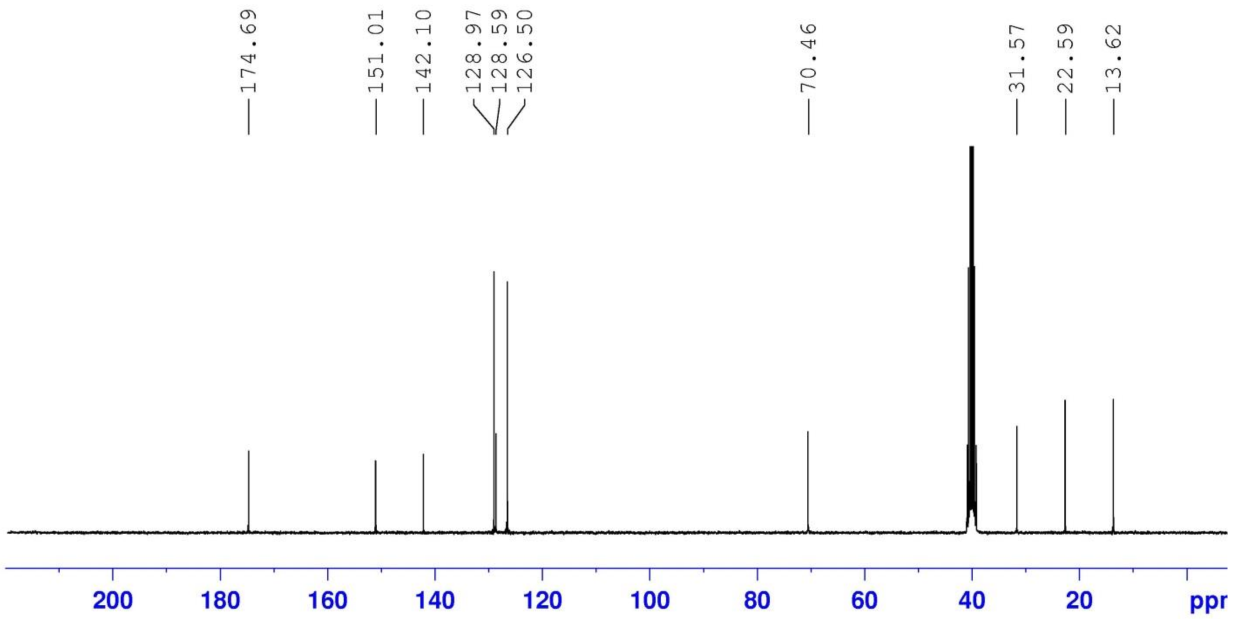

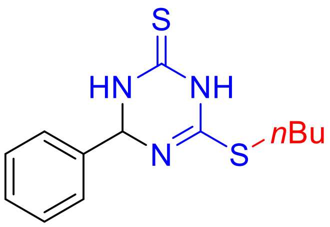

6-(Butylthio)-4-phenyl-3,4-dihydro-1,3,5-triazine-2(1*H*)-thione (6ad)

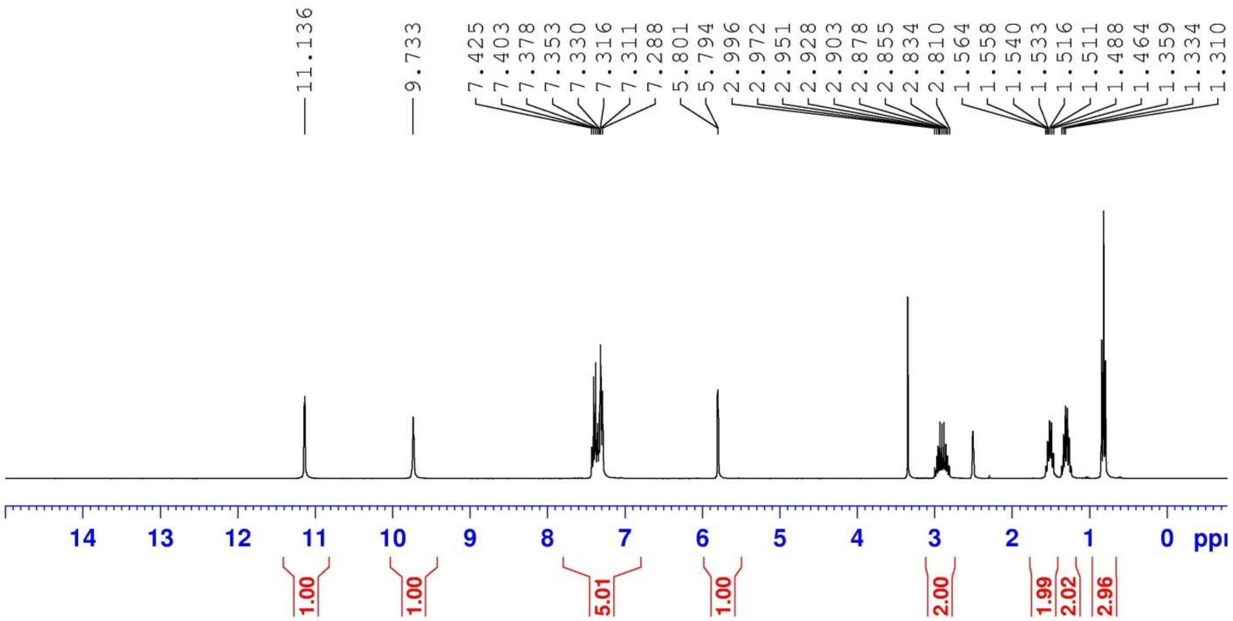

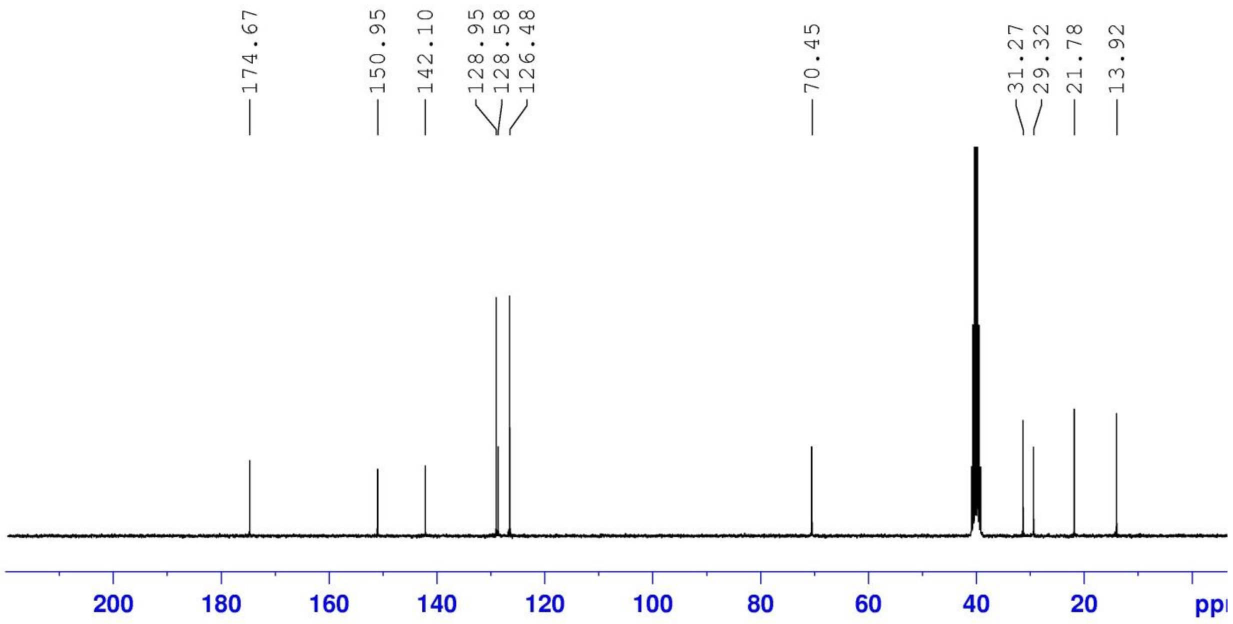

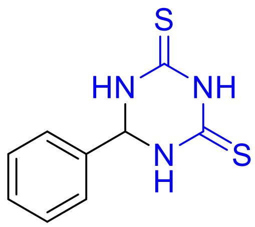

6-Phenyl-1,3,5-triazinane-2,4-dithione (7)

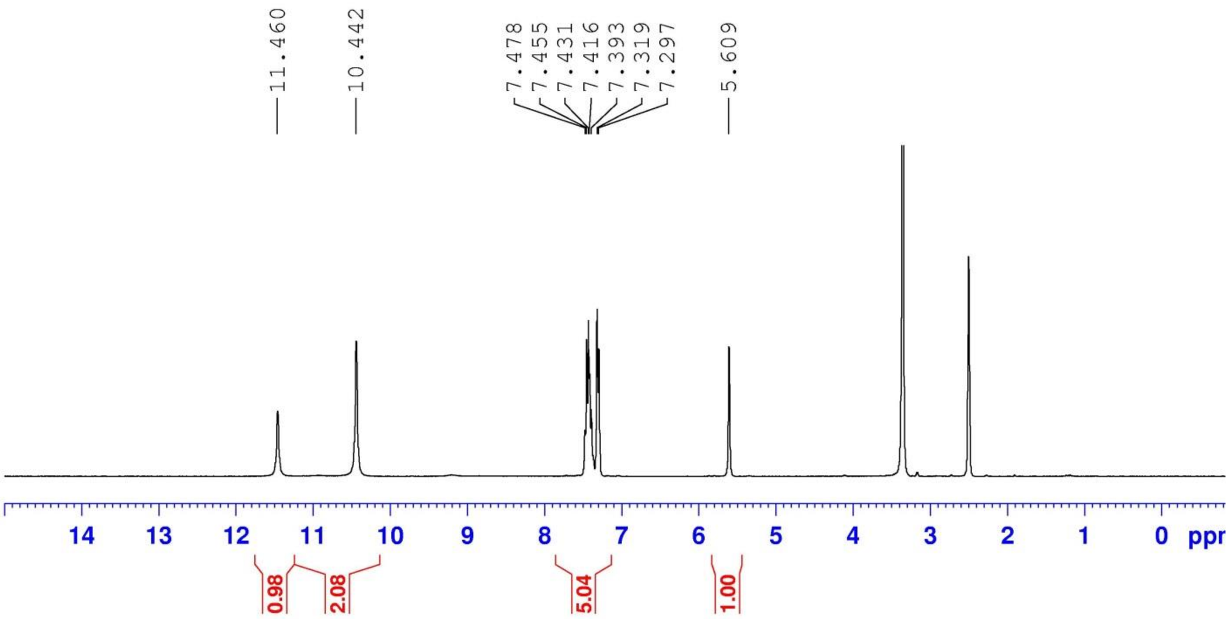

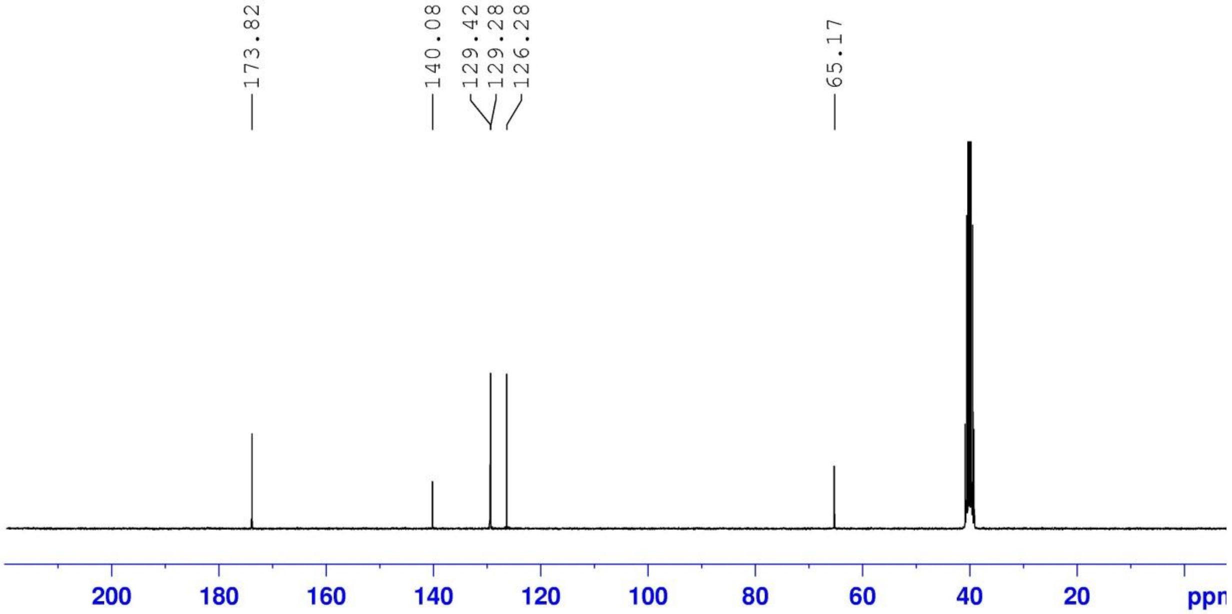

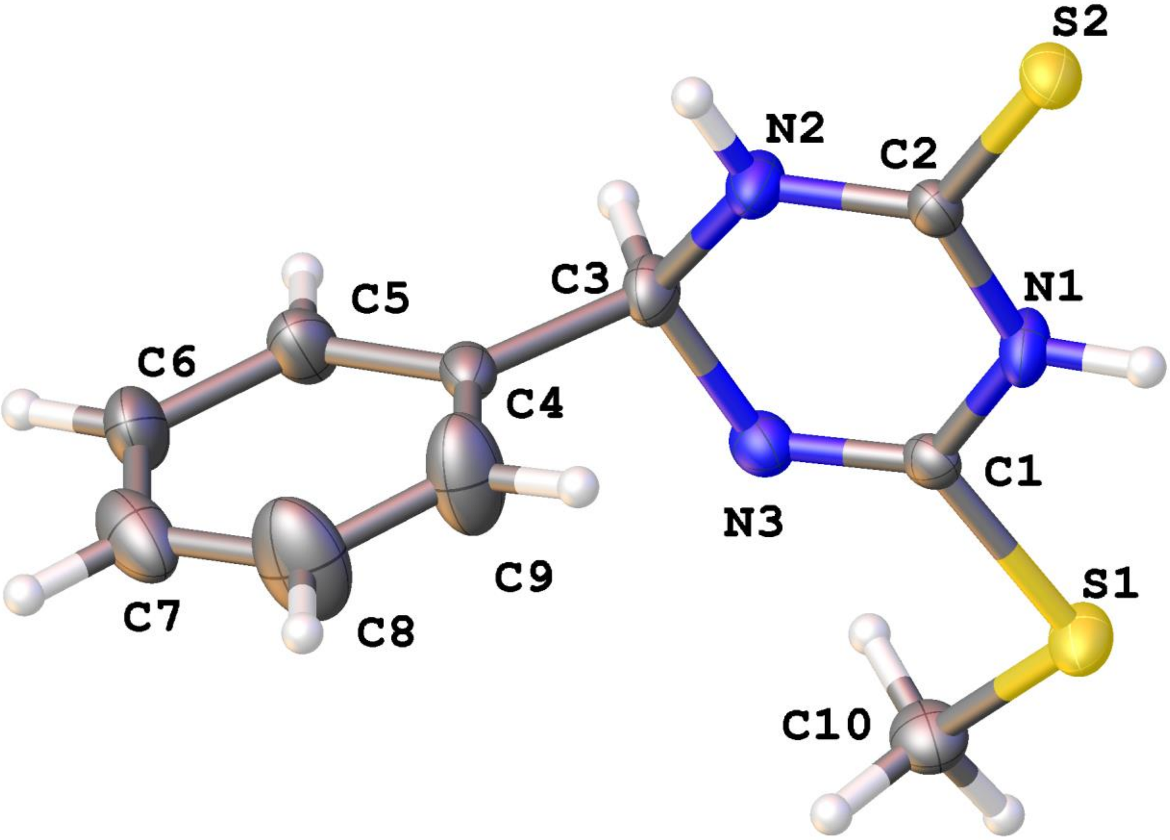

Figure S1: X-ray structure of 6-(methylthio)-4-phenyl-3,4-dihydro-1,3,5-triazine-2(1*H*)-thione **(6aa)** with thermal ellipsoids at 50% probability (CCDC 1991859).

Table S1: Crystal data and structure refinement for **6aa**.

Identification code	190128kgf_0m_tw	
Empirical formula	C10 H11 N3 S2	
Formula weight	237.34	
Temperature	100 K	
Wavelength	0.71073 Å	
Crystal system	Triclinic	
Space group	P-1	
Unit cell dimensions	a = 5.258(4) Å	α= 99.991(9)°.
	b = 8.810(6) Å	β= 98.639(8)°.
	c = 12.257(9) Å	γ = 95.953(8)°.
Volume	547.9(7) $Å^3$	
Z	2	
Density (calculated)	1.439 Mg/m^3	
Absorption coefficient	0.454 mm^{-1}	
F(000)	248	
Crystal size	0.26 x 0.24 x 0.23 mm^3	
Theta range for data collection	2.369 to 27.528°.	
Index ranges	-6<=h<=6, -11<=k<=11, -5<=l<=15	
Reflections collected	2311	
Independent reflections	2311 [R(int) = ?]	
Completeness to theta = 25.242°	97.2 %	
Absorption correction	Semi-empirical from equivalents	
Max. and min. transmission	0.7456 and 0.5168	
Refinement method	Full-matrix least-squares on F^2	
Data / restraints / parameters	2311 / 0 / 138	
Goodness-of-fit on F^2	1.181	
Final R indices [I>2sigma(I)]	R1 = 0.0898, wR2 = 0.2374	
R indices (all data)	R1 = 0.0951, wR2 = 0.2394	
Extinction coefficient	n/a	
Largest diff. peak and hole	0.814 and -0.660 e.$Å^{-3}$	

Table S2: Atomic coordinates (× 10^4) and equivalent isotropic displacement parameters ($Å^2$ × 10^3) for **6aa**. U(eq) is defined as one third of the trace of the orthogonalized U^{ij} tensor.

	x	y	z	U(eq)
S(1)	3549(3)	9807(2)	3049(2)	29(1)
S(2)	10811(3)	7591(2)	5475(1)	24(1)
N(1)	6845(11)	8361(6)	4176(5)	26(1)
N(2)	6958(11)	5772(6)	4008(5)	27(1)
N(3)	3473(11)	6738(6)	2871(5)	24(1)
C(1)	4612(12)	8065(7)	3354(5)	19(1)
C(2)	8070(12)	7200(7)	4500(5)	20(1)
C(3)	4504(13)	5373(7)	3194(6)	25(1)
C(4)	4848(13)	4270(7)	2134(5)	24(1)
C(5)	3055(16)	2953(9)	1723(6)	35(2)
C(6)	3267(18)	1983(9)	719(7)	42(2)
C(7)	5177(17)	2337(10)	138(7)	43(2)
C(8)	6980(20)	3642(13)	559(9)	62(3)
C(9)	6821(18)	4587(11)	1564(9)	54(2)
C(10)	515(14)	9022(9)	2123(6)	32(2)

Table S3: Bond lengths [Å] and angles [°] for **6aa**.

S(1)-C(1)	1.766(6)
S(1)-C(10)	1.807(7)
S(2)-C(2)	1.692(7)
N(1)-H(1)	0.8800
N(1)-C(1)	1.397(8)
N(1)-C(2)	1.348(8)
N(2)-H(2)	0.8800
N(2)-C(2)	1.329(8)
N(2)-C(3)	1.477(8)
N(3)-C(1)	1.259(8)
N(3)-C(3)	1.460(8)
C(3)-H(3)	1.0000
C(3)-C(4)	1.528(9)
C(4)-C(5)	1.384(10)
C(4)-C(9)	1.367(12)
C(5)-H(5)	0.9500
C(5)-C(6)	1.397(11)
C(6)-H(6)	0.9500
C(6)-C(7)	1.357(13)
C(7)-H(7)	0.9500
C(7)-C(8)	1.381(14)
C(8)-H(8)	0.9500
C(8)-C(9)	1.381(13)
C(9)-H(9)	0.9500
C(10)-H(10A)	0.9800
C(10)-H(10B)	0.9800
C(10)-H(10C)	0.9800
C(1)-S(1)-C(10)	99.8(3)
C(1)-N(1)-H(1)	119.1
C(2)-N(1)-H(1)	119.1
C(2)-N(1)-C(1)	121.7(5)
C(2)-N(2)-H(2)	117.1
C(2)-N(2)-C(3)	125.8(6)

C(3)-N(2)-H(2)	117.1
C(1)-N(3)-C(3)	118.5(5)
N(1)-C(1)-S(1)	111.5(4)
N(3)-C(1)-S(1)	123.0(5)
N(3)-C(1)-N(1)	125.4(6)
N(1)-C(2)-S(2)	120.8(5)
N(2)-C(2)-S(2)	123.9(5)
N(2)-C(2)-N(1)	115.3(6)
N(2)-C(3)-H(3)	108.0
N(2)-C(3)-C(4)	111.0(6)
N(3)-C(3)-N(2)	112.9(5)
N(3)-C(3)-H(3)	108.0
N(3)-C(3)-C(4)	108.8(5)
C(4)-C(3)-H(3)	108.0
C(5)-C(4)-C(3)	118.9(6)
C(9)-C(4)-C(3)	121.5(6)
C(9)-C(4)-C(5)	119.5(7)
C(4)-C(5)-H(5)	120.3
C(4)-C(5)-C(6)	119.4(7)
C(6)-C(5)-H(5)	120.3
C(5)-C(6)-H(6)	119.7
C(7)-C(6)-C(5)	120.6(8)
C(7)-C(6)-H(6)	119.7
C(6)-C(7)-H(7)	120.1
C(6)-C(7)-C(8)	119.8(8)
C(8)-C(7)-H(7)	120.1
C(7)-C(8)-H(8)	120.0
C(9)-C(8)-C(7)	120.0(9)
C(9)-C(8)-H(8)	120.0
C(4)-C(9)-C(8)	120.7(8)
C(4)-C(9)-H(9)	119.7
C(8)-C(9)-H(9)	119.7
S(1)-C(10)-H(10A)	109.5
S(1)-C(10)-H(10B)	109.5
S(1)-C(10)-H(10C)	109.5
H(10A)-C(10)-H(10B)	109.5

H(10A)-C(10)-H(10C)	109.5
H(10B)-C(10)-H(10C)	109.5

Symmetry transformations used to generate equivalent atoms:

Table S4: Anisotropic displacement parameters ($Å^2 \times 10^3$) for **6aa**. The anisotropic displacement factor exponent takes the form: $-2\pi^2[h^2 a^{*2}U^{11} + ... + 2 h k a^* b^* U^{12}]$.

	U^{11}	U^{22}	U^{33}	U^{23}	U^{13}	U^{12}
S(1)	31(1)	22(1)	31(1)	2(1)	-5(1)	9(1)
S(2)	25(1)	22(1)	23(1)	2(1)	-2(1)	4(1)
N(1)	24(3)	16(3)	31(3)	-3(2)	-4(2)	3(2)
N(2)	32(3)	18(3)	28(3)	3(2)	-6(2)	4(2)
N(3)	25(3)	25(3)	22(3)	3(2)	2(2)	7(2)
C(1)	22(3)	21(3)	15(3)	3(2)	5(2)	5(2)
C(2)	25(3)	19(3)	16(3)	2(2)	4(2)	1(2)
C(3)	23(3)	20(3)	26(3)	0(3)	-2(3)	0(2)
C(4)	31(3)	19(3)	19(3)	2(2)	-4(3)	5(3)
C(5)	44(4)	32(4)	26(4)	3(3)	6(3)	-6(3)
C(6)	57(5)	31(4)	29(4)	-3(3)	-1(4)	-6(4)
C(7)	45(5)	50(5)	31(4)	-5(4)	-1(3)	21(4)
C(8)	54(6)	74(7)	52(6)	-9(5)	25(5)	-2(5)
C(9)	44(5)	50(5)	56(6)	-13(4)	14(4)	-11(4)
C(10)	30(4)	33(4)	32(4)	7(3)	0(3)	10(3)

Table S5: Hydrogen coordinates ($\times 10^4$) and isotropic displacement parameters ($Å^2 \times 10^3$) for **6aa**.

	x	y	z	U(eq)
H(1)	7469	9329	4490	31
H(2)	7755	4995	4184	32
H(3)	3190	4827	3554	30
H(5)	1693	2711	2119	42
H(6)	2063	1066	442	50
H(7)	5274	1690	-556	52
H(8)	8327	3890	157	74
H(9)	8096	5465	1861	64
H(10A)	864	8551	1385	48
H(10B)	-546	9860	2041	48
H(10C)	-417	8230	2442	48

Table S6: Torsion angles [°] for **6aa**.

N(2)-C(3)-C(4)-C(5)	131.8(7)
N(2)-C(3)-C(4)-C(9)	-50.8(9)
N(3)-C(3)-C(4)-C(5)	-103.4(7)
N(3)-C(3)-C(4)-C(9)	74.0(9)
C(1)-N(1)-C(2)-S(2)	178.5(5)
C(1)-N(1)-C(2)-N(2)	-2.0(9)
C(1)-N(3)-C(3)-N(2)	-4.9(9)
C(1)-N(3)-C(3)-C(4)	-128.6(6)
C(2)-N(1)-C(1)-S(1)	-176.7(5)
C(2)-N(1)-C(1)-N(3)	2.8(10)
C(2)-N(2)-C(3)-N(3)	5.9(10)
C(2)-N(2)-C(3)-C(4)	128.4(7)
C(3)-N(2)-C(2)-S(2)	176.9(5)
C(3)-N(2)-C(2)-N(1)	-2.5(10)
C(3)-N(3)-C(1)-S(1)	-179.4(5)
C(3)-N(3)-C(1)-N(1)	1.1(10)
C(3)-C(4)-C(5)-C(6)	176.4(7)
C(3)-C(4)-C(9)-C(8)	-174.7(9)
C(4)-C(5)-C(6)-C(7)	-1.3(13)
C(5)-C(4)-C(9)-C(8)	2.7(15)
C(5)-C(6)-C(7)-C(8)	2.1(14)
C(6)-C(7)-C(8)-C(9)	-0.5(16)
C(7)-C(8)-C(9)-C(4)	-1.9(17)
C(9)-C(4)-C(5)-C(6)	-1.1(12)
C(10)-S(1)-C(1)-N(1)	-173.2(5)
C(10)-S(1)-C(1)-N(3)	7.3(6)

Symmetry transformations used to generate equivalent atoms:

References

1. Rezaee, P.; Davarpanah, J. *Res. Chem. Intermed.* **2016**, 42, 6815-6830. doi: 10.1007/s11164-015-2376-8
